# Direct molecular dissection of tumor parenchyma from tumor stroma in tumor xenograft using mass spectrometry-based glycoproteomics

**DOI:** 10.18632/oncotarget.25449

**Published:** 2018-05-29

**Authors:** Xiaoying Ye, Brian T. Luke, Bih-Rong Wei, Jan A. Kaczmarczyk, Jadranka Loncarek, Jennifer E. Dwyer, Donald J. Johann, Richard G. Saul, Dwight V. Nissley, Frank McCormick, Gordon R. Whiteley, Josip Blonder

**Affiliations:** ^1^ National Cancer Institute RAS Initiative, Cancer Research Technology Program, Frederick National Laboratory for Cancer Research, Leidos Biomedical Research, Inc., Frederick, MD 21702, USA; ^2^ Advanced Biomedical Computing Center, Frederick National Laboratory for Cancer Research, Leidos Biomedical Research, Inc., Frederick, MD 21702, USA; ^3^ Laboratory of Cancer Biology and Genetics, Center for Cancer Research, National Cancer Institute, Bethesda, MD 20892, USA; ^4^ Cancer Research Technology Program, Antibody Characterization Laboratory, Frederick National Laboratory for Cancer Research, Leidos Biomedical Research, Inc., Frederick, MD 21702, USA; ^5^ Laboratory of Protein Dynamics and Signaling, Center for Cancer Research, National Cancer Institute, Frederick, MD 21702, USA; ^6^ Winthrop P. Rockefeller Cancer Institute, University of Arkansas for Medical Sciences, Little Rock, AR 72209, USA; ^7^ UCSF Helen Diller Family Comprehensive Cancer Center, San Francisco, CA 94158, USA

**Keywords:** lung cancer, tumor microenvironment

## Abstract

The most widely used cancer animal model is the human-murine tumor xenograft. Unbiased molecular dissection of tumor parenchyma versus stroma in human-murine xenografts is critical for elucidating dysregulated protein networks/pathways and developing therapeutics that may target these two functionally codependent compartments. Although antibody-reliant technologies (e.g., immunohistochemistry, imaging mass cytometry) are capable of distinguishing tumor-proper versus stromal proteins, the breadth or extent of targets is limited. Here, we report an antibody-free targeted cross-species glycoproteomic (TCSG) approach that enables direct dissection of human tumor parenchyma from murine tumor stroma at the molecular/protein level in tumor xenografts at a selectivity rate presently unattainable by other means. This approach was used to segment/dissect and obtain the protein complement phenotype of the tumor stroma and parenchyma of the metastatic human lung adenocarcinoma A549 xenograft, with no need for tissue microdissection prior to mass-spectrometry analysis. An extensive molecular map of the tumor proper and the associated microenvironment was generated along with the top functional N-glycosylated protein networks enriched in each compartment. Importantly, immunohistochemistry-based cross-validation of selected parenchymal and stromal targets applied on human tissue samples of lung adenocarcinoma and normal adjacent tissue is indicative of a noteworthy translational capacity for this unique approach that may facilitate identifications of novel targets for next generation antibody therapies and development of real time preclinical tumor models.

## INTRODUCTION

Adherent two-dimensionally (2D) cultured human cancer cells are a widely used model system in cancer research and drug screening [1]. However, the biological relevance of results obtained using *in vitro* 2D-cultured cancer cell models is constrained by the lack of a natural tumor microenvironment (i.e., stroma) that provides a genuine proliferative cellular background via paracrine signals and metabolic gradients, which are fundamental and necessary for recapitulating tumor development and metastasis [2]. Hence, numerous murine xenograft models have been developed to study solid tumors in native tissue environments and to further substantiate the biology observed using 2D-cultured cancer cell lines [3].

Histologically speaking, tumor xenografts have a microanatomic configuration analogous to common solid tumors. These are characterized by the existence of two morphologically well-defined and functionally interdependent compartments: i) the parenchyma, comprising neoplastic tumor cells of diverging morphology, antigenicity, and metastatic capacity, and ii) the stroma, comprising different cellular elements including tumor fibroblasts, endothelial cells, and immune cells [4]. In human-murine tumor xenografts, the stroma is principally a product of the host (i.e., mouse), while the parenchyma consists of grafted neoplastic cells (e.g., human tumors or cell lines) [5]. During tumorigenesis, parenchymal neoplastic tumor cells secrete cytokines, growth factors, and proteolytic enzymes to sustain their survival and induce and/or modify the tumor stroma formation [6]. In this regard, any solid tumor bigger than two millimeters in diameter must induce its own blood supply, since it cannot survive without a stroma that provides a vascular network for nutrient supply and waste removal [7].

Historically, a tumor cell–centric view of cancer enabled a better understanding of tumorigenesis and facilitated the development of drugs that directly targeted tumor cells (i.e., parenchyma) using small molecules [8] or biologicals [9]. Yet, recent research efforts focused on the tumor microenvironment have shown that the tumor stroma is a legitimate anti-cancer target [10]. Consequently, the ability to analyze and differentiate tumor parenchyma from tumor stroma at the molecular level in tumor xenografts is critical for better understanding cancer biology and discovering novel and more-effective drugs/treatments that target both tumor and stroma compartments in a concurrent manner [2].

A variety of antibody-reliant technologies (e.g., immunohistochemistry, imaging mass cytometry) can be used to ascertain direct topographic distinctions between tumor parenchyma and stroma but only for a limited number of previously selected protein targets [11–13]. State-of-the-art mass spectrometry (MS)-based, antibody-free profiling of targeted tumor sections/cells relies primarily on laser capture microdissection (LCM) [14] for collecting histologically homogenous cell populations (e.g., neoplastic cells, stromal cells) prior to liquid chromatography (LC-MS)-based proteomic analysis [15, 16]. While the functional linkage between LCM and LC-MS–based proteomics enables the identification of hundreds of endogenous protein species [17–19], the optical resolution may potentially limit LCM and make the isolation of homogeneous cell populations more challenging [15]. In addition, the cost of the LCM apparatus and the obligatory requirement for a pathologist or technologist familiar with the identification/selection of targeted cells represent additional requirements [20].

Alternately, shotgun proteomics may be employed [21, 22]. However, its utility is greatly restricted due to a remarkably low dissimilarity rate (i.e., 40%) between the human and mouse MS-identifiable peptidome/proteome, which leads to exceedingly high rates of removal of peptide/protein identifications [21, 22]. This is because 60% of mouse and human MS-identifiable plain, unmodified tryptic peptides are identical (i.e., they share the same sequence). With a species specificity attribute lacking [23], MS-based discovery of viable drug targets and/or relevant biomarkers has proven to be a challenging task.

Described in this study is an antibody-free targeted cross-species N-glycoproteomic (TCSG) approach that allows for direct virtual molecular dissection/differentiation of the mouse- derived tumor microenvironment (i.e., tumor stroma) from the human-derived malignant cells (i.e., tumor parenchyma). This is performed in murine tumor xenografts at an acquisition rate presently unattainable by other means. TCSG utilizes our discovery, which showed for the first time a much higher amino acid (AA) sequence dissimilarity rate between the human and mouse MS-identifiable N-glycopeptidome compared to the corresponding plain human and mouse MS-identifiable unmodified tryptic peptidome.

To demonstrate our discovery, we adapted hydrazide-based glycoproteomics [24] to a TCSG-based proteome-wide dissection of tumor stroma from tumor parenchyma and applied it on an A549 metastatic lung adenocarcinoma tumor xenograft model. Using TCSG, we generated an extensive molecular map of the stromal and parenchymal proteome and observed unprecedented selectivity and sensitivity when differentiating tumor stroma from tumor parenchyma at the protein level. We also mapped and cross-validated the top N-glycoprotein networks activated in these two distinct co-dependent tumor compartments. The translational relevance of TCSG was confirmed by immunohistochemical (IHC) cross-validation. Here, selected parenchymal and stromal targets in matching human lung adenocarcinoma revealed a positive correlation in their location and expression compared to results obtained by TCSG in a murine lung adenocarcinoma xenograft model.

## RESULTS

### In silico computation revealed an exceedingly larger cross-species divergence/dissimilarity between the MS-identifiable N-glycosylated peptidome and the plain, unmodified peptidome

**Figure 1:**
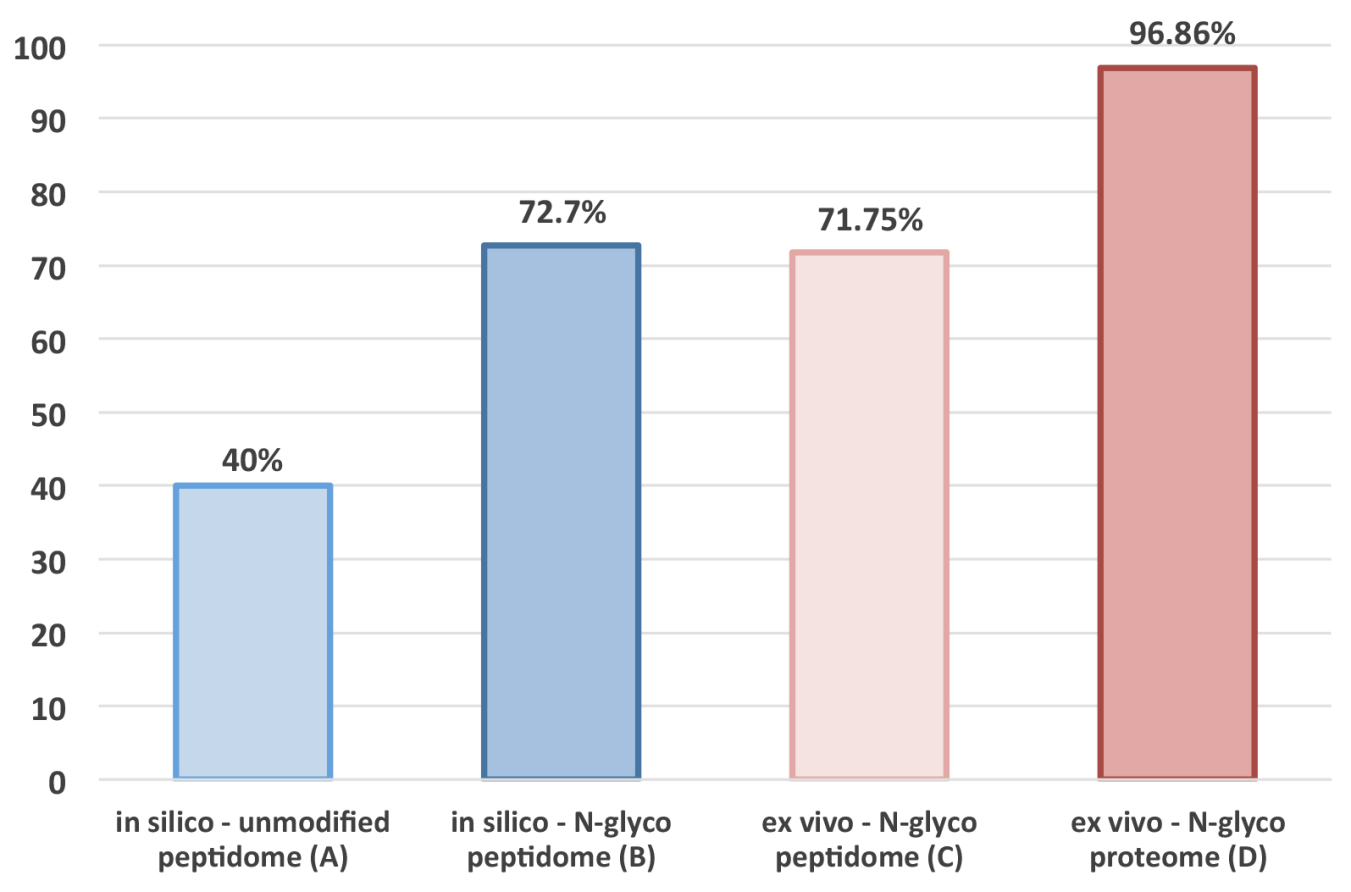
Dissimilarity rate analysis Results of *in silico* analysis **(Bars A-B)** comparing the dissimilarity rate between the human and mouse MS-identifiable plain tryptic peptidome (A) and MS-identifiable N-glycopeptidome, plus results of a corresponding *ex vivo* analysis **(Bars C-D)** comparing the observed dissimilarity rate between the human and mouse MS-identifiable N-glycopeptidome and N-glycoproteome.

In human-murine tumor xenografts, the parenchymal proteome is a product of the human neoplastic tumor cells, while the stromal proteome is principally a product of the host’s (i.e., mouse) tumor microenvironment [25]. Based on the critical role played by protein glycosylation in cancer biology [26] and self/nonself immune recognition [27], we hypothesized that the difference and/or dissimilarity rate between the human (parenchyma) and mouse (stroma) N-glycopeptidome/proteome ought to be substantially larger than the actual dissimilarity rate between the corresponding ordinary human/mouse MS-identifiable unmodified peptidome/proteome. To test our hypothesis, we performed a comparative analysis of human and mouse MS-identifiable N-glycosylated peptidome (from six AAs to 35 AAs long) *in silico* targeting the N-X-S|T motif in full protein sequences to estimate and compare differences in AA sequences between the modified MS-identifiable N-glycopeptidome and the plain MS-identifiable unmodified tryptic peptidome. The results of this *in silico* analysis showed that 75.7% of the observed mouse MS-identifiable N-glycopeptides containing the N-X-S|T amino-acid motif as well as 69.7% of the observed human MS-identifiable N-glycopeptides were distributed differently in a protein- and species-specific manner, exhibiting an average glycopeptide dissimilarity rate of 72.7% (Figure 1, Bar B).

This extensive dissimilarity rate is critical, and it enables direct molecular dissection of the tumor stroma from the tumor parenchyma by relying on species-specific differences between the parenchymal human-derived and stromal murine-derived MS-identifiable N-glycopeptidome. Evidently, only 27.3% of all MS-identifiable N-glycopeptides are shared between the human-derived and mouse-derived N-glycopeptidome, which is in striking contrast to the unmodified MS-identifiable tryptic peptides, which are 60% shared between the two species [23]. Thus, in any MS-based comparison between the human-derived parenchymal and mouse-derived stromal dataset, ∼60% of identified plain tryptic peptides should be thrown out because they are shared between two species [23], as depicted by a corresponding dissimilarity rate of only 40% (Figure 1, Bar A). Taken together, our results obtained *in silico* confirm our hypothesis and are indicative of the unparalleled potential of targeted N-glycoproteomics to enable in-depth dissection of tumor stroma from tumor parenchyma in human-murine tumor xenografts using TCSG.

### TCSG analysis of the A549 tumor xenograft recapitulates in silico computed divergence/dissimilarity between the human and mouse MS-identifiable N-glycopeptidome

**Figure 2:**
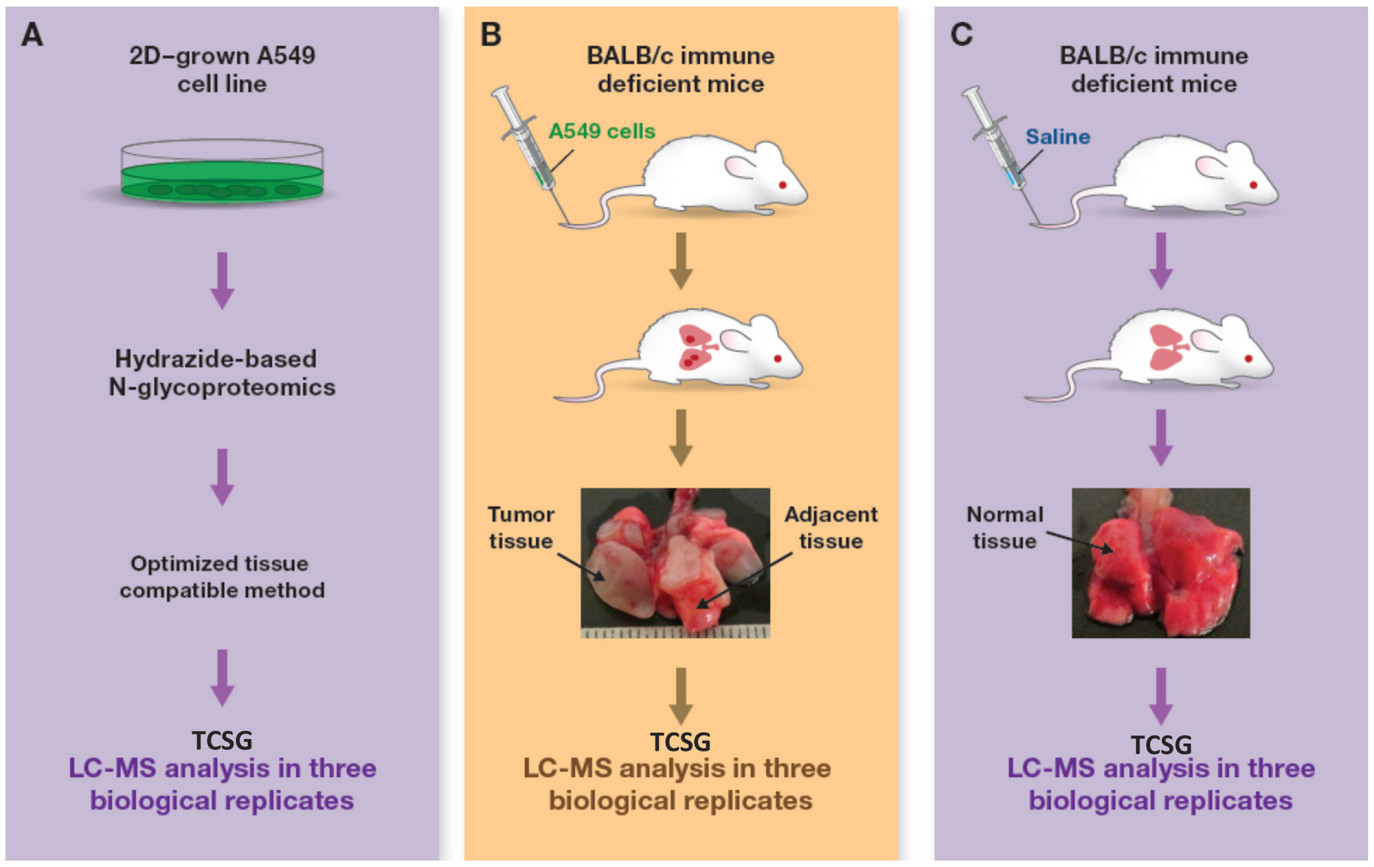
Experimental design and workflow TCSG-based molecular profiling of **(A)** tumor xenograft using **(B)** normal lung tissue and **(C)** 2D-grown A549 cell lines as controls.

The next step carried out a proof-of-principle study by applying TCSG to analyze/dissect tumor stroma from tumor parenchyma in a metastatic lung adenocarcinoma xenograft model. This was generated by injecting A549 (KRas^G12S^) human lung adenocarcinoma cells through the tail vein in nude mice as previously described [28]. Concurrently, normal mouse lung tissue collected from age- and gender-matched mice injected with normal saline and *in vitro* 2D-grown A549 cancer cells served as controls (Figure 2). To take full advantage of our *in silico* discovery and validate the hypothesis *ex vivo*, we first adopted and optimized the TCSG workflow (Supplementary Figure 1). The critical steps involved were (i) cryostat-facilitated tissue homogenization [29], (ii) aqueous-organic buffer–facilitated protein solubilization/digestion [30], (iii) N-glycosylated peptides capture [24, 31] using hydrazide-coated magnetic beads, and (iv) highly selective and sensitive cross-species N-glycoproteomic LC-MS analysis.

To ensure reliability and increase coverage, the samples were prepared on three independent occasions/repeats (i.e., biological replicates). Each sample was injected six times (i.e., technical replicates) into the high-resolution/accuracy hybrid MS and raw MS-data collated for further processing. After tissue homogenization and/or cell lysis, tryptic digestion was carried out in a mixed organic-aqueous buffer followed by oxidation of glycosylated peptides along with their covalent capture on hydrazide-coated magnetic beads. Next, non-glycopeptides were removed using stringent washes followed by the PNGase F-mediated release of N-linked glycopeptides, which were then analyzed using high-resolution and high mass accuracy LC-MS.

The measured tandem (MS^2^) spectra acquired from the tumor xenograft digestate were collated and searched against both the human and the mouse proteome databases, respectively. The MS^2^ spectra obtained from normal murine lung tissue were searched against the mouse database, while the MS^2^ spectra acquired from 2D-grown A549 human cancer cells were searched against the human proteome database. TCSG analysis of the xenograft tissue resulted in the identification of 764 human and 919 mouse N-glycopeptides (Supplementary Table 1A-1B) showing deamidated asparagine shift (0.984 Da) and N-X-S|T amino-acid motif. Correspondingly, a total of 1,263 mouse N-glycosylated peptides (Supplementary Table 2A) were identified in normal murine lungs, and 999 human N-glycosylated peptides (Supplementary Table 2B) were identified in 2D-grown A549 human lung cancer cells.

To evaluate the utility of the TCSG platform and confirm our hypothesis, we set out to compare experimentally observed dissimilarity rates in N-glyco site distributions between the human and mouse N-glycopeptidome. Towards this goal, we combined all identified peptides of human origin detected in tumor xenograft tissue and the A549 human lung cancer cell line in a single file (Supplementary Table 3A). Similarly, all identified peptides of mouse origin detected in the tumor xenograft and normal mouse lungs were combined into another file (Supplementary Table 3B). Then, each full protein sequence from the UniProt mouse proteome database was analyzed to see if it contained any of the observed/identified human N-glycopeptides. Each UniProt human protein sequence was analyzed to see if it contained any of the mouse detected peptides. The analysis revealed that 69.5% of the observed mouse N-glycosylated peptides containing the N-X-S|T amino-acid motif and 74% of the observed human N-glycosylated peptides were protein- and species-specific, exhibiting an average dissimilarity rate of 71.75% (Figure 1, Bar C).

The results of this analysis are in direct agreement with our hypothesis. They confirm the utility of TCSG to capture *ex vivo* differences between humans and mice via MS-identifiable N-glycopeptidome at the dissimilarity rate predicted *in silico.* This approach enables a direct virtual dissection of tumor parenchyma from tumor stroma in human-mouse xenografts in a species-specific manner and at a rate previously unattainable by other means. In comparison with other published approaches, the essential distinction and unparalleled selectivity of TCSG is derived directly from our discovery that revealed a much higher cross-species dissimilarity rate between human and mouse N-glycopeptidome compared to the dissimilarity rate between human and mouse plain MS-identifiable unmodified tryptic peptidome.

### TCSG enables extensive and highly selective phenotyping of the tumor stroma and tumor parenchyma at the protein level without the need for laser-based microdissection

Finally, the TCSG analysis resulted in a total of 438 mouse-stromal (Supplementary Table 4A) and 347 parenchymal-human (Supplementary Table 4B) N-glycosylated protein species identified in tumor xenograft. Importantly, a stringent false peptide discovery rate (FDR) cutoff of ≤ 1% was employed. Here, the acquired MS^2^ spectra were searched against a non-redundant Swiss-Prot human proteome database containing 20,194 protein sequences and the corresponding non-redundant Swiss-Prot mouse proteome database containing 16,299 protein sequences using the SEQUEST algorithm, which is included within the Protein Discoverer (version 1.4) software suite (Thermo). To further decrease redundancy in protein identification, protein grouping was used to generate a minimum protein list that covers all identified peptides and thereby removes redundant identifications via peptides belonging to multiple protein species. Additionally, glycoproteins identified by a single peptide matching spectrum (PMS) were not included in the final dataset. Correspondingly, a total of 471 mouse and 366 human N-glycosylated proteins were identified in normal mouse tissue and 2D-grown A549 cells, respectively (Supplementary Table 5A-5B).

These stringent searching criteria were employed to determine actual TCSG selectivity in separating/dissecting tumor stroma from tumor parenchyma at the molecular/protein level. Towards this goal, we analyzed both human and mouse protein datasets/lists to elucidate legitimate human-derived (i.e., parenchymal) from mouse-derived (i.e., stromal) proteins and/or protein groups detected in tumor xenograft. The analysis revealed that out of 438 initially identified mouse proteins and/or protein groups in tumor xenografts, a total of 427 (97.48%) were identified as genuine stromal proteins by at least a single N-glycosylated peptide in a protein- and species-specific manner (Supplementary Figure 2, Supplementary Table 6A). Correspondingly, from the 347 human proteins identified in tumor xenografts, a total of 334 (96.25%) were identified as genuine parenchymal proteins in a protein- and species-specific manner (Supplementary Figure 2, Supplementary Table 6B), corresponding to an average dissimilarity rate of 96.86% (Figure 1, Bar D). An identical analysis applied to a control normal mouse tissue and an A549 human cancer cell line produced similar results. Namely, 459 out of 472 (97.24%) mouse proteins detected in a normal mouse tissue control and 352 out of 366 proteins (96.17%) detected in 2D-grown A549 human lung adenocarcinoma cells (Supplementary Figure 2, Supplementary Table 7A-7B) were identified in a protein- and species-specific manner. These results illustrate the inherent capability of this approach to generate glycopeptide and/or glycoprotein identifications in a protein- and species-dependent manner at a rate currently unattainable by other means. It also provides explicit evidence of an unprecedent TCSG capacity for virtual molecular dissection of tumor stroma from tumor parenchyma in murine tumor xenografts without utilizing laser-based microdissection to obtain homogenous parenchymal/stromal cell populations.

### TCSG-based subtractive proteomics reveals stroma-unique mouse-derived and parenchyma-unique human-derived phenotypes

Raw tumor xenograft LC-MS data obtained by TCSG were searched against the mouse and human proteome databases, respectively. Proteins unique to mouse stroma and human parenchyma identified by at least one species-specific peptide were uncovered by subtracting overlapping protein orthologs from the original datasets. This analysis revealed a total of 211 proteins unique to tumor stroma along with a total of 118 proteins unique to tumor parenchyma (Supplementary Table 8A-8B). Notably, out of 211 identified mouse-derived stromal proteins, a total of 110 (52.13%) were annotated as authentic cell surface molecules in the cell surface protein atlas (CSPA) (Supplementary Table 8A) [32]. Of these, a total of 47 (42.72%) of the identified cell surface glycoproteins are annotated as cluster of differentiation (CD) molecules [33]. This is indicative of a significantly increased rate of CD molecules identified in tumor stroma. Interestingly, this is in comparison to an overall rate of 2% that CD molecules represent within the entire non-redundant SwissProt human/mouse proteome database. Correspondingly, out of 118 identified human-derived stromal proteins, a total of 48 (40.67%) were genuine cell surface molecules found in CSPA [32], of which a total of 15 (31.25%) were classified as CD molecules (Supplementary Table 8B). A significantly increased identification rate of CD molecules in tumor stroma is consistent with a higher content of immune cells (e.g., leukocytes) found in the tumor microenvironment (e.g., tumor-associated macrophages) and serves as another indicator of TCSG’s ability to capture interesting and timely aspects of the phenotype of the tumor microenvironment.

Next, we carried out a multiplex subtractive proteomic analysis by including control samples to elucidate mouse-derived proteins detected exclusively in tumor stroma. These identifications were below the level found in normal lung tissue and without detection of matching human-derived orthologs in tumor parenchyma or 2D-grown A549 cells. Towards this goal, we subtracted overlapping gene products detected in each dataset (e.g., tumor stroma) from the non-redundant list of collated overlapping orthologs detected in three other samples (e.g., tumor parenchyma, 2D-grown A549 cells, and normal murine lungs). This analysis revealed an exclusive detection of protein subsets/panels unique to tumor stroma (40 targets), tumor parenchyma (47 targets), 2D-grown A549 cancer cells (97 targets), and normal lung tissue (91 targets). These may be considered as putative stromal and parenchymal markers/targets critical for the biology of the tumor model under study, and they allow for a better understanding of the biology of 2D-grown A549 cells or normal lung tissue (Supplementary Table 9A-9D).

To assess the utility of multiplex subtractive proteomics, we investigated the biological processes established in the literature and reported for each of the 10 most abundant proteins detected exclusively in tumor stroma, tumor parenchyma, 2D-grown A549 cells, and normal mouse lungs, respectively. Remarkably, each of the 10 most abundant protein species (Table 1) detected exclusively in tumor stroma are known to be involved in immune processes. Of these, transmembrane glycoprotein NMB (Gpnmb), low affinity immunoglobulin gamma Fc region receptor II (Fcgr2), H-2 class I histocompatibility antigen, alpha chain, and programmed cell death 1 ligand 2 (Pdcd1lg2) are directly involved in tumor immune evasion. Conversely, the clear majority of the 10 most abundant human-derived gene products detected exclusively in tumor parenchyma (e.g., CEACAM5, MUC5B, FCGBP) were found to be involved in metastasis (Table 2). Predictably, the most abundant human-derived protein species detected exclusively in 2D-grown A549 cells (e.g., NTRK3, NRCAM, CTCS, EPHA2) involved cell proliferation (Table 3). Evidently, most of the abundant proteins exclusively detected in normal mouse lungs (Table 4) belong to “housekeeping” protein species (e.g., Abca8a, Lepr, B4gat1, Hyal2). Taken together, the results of multiplexed subtractive proteomics verify an unparalleled selectivity of TCSG-based molecular dissection. Evidently, TCSG enables mapping of the principal biological processes taking place in a sample under study. This type of subtractive analysis in the context of the present experimental design is evidently well-suited for putative drug target and biomarker discovery.

**Table 1:** Top 10 most abundant mouse-derived proteins detected solely in tumor stroma

Mouse UniProt Acc #	Gene	Protein Description	PSMs	Biological process	Reference
Q99P91	Gpnmb	Transmembrane glycoprotein NMB (DC-HIL)	344	Negative regulation of immune response	Blood. 2007 May 15;109(10):4320-7.
P08101	Fcgr2	Low affinity immunoglobulin gamma Fc region receptor II	151	Negative regulation of immune response	Immunity. 2017 Apr 18;46(4):577-586.
Q9D7Z6	Clca1	Calcium-activated chloride channel regulator 1	132	Inflammatory - immune response	PLoS One. 2013 Dec 12;8(12):e83130
Q9DBP0	Slc34a2	Sodium-dependent phosphate transport protein 2B	124	Ab-drug conjugate -cancer immunotherapy	Clin Cancer Res. 2015 Nov 15;21(22):5139-50.
P01896	N/A	H-2 class I histocompatibility antigen, alpha chain	120	Negative regulation of immune response	Bull Acad Natl Med. 2014 Apr-May;198(4-5):801-11.
P70232	Chl1	Neural cell adhesion molecule L1-like protein	67	Promotes tumorigenicity and metastasis	Clin Cancer Res. 2012 Apr 1;18(7):1914-24.
Q6R5N8	Tlr13	Toll-like receptor 13	65	Innate immune response	J Immunol. 2012 Sep 15;189(6):2717-21.
Q62192	Cd180	CD180 antigen	59	Immune response	J Exp Med. 2000 Jul 3;192(1):23-9.
Q9WUL5	Pdcd1lg2	Programmed cell death 1 ligand 2 (PD-L2)	36	Negative regulation of immune response	J Immunol. 2007 May 1;178(9):5552-62.
Q99NH8	Trem2	Triggering receptor expressed on myeloid cells 2	34	Immune response	Oncotarget. 2016 May 17;7(20):29620-34.

**Table 2:** Top 10 most abundant human-derived proteins detected solely in tumor parenchyma

Human UniProt Acc #	Gene	Protein Description	PSMs	Biological process	Reference
P06731	CEACAM5	Carcinoembryonic antigen-related cell adhesion molecule 5	1003	Metastasis	Cancer Metastasis Rev. 2013 Dec;32(3-4):643-71.
Q9HC84	MUC5B	Mucin-5B	215	Metastasis	Int J Cancer. 1996 Dec 20;69(6):457-65.
Q9Y6R7	FCGBP	IgGFc-binding protein	211	Metastasis	Oncol Lett. 2016 Jan;11(1):568-574.
Q7Z7M9	GALNT5	Polypeptide N-acetylgalactosaminyl transferase 5	150	Metastasis	Oncotarget. 2016 Aug 16;7(33):54067-54081.
Q9HD43	PTPRH	Receptor-type tyrosine-protein phosphatase H	117	Tumor growth	Nat Rev Cancer. 2011 Jan;11(1):35-49.
O75015	FCGR3B	Low affinity immunoglobulin gamma Fc region receptor III-B	105	Metastasis	Eur J Cancer. 2010 Jul;46(10):1829-34.
P98088	MUC5AC	Mucin-5AC (Fragments)	99	Enhances metastasis	Oncogene. 2016 Aug 4;35(31):4112-21.
Q12864	CDH17	Cadherin-17 (CDH17)	66	Enhances metastasis	Oncogene. 2014 Mar 27;33(13):1658-69
P01009	SERPINA1	Alpha-1-antitrypsin	54	Invasion and migration	Oncotarget. 2015 Aug 21;6(24):20312-26.
Q9UKN1	MUC12	Mucin-12	46	Tumor cell growth regulation	Cancer Res. 1999 Aug 15;59(16):4083-9.

**Table 3:** Top 10 most abundant human-derived proteins detected solely in 2D-grown A549 cells

Human UniProt Acc #	Gene	Protein Description	PSMs	Biological process	Reference
Q16288	NTRK3	NT-3 growth factor receptor	258	Increases cell proliferation	Oncogene. 2013 Aug 8;32(32):3698-710
Q92823	NRCAM	Neuronal cell adhesion molecule	197	Increases cell proliferation	Genes Dev. 2002 Aug 15;16(16):2058-72
Q13433	SLC39A6	Zinc transporter ZIP6	133	Increases cell proliferation	PLoS One. 2013;8(2):e56542.
P10586	PTPRF	Receptor-type tyrosine-protein phosphatase F	120	Increases cell proliferation	J Biol Chem. 2006 Jun 16;281(24):16482-92.
O75503	CTSC	Dipeptidyl peptidase 1	96	Growth of cultured cells	Proc Natl Acad Sci U S A. 2004 Nov 2;101(44):15724-9.
P53634	ASAH1	Acid ceramidase	89	Increases cell proliferation	J Biol Chem. 2011 Jun 3;286(22):19399-409.
Q13510	CELSR1	Cadherin EGF LAG seven-pass G-type receptor 1	79	Migration	J Neurosci. 2010 Jul 14;30(28):9392-401.
P29317	EPHA2	Ephrin type-A receptor 2	65	Increases cell proliferation	Oncogene. 2011 Dec 15;30(50):4921-9.
P13987	CD59	CD59 glycoprotein OS	64	Increases cell proliferation	Cell Immunol. 2011;272(1):61-70.
O60911	CTSV	Cathepsin L2 OS	53	Increases cell proliferation	Nat Commun. 2014 Sep 15;5:4931.

**Table 4:** Top 10 most abundant mouse-derived proteins detected solely in normal mouse lungs

Mouse UniProt Acc #	Gene	Protein Description	PSMs	Biological process	Reference
Q8K442	Abca8a	ATP-binding cassette sub-family A member 8-A	155	Lipid transport	Mamm Genome. 2003 Jan;14(1):7-20
P48356	Lepr	Leptin receptor	47	Regulation of lipid metabolism	Hepatology. 2014 Jul;60(1):133-45.
Q8BWP8	B4gat1	Beta-1,4-glucuronyltransferase 1	46	Protein glycosylation	Elife. 2014 Oct 3;3. doi: 10.7554/eLife.03941.
P08508	Fcgr3	Low affinity immunoglobulin gamma Fc region receptor III	45	Immune response regulation	J Immunol. 1998 Sep 15;161(6):3026-32.
O35632	Hyal2	Hyaluronidase-2	43	Hyaluronic acid binding	Histochem Cell Biol. 2016 Jan;145(1):53-66
Q8C6K9	Col6a6	Collagen alpha-6(VI) chain	41	Cell adhesion	J Biol Chem. 2008 Jul 18;283(29):20170-80.
Q80WV3	Chst2	Carbohydrate sulfotransferase 2	39	Inflammatory response	J Leukoc Biol. 2001 Apr;69(4):565-74.
P06339	H2-T23	H-2 class I histocompatibility antigen, D-37 alpha chain	39	Adaptive immune response	J Immunol. 2014 Aug 1;193(3):1427-39.
Q6NVD0	Frem2	FRAS1-related extracellular matrix protein 2	38	Cell adhesion	Histochem Cell Biol. 2008 Oct;130(4):785-93.
P01897	H2-L	H-2 class I histocompatibility antigen, L-D alpha chain	37	Immune systems process	Cell. 1986 Jan 31;44(2):261-72.

### Label-free relative quantitation captures a difference in the regulation of protein orthologs detected by TCSG in both tumor stroma and tumor parenchyma

We next used label-free quantitation [34, 35] to investigate and determine the differences in expression levels between stromal and parenchymal orthologs identified by at least one species-specific N-glycopeptide. The analysis revealed a total of 93 N-glycoproteins that showed a significant (p value ≤ 0.05) divergence in regulation between these two compartments (Supplementary Table 10). Of these, the most significantly upregulated molecule in tumor stroma was a gene product of integrin alpha-1 (ITGA1), and the most upregulated molecule in tumor parenchyma is a product of the CLU gene (i.e., clusterin). Both proteins were found to play significant roles in the biology of lung adenocarcinoma [36, 37]. These results indicate that the application/addition of label-free relative quantitation in the context of TCSG further expands the map of the stromal and parenchymal N-glycoproteome by capturing differences in the expression profile and phenotype of species-specific protein orthologs, which are detected in both tumor compartments with no requirement for laser-based tissue microdissection prior to LC-MS analysis.

### Bioinformatic characterization of the stromal and parenchymal proteome corroborates the fidelity/utility of TCSG-based molecular dissection

Next, we used the PANTHER classification system to characterize the stromal and parenchymal protein profiles. The protein class analysis of the stromal complement revealed an enrichment of structural and extracellular matrix proteins as well as transporters and defense/immunity proteins, while cell adhesion molecules, transferases, and hydrolases were enriched in the tumor parenchyma (Supplementary Figure 3). PANTHER pathway analysis revealed the amplification of VGEF signaling, endothelin signaling angiogenesis, chemokine/cytokine inflammation signaling, and T- and B-cell activation in the tumor stroma. The same analysis revealed cadherin signaling; EGF receptor signaling; and Wnt, Notch, and Hedgehog signaling activation in the tumor parenchyma (Supplementary Figure 4). The results of the bioinformatic molecular classification are concordant with the well-recognized roles of the tumor stroma in cancer biology (e.g., angiogenesis, immune response) as well as the roles of the parenchyma (i.e., malignant tumor cells) in tumorigenesis, as exemplified by the amplification of proliferative signals (e.g., EGF and Wnt signaling). These findings further corroborate the fidelity/utility of TCSG-based molecular dissection that enables advanced characterization and phenotyping of the tumor proper and its microenvironment.

### Pathway analysis revealed cellular movement, tumor proliferation, tumor invasion, and metastasis as the top functional networks in the tumor parenchyma

To assess the biological relevance of results obtained by virtual dissection of tumor parenchyma from the stroma and to prioritize cross-validation targets, a subset of proteins unique to and/or found upregulated in tumor parenchyma were analyzed using the Ingenuity Pathway Analysis (IPA^®^) [QIAGEN Redwood City, www.qiagen.com/ingenuity]. The IPA^®^ revealed cell migration, tumor invasion, advanced malignancy, and tumor metastasis as the top parenchymal functional networks (Supplementary Table 11A) based on statistical significance and the number of interacting protein species detected. To cross-validate the results of the IPA^®^, we set out to confirm the identification and topology of CD147, CD44, and CDH17 depicted in tumor invasion and tumor metastasis networks (Supplementary Figures 5-6).

We first selected basigin (CD147), a product of the BSG gene depicted in the tumor invasion network (Supplementary Figure 5). Other groups, including ours, have previously shown that CD147 plays an important role in the biology of KRAS-driven cancers [38–40]. Selected MS^2^ spectra depicting the identification of human-derived parenchymal CD147 via protein- and species-specific N-glycopeptides are shown in Supplementary Figures 7-8. Next, BLAST analysis compared the sequences of the human CD147 and corresponding mouse ortholog and confirmed the topology and protein/species specificity of the identified N-glycopeptides, which is underlined in red in Supplementary Figure 9.

**Figure 3:**
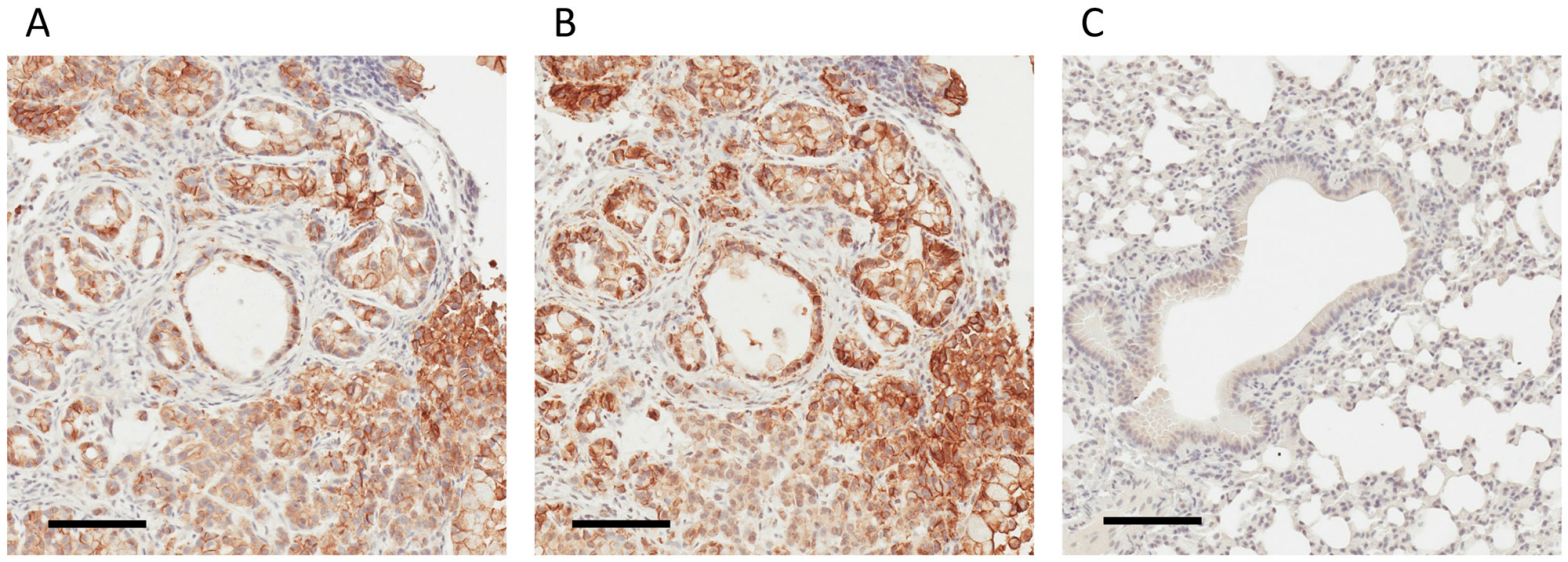
IHC-based validation of human-derived CD147 expression in tumor parenchyma **(A)** IHC staining for human-derived CD147 in tumor xenograft obtained employing the human-specific CD147 antibody. **(B)** IHC staining for human MHC I obtained employing anti-human MHC I antibody. **(C)** Absence of IHC staining in normal mouse lungs employing human-specific CD147 antibody. Bar = 100 μm.

**Figure 4:**
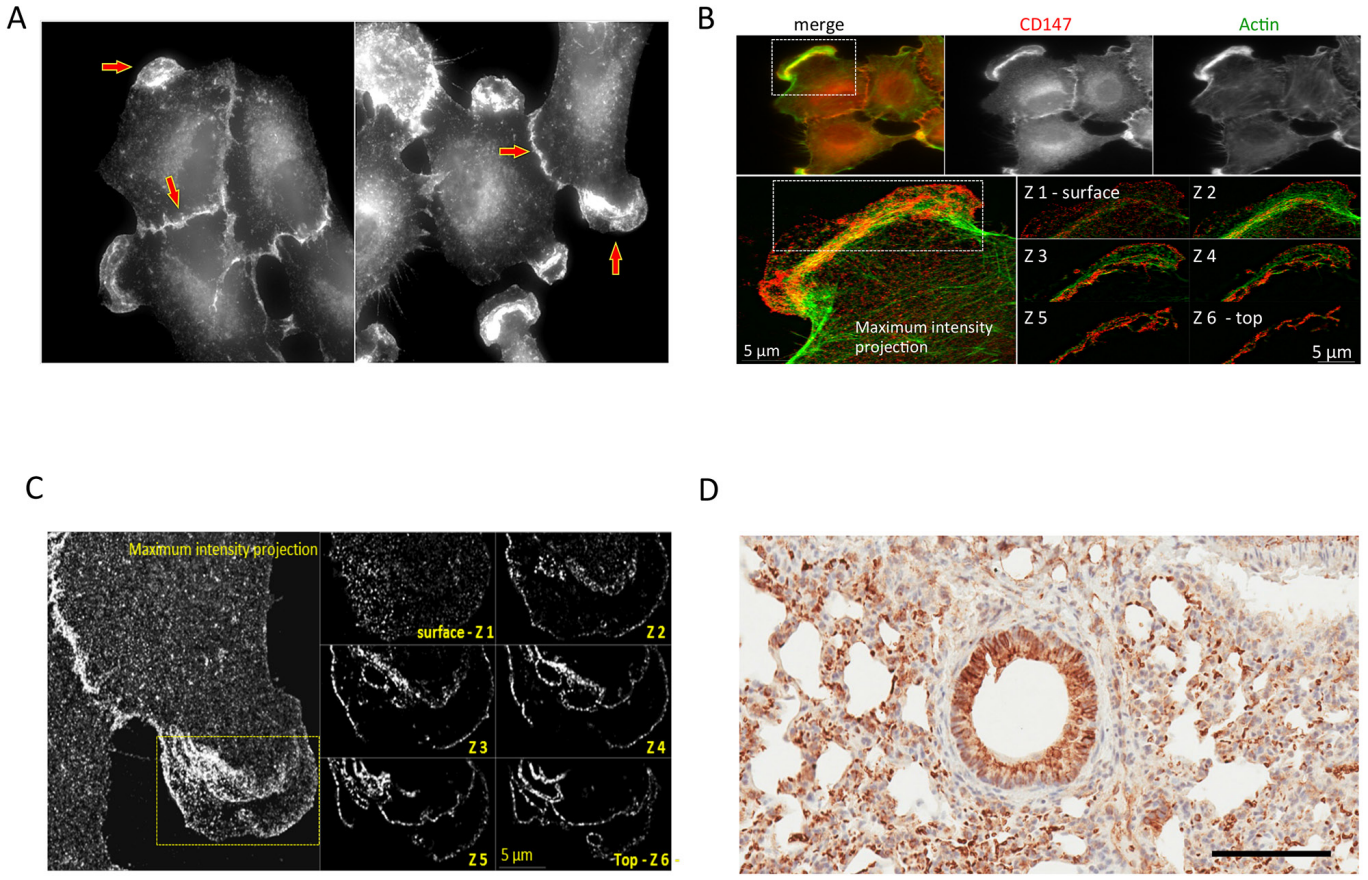
Cross-validation of the CD147 expression in human 2D-grown A549 cells and its mouse ortholog in the parenchyma of normal mouse lungs **(A)** A549 cells: wide-field fluorescence microscopy analysis of immunolabeled CD147 using anti-mouse specific antibody. **(B)** A549 cells: SIM analysis of the cell from (A) detailing localization of CD147 on cell membrane. **(C)** A549 cells: wide-field fluorescence showing colocalization of CD147 and Actin. **(D)** Normal mouse lungs: IHC staining for parenchymal mouse-derived CD147 using the mouse-specific CD147 antibody. Bar = 100 μm.

We applied IHC to analyze the tumor xenograft using a human CD147-specific antibody. The tumor xenograft IHC showed membrane staining exclusively on parenchymal cells (Figure 3A) as well as its absence in normal mouse lung tissue (Figure 3C). The human origin of parenchymal cells is confirmed by an IHC using a human MHC-I–specific antibody (Figure 3B). The expression and membrane subcellular localization of CD147 was further validated by immunofluorescence (IF) in 2D-grown A549 cells (Figure 4A-4C). The IF confirmed the expression of the human CD147 at the surface of A549 cells, and it is consistent with our previous findings showing that CD147 localizes at the surface of cancer cell lines expressing oncogenic KRas mutants endogenously [38]. Additionally, we carried out a western blot (WB) analysis of tumor tissue homogenate using a human-specific CD147 antibody that verified CD147 expression in tumor xenograft homogenate and human heart homogenate (i.e., positive control). Correspondingly, WB analysis using the same antibody was negative in normal mouse lungs and mouse heart (i.e., negative control), as shown in Supplementary Figure 10. These findings are concordant with the TCSG results and support our hypothesis.

Mouse-derived CD147 ortholog was identified in normal/healthy mouse lungs (Supplementary Table 7A) and is shown underlined in blue (Supplementary Figure 9). Human-derived CD147 (Supplementary Table 7B) was detected in 2D-grown A549 human lung adenocarcinoma cells via protein- and species-specific peptides and is shown underlined in green (Supplementary Figure 9). Depicted in Supplementary Figures 12-13 are the selected MS^2^ spectra of protein- and species-specific N-glycosylated peptides identifying mouse-derived CD147 in normal lungs (Supplementary Figure 11) and the human-derived CD147 ortholog in 2D-grown A549 cells. Next, we cross-validated the identification of the mouse-derived CD147 in normal/healthy lungs using a mouse-specific CD147 antibody. The immunostaining confirmed the expression of the mouse-derived CD147 ortholog in normal lungs, directly validating the TCSG results, and is shown in Figure 4D. In summary, these results designate CD147 as a prototypical proof-of-principle molecule that depicts unparalleled selectivity of TCSG for molecular profiling of human-mouse xenografts.

Next, we set out to investigate *in silico* if this glycopeptide-based selectivity is conserved across primate species exhibiting high similarity with the human proteome (e.g., chimpanzees). We used CD147 as a model molecule and carried out BLAST analysis to compare sequences of the human CD147 with chimpanzee and gorilla orthologs. The analysis (Supplementary Figures 14-15) showed that the human CD147 is 96.3% and 96.6% identical to chimpanzee and gorilla orthologs. Even at this high sequence similarity rate, all *in silico* predicated MS-identifiable N-glycopeptides from each of the three CD147 orthologs (i.e., human, gorilla, and chimp) were found to be species-specific identifiers. These findings suggest that the dissimilarity rate of the CD147 N-glycosylated peptides is highly conserved even across phylogenetically close species (i.e., primates) and further corroborates our hypothesis.

**Figure 5:**
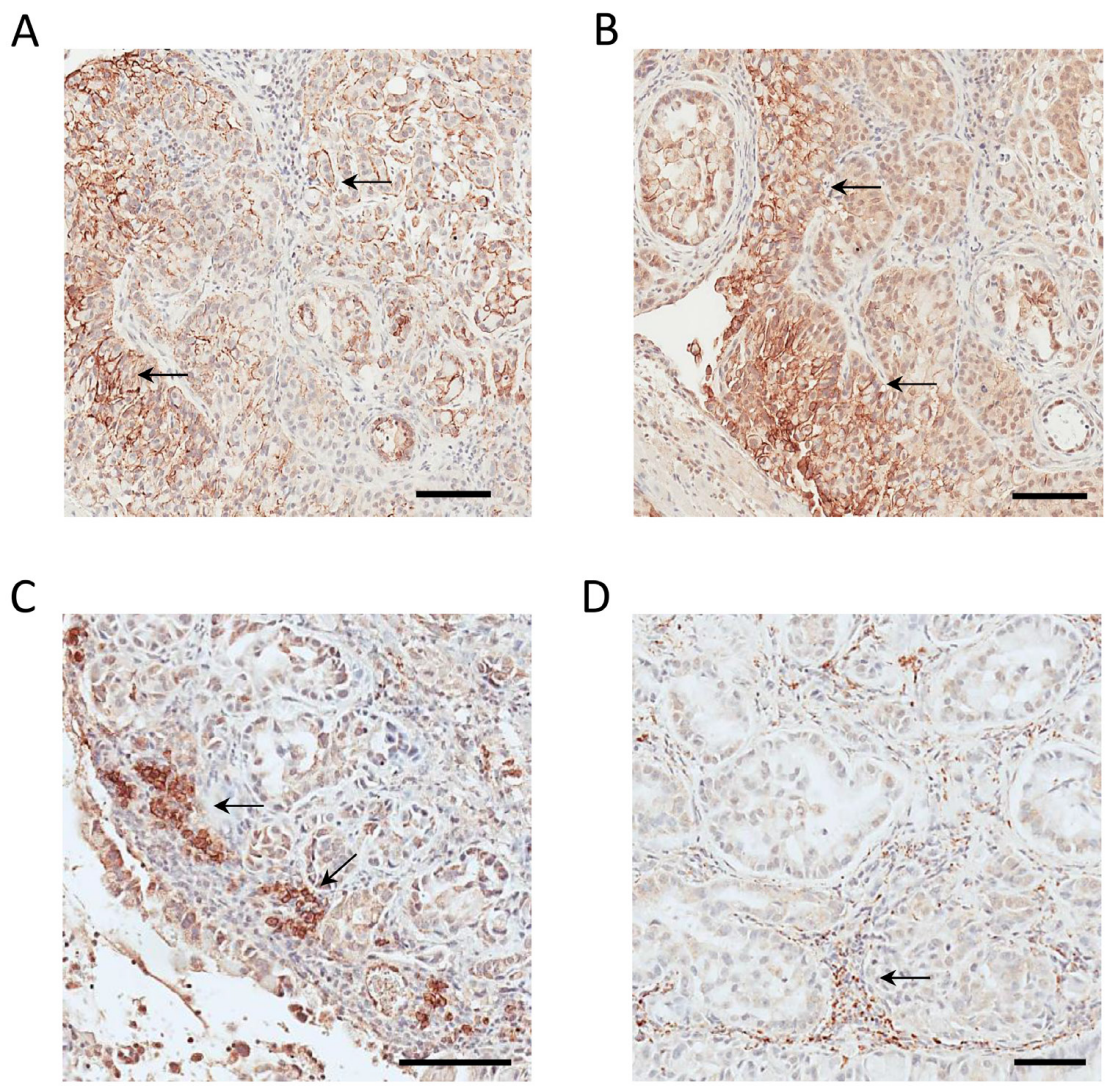
Cross-validation of parenchymal CD44 and CDH17 expression and stromal PD-L2 and ORL-1 expression in tumor xenograft tissue **(A)** Parenchymal CD44 immunostaining. **(B)** Parenchymal CDH17 immunostaining. **(C)** Stromal PD-L2 immunostaining. **(D)** Stromal LOX-1 immunostaining. Bar = 100 μm.

The CD44 antigen (CD44), depicted in the tumor invasion network (Supplementary Figure 5) was selected for orthogonal cross-validation because of its well-described role in tumorigenesis and invasion of lung adenocarcinoma driven by oncogenic KRas mutants [41, 42]. Selected MS^2^ spectra depicting the identification of human-derived parenchymal CD44 via protein- and species-specific N-glycopeptides are shown in Supplementary Figures 16-17. The BLAST analysis comparing the sequences of human CD44 and its mouse ortholog, which confirmed the topology as well as the protein and species specificity of human-derived N-glycopeptides, is underlined in red (Supplementary Figure 18). The IHC analysis unambiguously confirmed the parenchymal location of CD44 (Figure 5A). CD44 was also identified in a 2D-grown A549 cell control (Supplementary Table 5B). Notably, using A549-derived CD44^+^ cancer stem cells, Guo et al. observed an increased expression of PKM2 and proposed it as a putative drug target for lung adenocarcinoma [43]. It has also been shown that CD44 forms complexes with CD147 at the cell surface of cancer cell lines [39, 44], which is indicative of the pivotal and complex role CD147 plays in KRas-driven tumors.

Cadherin-17, depicted in the tumor metastasis network (Supplementary Figure 6), was identified by TCSG exclusively in the tumor parenchyma, with no detection in 2D-grown A549 cells or normal lung tissue. While the role of CDH17 in the biology of gastrointestinal malignancies is well documented, [45] its role in lung cancer biology has not been well established. Selected MS^2^ spectra depicting the identification of the parenchymal human-derived cadherin-17 via protein- and species-specific N-glycopeptides are shown in Supplementary Figures 19-20. The BLAST analysis comparing the sequences of human CDH17 and its mouse ortholog, which confirmed the topology as well as the protein and species specificity of the identified human-derived N-glycopeptides, is underlined in red (Supplementary Figure 21). Subsequent IHC analysis validated the localization of the human CDH17 in the tumor xenograft parenchyma (Figure 5B). Overall, the bioinformatic characterization of the molecular profiles, the pathway analyses, and the subsequent cross-validation of the selected targets all highlight the potential of TCSG technology to improve our understanding of cancer biology and facilitate the discovery of putative parenchymal biomarkers and druggable targets.

### Pathway analysis revealed leukocyte immune response, humoral immune response, cellular immune response, and movement disorder as the top functional networks in the tumor stroma

Focusing on the stromal molecular phenotype obtained by TCSG, we applied the network analysis feature of IPA^®^ on a subset of N-glycoproteins identified uniquely and/or found upregulated in the tumor stroma. The analysis revealed leukocyte immune response, humoral immune response, cellular immune response, and movement disorder as the top stromal functional networks (Supplementary Table 11B) based on statistical significance and the number of interacting N-glycoproteins identified. The outcome of the IPA^®^ is concordant with the role of the tumor stroma (i.e., tumor microenvironment) in tumor biology [46], revealing the activation of different immune response modalities that are critical for tumor immune evasion and survival. From the top protein networks, we selected the programmed cell death 1 ligand 2 (PD-L2), Pdcd1lg2 gene product, and the oxidized low-density lipoprotein receptor 1 (LOX-1)—a product of the Olr1 gene—for IHC-based cross-validation.

PD-L2 is depicted in the leukocyte immune response network (Supplementary Figure 22). The MS^2^ spectrum depicting the identification of the mouse PD-L2 ortholog in a species- and protein-specific manner is shown in Supplementary Figure 23. The BLAST analysis (Supplementary Figure 24) depicts the protein- and species-specific N-glycopeptide identifying the mouse PD-L2. The IHC staining reveals the stromal expression of PD-L2 (Figure 5C) and is found predominantly on the surface of immune cell infiltrates. While PD-L1 is still the only predictive biomarker for PD-L1/PD1 immunotherapy (i.e., nivolumab, pembrolizumab) for metastatic lung adenocarcinoma, our findings are in agreement with an increasing number of publications suggesting that additional companion IHC diagnostics targeting stromal PD-L2 may offer better prediction for targeted immunotherapy [47, 48]. Thus, PD-L2 may serve as an additional companion diagnostic marker and putative stromal target for metastatic adenocarcinoma of the lung.

LOX-1, a product of the Olr1 gene, is shown in the leukocyte migration network (Supplementary Figure 25). The representative MS^2^ spectrum of a glycopeptide identifying the mouse LOX-1 ortholog is shown in Supplementary Figure 26. The BLAST analysis confirms the protein and species specificity of the same peptide and is shown in Supplementary Figure 27. The IHC analysis confirmed explicitly the stromal LOX-1 location (Figure 5C) within the immune cell infiltrates. Unlike PD-L2, the role of LOX-1 in the biology of adenocarcinoma of the lung is poorly understood. Nonetheless, our findings are consistent with results obtained by transcriptomic analysis showing amplified LOX-1 expression in tumor-associated neutrophils in mice [49], as well as with findings showing a LOX-1–dependent enhancement of angiogenesis in prostate cancer tumor xenografts confirmed by biological assay [50].

In summary, the cross-validation verified the utility of TCSG for molecular differentiation/dissection of these two functionally and morphologically co-dependent complex tumor compartments. To the best of our knowledge, this the first study that investigated and compared the differences between human and mouse MS-identifiable N-glycopeptidomes. A TCSG approach was developed that enabled the dissection of tumor stroma from tumor parenchyma in human-murine tumor xenografts at the molecular level, along with a selectivity and specificity rate currently unattainable by other means.

### The translational relevance of the data obtained by TCSG

The translational relevance and the utility of the results obtained by the TCSG platform were evaluated by carrying out a proof-of-principle experiment focused on cross-validation of selected targets in matching human clinical tissue specimens. Several parenchymal and stromal targets detected by TCSG in the tumor xenograft were selected for IHC-based validation on human lung adenocarcinoma tissue known to contain an oncogenic KRas mutant. To mitigate the impact of natural molecular heterogeneity present at the proteome level in the human population proper, the tumor tissue and normal adjacent tissue were collected from the same patient. From the top parenchymal networks (Supplementary Table 11A), we selected two targets, CD147 and CDH17, that were found in malignant tumor invasion (Supplementary Figure 5) and tumor metastasis networks (Supplementary Figure 6), respectively. From the top stromal networks (Supplementary Table 11B), we also selected transmembrane glycoprotein NMB (DC-HIL), Gpnmb gene product, and ectonucleotide pyrophosphatase/phosphodiesterase family member 5 (NPP-5), Enpp5 gene product, that were found in stromal humoral immune response (Supplementary Figure 28) and movement disorder networks (Supplementary Figure 32), respectively.

**Figure 6:**
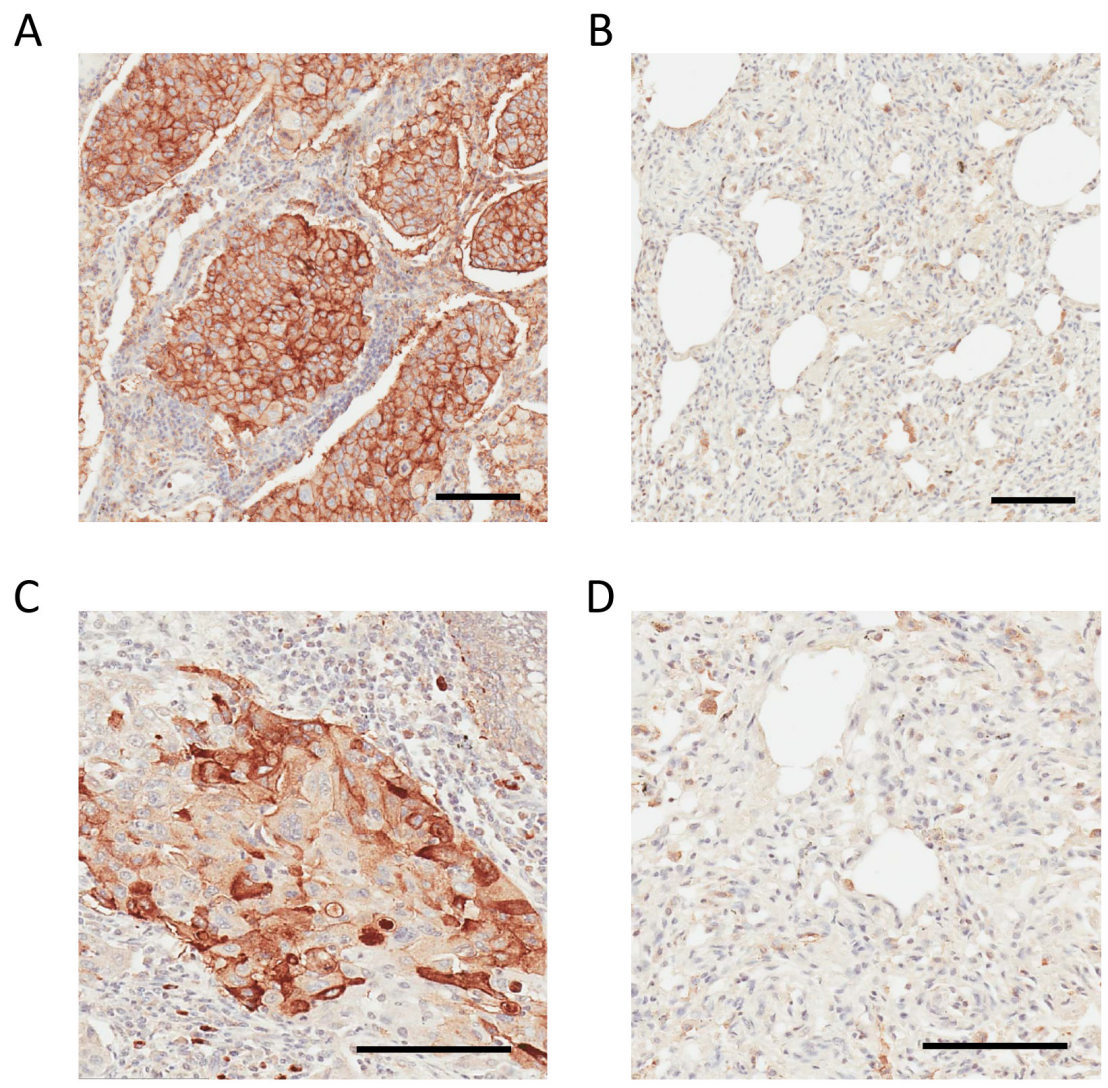
IHC analysis of CD147 and CDH17 in human lung adenocarcinoma and non-cancerous adjacent tissue **(A)** Strong positive CD147 staining in human lung adenocarcinoma. **(B)** Weak or negative CD147 staining in adjacent non-tumorous lung tissue. **(C)** Strong positive CDH17 staining in human lung adenocarcinoma. **(D)** Weak or negative CDH17 staining in adjacent non-tumorous lung tissue. Bar = 100 μm.

IHC staining of CD147 confirmed the parenchymal location in the human adenocarcinoma lung tissue. A much higher expression level of CD147 in tumor tissue compared to normal adjacent tissue (Figure 6A-6B) was observed. The role of CD147 in invasion and metastasis of human lung cancer is well described [51]. Previously, we have shown that the expression pattern of CD147 in the context of the oncogenic KRas-driven malignant transformation is highly conserved. Essentially, we showed that CD147 exhibits an identical surface expression pattern in a model of a KRas-transfected/transformed cell line (MCF10A-KRas^G12V^) as well as in pancreatic (KP3), lung (H2444), and colon (SW620) cancer cell lines expressing KRas mutants endogenously [38]. This study showed that the CD147 protein signature displays the same location at the surface of 2D-grown A549 cells in the parenchyma of a murine lung adenocarcinoma xenograft and in human lung adenocarcinoma. This is depicted in Figures 3, 4, and 6 and indicates a high translational relevance of the TCSG platform.

An intense parenchymal CDH17 staining was observed in human lung adenocarcinoma tissue (Figure 6C), and CDH17 was absent in the normal adjacent tissue (Figure 6D), which agrees with the corresponding LC-MS data obtained using TCSG (Supplementary Figures 19-20). While the role of CDH17 in lung adenocarcinoma is not well understood, our IHC findings are consistent with results obtained via tissue microarrays targeting CDH17 in patients diagnosed with metastatic colorectal adenocarcinoma [52]. Hence, CDH17 depicted in the tumor metastasis network (Supplementary Figure 6) may be added to a cytokeratin panel [53] for better classification of the metastatic human lung adenocarcinoma.

**Figure 7:**
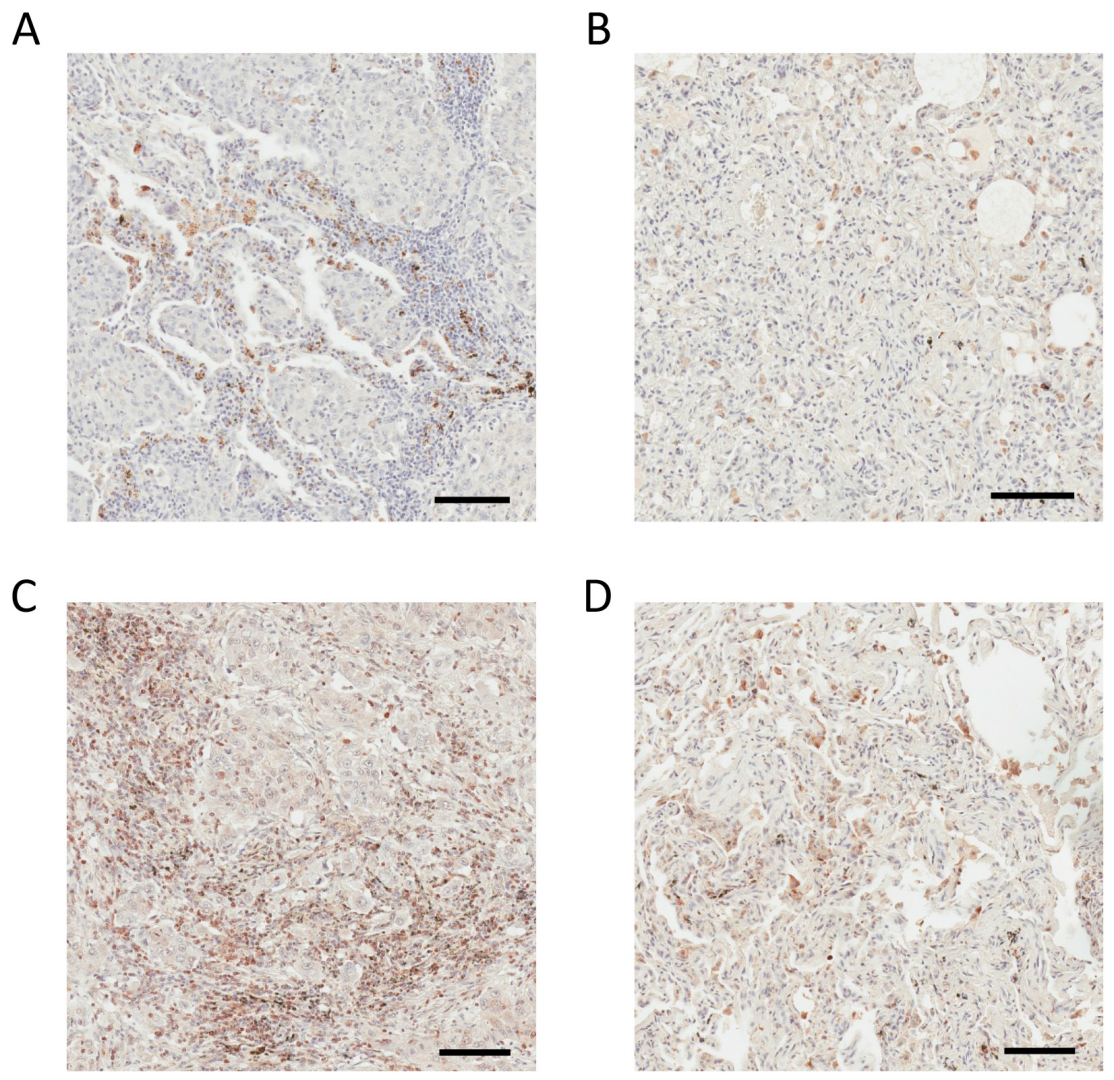
IHC analysis of DC-HIL and ENPP5 in human lung adenocarcinoma and non-cancerous adjacent tissue **(A)** Positive DC-HIL staining in stroma of human lung adenocarcinoma. **(B)** Weak or negative DC-HIL staining in adjacent non-tumorous lung tissue. **(C)** Positive ENPP5 staining in the stroma of human lung adenocarcinoma. **(D)** Weak or negative CDH17 staining in adjacent non-tumorous lung tissue. Bar = 100 μm.

DC-HIL depicted in the humoral response network (Supplementary Figure 28) is a well-described immunomodulatory molecule that plays a critical role in tumor progression and metastasis in multiple solid malignancies, including lung cancer [54]. Selected MS^2^ spectra depict the identification of the mouse DC-HIL in a species- and protein-specific manner (Supplementary Figures 29-30). The BLAST analysis (Supplementary Figure 31) verifies the protein and species specificity of the identified DC-HIL N-glycopeptides. Figure 7A shows positive stromal DC-HIL immunostaining in human lung adenocarcinoma tissue and weak or negative staining in normal adjacent lung tissue (Figure 7B). These findings are in direct agreement with LC-MS results obtained by TCSG. DC-HIL was detected as the most abundant protein unique to the tumor stroma (Table 1A). Like PD-1, DC-HIL functions as a negative regulator of T-cell activation [55]. An antibody-drug conjugate that targets DC-HIL is now in clinical trials [54].

NPP-5, depicted in the movement disorder network (Supplementary Figure 33), stimulates cell motility [56] and plays a significant role in pediatric high-grade glioma neo-angiogenesis [57]. In comparison with normal adjacent lung tissue, the stroma of human lung adenocarcinoma shows strong positive overexpression of NPP-5 in the IHC analysis (Figure 7C) and weak or negative expression in the stroma of non-tumorous adjacent lung tissue (Figure 7D). These findings are consistent with LC-MS results obtained by TCSG. The MS^2^ spectrum depicts the identification of the mouse NPP-5 in a species- and protein-specific manner (Supplementary Figure 34). The BLAST analysis verifies the protein and species specificity of the identified NPP-5 N-glycopeptides (Supplementary Figure 34). Unlike DC-HIL, the role of NPP-5 in the biology of lung cancer is poorly understood, and the present results make NPP-5 a putative target (at best) for lung adenocarcinoma therapy. These results strongly support the translational potential of TCSG and its ability to facilitate a better understanding of tumor biology as well as the discovery of putative therapeutic candidates that would enable concomitant targeting of these two codependent solid tumor compartments.

## DISCUSSION

While immune detection remains a mainstay technology (e.g., IHC, mass cytometry) in the molecular profiling of malignant tumors, its depth and scope are limited by the shortage of reliable antibodies applicable to “in-advance” selected targets. Discovery is limited. Conversely, antibody-free proteomics that relies on laser-based microdissection to separate tumor stroma from tumor parenchyma prior to LC-MS analysis is hindered by limited sample size, uncertain specimen homogeneity, tedium, and costly operator-dependent instrumentation.

Similarly, cross-species–reliant MS-based tumor xenograft proteomics, which exploits the differences between the plain/unmodified human and mouse MS-identifiable peptidome, is constrained by the fact that 60% of human and mouse MS-identifiable peptides share the same sequence (Figure 1). This greatly limits the range and capacity to differentiate between the stromal (i.e., mouse) and parenchymal (i.e., human) protein species, rendering ∼50% of peptide identifications wasted. Recent advances in cancer immunotherapy targeting malignant tumor cells (i.e., parenchymal targets) using biologicals (e.g., therapeutic antibodies) [58] or cells of the tumor microenvironment (i.e., stromal targets) using small molecules (e.g., angiogenesis inhibitors) [59] underscore a need for improved molecular diagnostic approaches. Antibody-free proteomic technologies capable of unbiased, in-depth characterization of the tumor stroma and tumor parenchyma in xenograft model systems are an advance in this direction [60].

Described in this study is a potent antibody-free MS-based approach for proteome-scale molecular dissection of tumor stroma from tumor parenchyma in murine xenografts at a differentiation rate currently unattainable by other means. The TCSG approach is discovery-based. It also allows for more selective and specific molecular mapping of these two functionally and anatomically codependent compartments. It relies primarily on our discovery showing for the first time the exceedingly higher dissimilarity rate (i.e., 72%) between mouse and human MS-identifiable N-glycopeptidomes. By comparison, the plain, unmodified MS-identifiable peptidome shows a dissimilarity rate of only 40% (Figure 1). As an illustration, BLAST analysis of the human, gorilla, and chimpanzee CD147 orthologs targeting MS-identifiable N-glycosylated peptides showed their conserved species-specific attributes despite the fact that CD147 sequences possess an identity of ≥ 96.3%. This is depicted in Supplementary Figures 15-16.

Coupling TCSG with IPA^®^ elucidated the top parenchymal tumor networks (i.e., invasion, advanced malignant tumor, and metastasis). These networks contain human-derived N-glycoproteins with an already-established linkage/role in lung adenocarcinoma signaling (e.g., CD44, CD147) and proteins whose relevance to the biology of lung adenocarcinoma is less clear (e.g., CDH17). In parallel, the IPA^®^ of the stromal N-glycoprotein complement revealed a humoral immune response, cellular immune response, and immune response of leukocytes as top immune networks. These were activated in the mouse-derived lung adenocarcinoma microenvironment depicting protein species with known roles in tumor immune evasion (e.g., PD-L2) or others whose role in the biology of lung adenocarcinoma is currently not well understood (e.g., Gpnmb). The IHC-based cross-validation of TCSG results for selected targets verified their spatial distribution within the tumor stroma and tumor parenchyma, leading to a better understanding of lung adenocarcinoma biology.

In addition, we discovered that TCSG-based molecular mapping of the tumor stroma and tumor parenchyma in mouse xenografts is further enhanced by applying subtractive proteomic analysis to control samples (i.e., 2D-grown A549 cells, normal mouse lungs). This analysis revealed a subset of N-glycoproteins exclusively detected in 2D-grown A549 cells that may be pivotal for a better understanding of Petri dish biology and/or proteins identified solely in healthy lungs that may facilitate a better understanding of respiratory processes. Alternatively, in the context of the present experimental design (Figure 2), the TCSG platform applied on the current mouse lung adenocarcinoma metastatic model may be used for rapid quantitative measurement of selected targets in a series of follow-up experiments to monitor changes in their relative concentration subsequent to experimental alterations (e.g., drug treatment).

This study begins to address the basic shortage of feasible stromal and parenchymal targets in metastatic lung adenocarcinoma mouse xenograft models. As a part of the NCI’s RAS initiative (www.cancer.gov/research/key-initiatives/ras), the Frederick National Laboratory for Cancer Research (FNLCR) utilizes MS-based proteomics to identify and characterize putative drug targets using cell lines [38, 61] and mouse models expressing oncogenic Ras mutants. FNLCR has pioneered MS-based methods for profiling clinical tissue specimens [29, 35, 62, 63] and mouse models expressing Ras mutants [64]. Using the present TCSG platform, maps were generated of 210 putative mouse-derived targets unique to the tumor stroma and 109 putative targets unique to the human-derived parenchyma. A total of 93 orthologs were found significantly dysregulated in both tumor compartments. Maps can be used as a source to refine current protein–protein interaction networks in metastatic lung adenocarcinoma. An evidence-based discovery of novel drug targets in these two co-dependent tumor compartments may also be facilitated by TCSG-generated maps.

The translational relevance of the TCSG approach was shown by cross-validating results of IHC analyses probing parenchymal (CD147, cadherin-17) and stromal targets (DC-HIL, NPP-5). This was performed using human lung adenocarcinoma and normal adjacent tissue. This suggests that the TCSG approach may be used in the initial discovery of putative lung adenocarcinoma biomarkers and/or drug targets. A limited number of clinical specimens represents a weakness of this investigation, precluding more conclusive findings. However, a recent report investigating the expression of CD147 in a total of 1,605 NSCLC patients, showed that the CD147 upregulation was positively associated with aggressive metastatic disease and shorter overall survival [65]. Nevertheless, this work is a sound proof-of-principle investigation that suggests the TCSG platform has significant translational value.

In summary, the results of this investigation authenticate the utility of the TCSG approach for facile and precise dissection of tumor stroma from tumor parenchyma in murine xenografts at a molecular specificity level previously unattainable by other means, with no need for laser-based microdissection. Illustrated here is a powerful tool that can lead to an improved understanding of cancer biology and immunity as well as the discovery of next generation antibodies aiming simultaneously and precisely at targets in tumor parenchyma and tumor stroma. Overall, the present pipeline may be employed as a robust technology in: i) drug and diagnostic target discovery, ii) therapeutic monitoring in the context of cancer immunotherapy, and iii) biology of KRas-driven cancers [66, 67]. As a complementary resource, the TCSG map/atlas described in this study can be used along with the cancer genome atlas (TCGA) to bioinformatically interrogate the tumor microenvironment and obtain an integrated view of a living tumor using the advantage of an experimental manipulation that is inaccessible in clinical settings. It is even more important in light of a recent study showing the comparative advantage of human cancer cell line models over patient-derived tumor xenograft models [68].

## MATERIALS AND METHODS

### Materials

Hydrazide-terminated 5 μm magnetic beads were purchased from Bioclone Inc. (San Diego, CA). Non-ionic Acid Labile Surfactant I (NALS I) was obtained from Protea Biosciences Group (Morgantown, WV). Tris 2-carboxyethylphosphine, Bond Breaker™ (TCEP) and iodoacetamide (IAA) were obtained from Pierce (Rockford, IL). Sequence-grade modified trypsin was obtained from Promega (Madison, WI). PNGase F was obtained from New England BioLabs (Ipswich, MA). The antibodies used for western blot and immunofluorescence analyses were from the following sources: anti-BSG was from OriGene Technologies (Rockville, MD); all other chemicals were purchased from Sigma-Aldrich (St. Louis, MO). A human lung adenocarcinoma–bearing KRas mutant and the corresponding normal adjacent tissue were obtained from an anonymous donor in the form of formalin-fixed paraffin-embedded (FFPE) tissue blocks, which were purchased from ProteoGenex, Inc. (Culver City, CA) and were accompanied by a pathologist report.

### In silico dissimilarity rate analysis

The tool getfrag.f was built and used to find all tryptic fragments between six and 35AAs for each protein with and without NxS|T motifs. For uniprot Human.fasta, the files become Human frag noNxST.txt and Human frag NxST.txt, and for uniprot Mouse.fasta, the files become Mouse frag noNxST.txt and Mouse frag NxST.txt. Each of these four files are imported into Excel and sorted by fragment. The number of total fragments between six and 35 amino acids are retained. The program fraguniq.f is then used to identify unique fragments and the protein(s) they belong to. The program/tool compfrag.f was built and used to compare the fragments.

### Cell culture

The A549 human lung adenocarcinoma cell line was purchased from A.T.C.C. (Manassas, VA) and was cultured and passaged in medium containing 10% fetal bovine serum in a cell culture incubator at 37°C in 5% CO_2_-saturated humidity conditions.

### Mouse xenografts

Mouse experiments were performed at FNLCR’s Laboratory Animal Sciences Program (LASP) in accordance with regulations from the Animal Care and Use Committee and the guidelines of the Animal Welfare Act. The metastatic lung adenocarcinoma mouse model was generated as previously described [28]. Briefly, a total of 20 SCID/NCr female mice (BALB/C background) at five weeks of age were randomly separated into two groups, experimental and control, n = 15 per group. Each mouse in the experimental group was injected with 100 μL of SF medium containing 1 × 10^6^ 2D-grown A549 cells through the tail vein. Each mouse in the control group was injected with 100 μL of SF medium. Tumor generation/growth in the experimental group was monitored via a serial magnetic resonance imaging (MRI) procedure using dual MRI mouse coils. Mice in the experimental group were euthanized upon reaching their endpoint and/or when their tumors reached a diameter ≥ 5 mm. Tumors were dissected and immediately snap-frozen in liquid nitrogen until further analysis. Correspondingly, mice in the control group were sacrificed at the same time, and the lungs were excised and snap-frozen in liquid nitrogen until further analysis. From each mouse, H&E stained FFPE tissue sections were prepared and analyzed by a LASP pathologist.

### TCSG sample preparation

Tissue samples were prepared using tip sonication to homogenize 8 μm–thick tissue slices suspended in a lysis buffer (i.e., 25 mM ammonium bicarbonate containing 1 mM PMSF) as previously described [29], followed by isolation of the crude microsomal fraction. Correspondingly, 2D-grown A549 cells were disrupted in the same lysis buffer using tip sonication, followed by isolation of the microsomal fraction as previously described [69]. The BCA assay was carried out to estimate the protein concentration in the microsomal fraction and was re-suspended in 25 mM ammonium bicarbonate using sonication. A total of 1 mg of protein was aliquoted and solubilized in 0.5% NALS I followed by sample heating at 95°C for 5 minutes. After cooling the sample, a two-step tryptic digestion was performed. In the first step, methanol was added to achieve a 10% concentration followed by a first spike of Trypsin using a 1:100 enzyme-to-substrate ratio, which was followed by incubation at 37°C for 2 hours. In the second step, additional methanol was added to achieve a 60% concentration, and a second spike of Trypsin was added to achieve a 1:50 enzyme-to-substrate ratio for an overnight digestion. Next, the digest was acidified using 20% TFA to achieve a pH level of 2 followed by incubation for 30 minutes at 37°C to cleave off NALS I. Following the incubation at 95°C for 5 minutes to inactivate trypsin, the digest was centrifuged at 10,000 x g for 5 minutes, and the supernatant was collected and lyophilized to dryness.

Next, N-glycosylated peptides were captured using a slightly modified approach that was previously described [24], which employs hydrazide-coated magnetic beads at the peptide level instead of hydrazide resin/slurry at the protein level. Briefly, the digest was resuspended in 0.1% TFA pH 5, which contained 10 mM of sodium periodate and was oxidized for 1 hour at 4°C. Sodium periodate was removed using a C18 cartridge, and the peptides were directly eluted into a vial containing hydrazide-coated magnetic beads. Glycopeptides were then conjugated to hydrazide beads by an overnight incubation at 37°C. In the next step, unconjugated peptides were removed using a series of washes with 1.5 M sodium chloride and 80% acetonitrile. Finally, N-glycopeptides were released from the magnetic beads by PNGase F during an overnight incubation at 37°C, and they were desalted using a C18 column and ultimately stored for LC-MS analysis.

### LC-MS and bioinformatic analysis

Hydrazide-captured/enriched N-glycopeptides were analyzed using nano-flow reversed phase (RP) LC-MS on the Agilent 1100 nano-flow LC system coupled on-line to an Orbitrap Elite mass spectrometer (ThermoElectron, San Jose, CA). The final N-glycopeptide mixture was reconstituted in a total of 20 μL of 0.1% TFA, which was followed by three 5 μL injections on a RP column (75 μm i.d. × 10 cm fused silica capillary with a flame-pulled tip). Finally, the mixture was slurry-packed in-house with 5 μm of 300 Å pore size C-18 stationary phase (Phenomenex, Torrance, CA). Peptides were eluted from the column using a linear gradient of 2% mobile phase B (0.1% formic acid in ACN) to 40% solvent B for 100 minutes at a flow rate of 0.25 μL/min, then to 98% B for an additional 20 minutes. The instrument was operated in a data-dependent mode using the peptide ion mass-to-charge range of 350−1800 and was monitored at the resolution level of 60,000 at m/z 400. Each MS^1^ scan was followed by 16 MS^2^ scans, wherein the 16 most abundant precursor ions were dynamically selected for collision-induced dissociation using a normalized collision energy of 35%.

Raw data obtained from the tumor xenograft were analyzed using Proteome Discoverer 1.4 (Thermo) by employing searches against the non-redundant human and mouse proteome database (SwisProt release v57.15). Raw data obtained from the normal mouse lungs and 2D-grown A549 cells were searched against the mouse and human proteome database, respectively. The following thresholds were set: for the monoisotopic peptide precursor ions (MS^1^ spectra), mass tolerance was set at 5 ppm, and for the fragment ions (data-dependent MS^2^ spectra), mass tolerance was set at 0.6 Da. Dynamic amino acid modifications were added for the detection of the following: +0.984 Da for asparagine deamidation (i.e., deamidation of N-glycosylated asparagines via PNGase F treatment), +57.021 Da for cysteine carboxyamidomethylation (i.e., alkylation), and +15.994 Da for methionine oxidation. Peptides with one tryptic terminus (K, R) meeting these thresholds were considered legitimate identifications. Finally, search results were filtered for the presence of peptides containing deamidated asparagine in the context of the N-glycosylation sequence motif (i.e., NxST) to further decrease the peptide/protein false discovery rate (FDR). N-glycosylated proteins identified by a single peptide spectrum match (PSM) were not included in the final dataset. Protein grouping was employed to increase the quality and reliability of the protein identifications and to enforce economy in the number of identified proteins. N-glycosylated proteins were characterized in accordance with their annotations in the human cell surface protein atlas (CSPA). Selection and prioritization of cell-surface proteins for antibody-based cross-validation using IHC, IF, and WB analyses were facilitated using PANTHER and IPA^®^ bioinformatic tools. Raw MS data is accessible at the https://vmsshare.nist.gov.

### Statistical analysis

Spectral counting-based relative quantitation of the changes in N-glycosylated protein regulation between the tumor parenchyma and tumor stroma was carried out using PSMs readouts, which were computed by the Percolator algorithm within Proteome Discoverer 1.4. Percolator relies on semi-supervised machine learning to improve the discrimination between correct and incorrect spectra identifications by considering p-value, q-value, and posterior error probability for each peptide match at the selected strict FDR of ≤ 0.01. Significant differences in protein regulation were estimated using binomial probability and false discovery rate (FDR) calculations [70].

### Generation of the non-redundant cell surface map of tumor parenchyma and tumor stroma

Subtractive proteomic analysis was used to reveal the non-redundant list of human-derived targets identified solely in tumor parenchyma and the mouse-derived targets identified in tumor stroma, respectively. Comparative proteomic analysis that relies on spectral counting to quantify relative changes in protein abundances was used to reveal a non-redundant list of proteins differently regulated between the tumor parenchyma and tumor stroma.

### IHC analysis

The 6 μm–thick paraffin-embedded sections of FFPE tissue were dewaxed in three changes of xylene (5 minutes each), hydrated in decreasing concentrations of ethanol (100%, 95%, and 70%), and washed in water. For antigen retrieval, slides submerged in retrieval buffer (Dako, Santa Clara, CA) were heated in a steamer for 15 minutes followed by cooling at room temperature for 15 minutes. Tissue sections were immunostained with primary antibody followed by HRP conjugated polymer secondary antibody amplification kit (DAKO) and DAB substrate. Tissue sections were then counter-stained with hematoxylin, dehydrated, and mounted with cover slips. Omission of the primary antibody served as a negative control. Images of stained tissues were obtained by scanning slides with the ScanScope XT digital slide scanner (Aperio Technologies, Inc., Vista, California) and viewed with the ImageScope program (Aperio Technologies, Inc.). The human MHC-I, CD44, and EPNN5 antibodies are from Abcam (Cambridge, MA); human CD147 is from Cell Signaling Technologies (Danvers, MA); GPNMB is from R&D Systems (Minneapolis, MN), and Cadherin 17 is from Sigma (St. Louis, MO).

### IF analysis

A549 cells were cultured on coverslips and washed with cold PBS three times, fixed in 4% formaldehyde, permeabilized with 0.1% Triton X-100, and blocked with Odyssey™ Blocking Buffer (Li-Cor, Cambridge, UK) for 1 hour. Cells were then incubated with primary antibodies overnight at 4°C, followed by incubation with Alexa Fluor 594 conjugated secondary antibodies and phalloidin-FITC (Invitrogen) for 2 hours. Cells were also stained with 4,6-diamidino-2-phenylindole (DAPI) (Invitrogen) to visualize the nuclei.

### Fluorescent microscopy

Wide-field images were acquired on a Nikon Eclipse Ti inverted microscope using a 60x NA 1.42 Plan Apo objective. The microscope was equipped with a 64 μm pixel CoolSNAP HQ^2^ camera (Photometrics) and Intensilight C-HGFIE illuminator, and 200 nm Z-sections were acquired. ImageJ (National Institutes of Health, Bethesda, MD) software was used to make maximum intensity projections and to assemble figures. Structured illumination microscopy (SIM) that relies on a grid pattern to provide higher resolution images was performed on an N-SIM (Nikon Inc.) equipped with an Apo TIRF 100x NA 1.49 Plan Apo oil objective and 405, 488, 561, and 640 nm excitation lasers as well as a back-illuminated 16 μm pixel EMCCD camera (Andor, DU897). 100 nm Z-sections were acquired in 3D SIM mode, generating 15 images per plane. Channels were corrected for chromatic shift based on the signals of 100 nm multi-spectral fluorescent spheres (TetraSpeck beads, Invitrogen) that were included in the mounting medium. For 3D visualization, we used the NIS-elements software package. To allow for comparison of the signal intensities, cells were imaged using identical imaging settings, and the images were processed identically during figure assembly.

## SUPPLEMENTARY MATERIALS FIGURES AND TABLES
























