# Supplementary material for: Direct molecular dissection of tumor parenchyma from tumor stroma in tumor xenograft using mass spectrometry-based glycoproteomics
Source: Oncotarget. 2018 May 29;9(41):26431–52. doi: 10.18632/oncotarget.25449 (PMC5995176; doi:10.18632/oncotarget.25449)
Supplement: Supplementary file 1 [file oncotarget-09-26431-s001.pdf]

# Direct molecular dissection of tumor parenchyma from tumor stroma in tumor xenograft using mass spectrometry-based glycoproteomics

## SUPPLEMENTARY MATERIALS

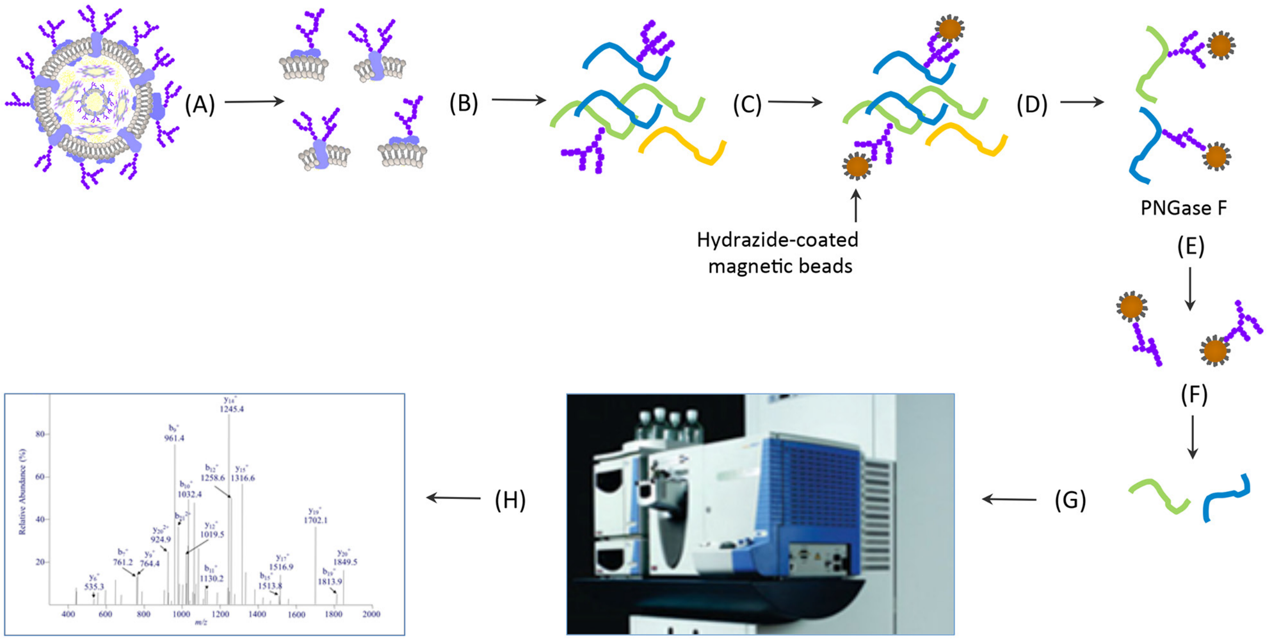


**Supplementary Figure 1: Glycoproteomic workflow.** **(A)** Tissue homogenization. **(B)** Tryptic digestion. **(C)** Oxidation and hydrazide-beads conjugation. **(D)** non-glycopeptide removal. **(E)** PNGase F based glycan removal. **(F)** N-glycopeptides collection. **(G)** LC-MS analysis. **(H)** Protein identification.

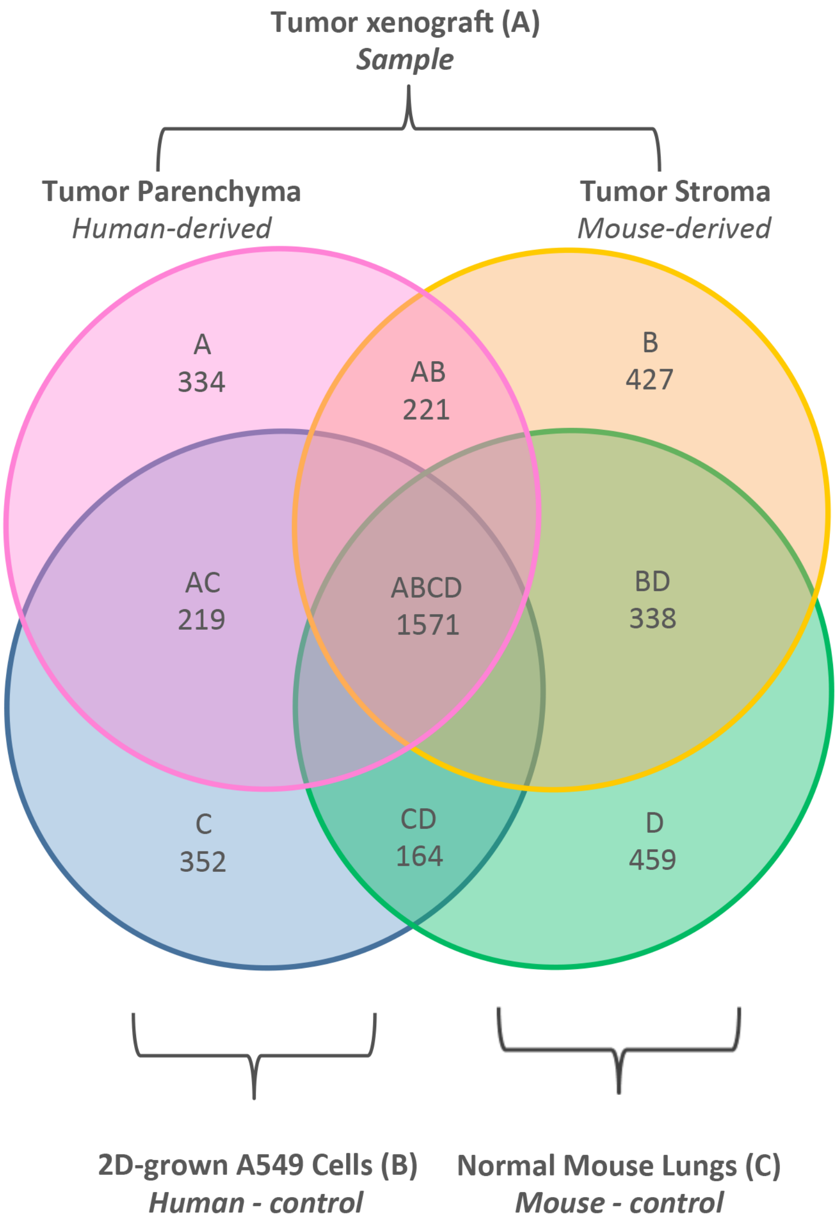


**Supplementary Figure 2: Distribution of N-glycoproteins proteins identified by at least one protein and species specific N-glycopeptide.**

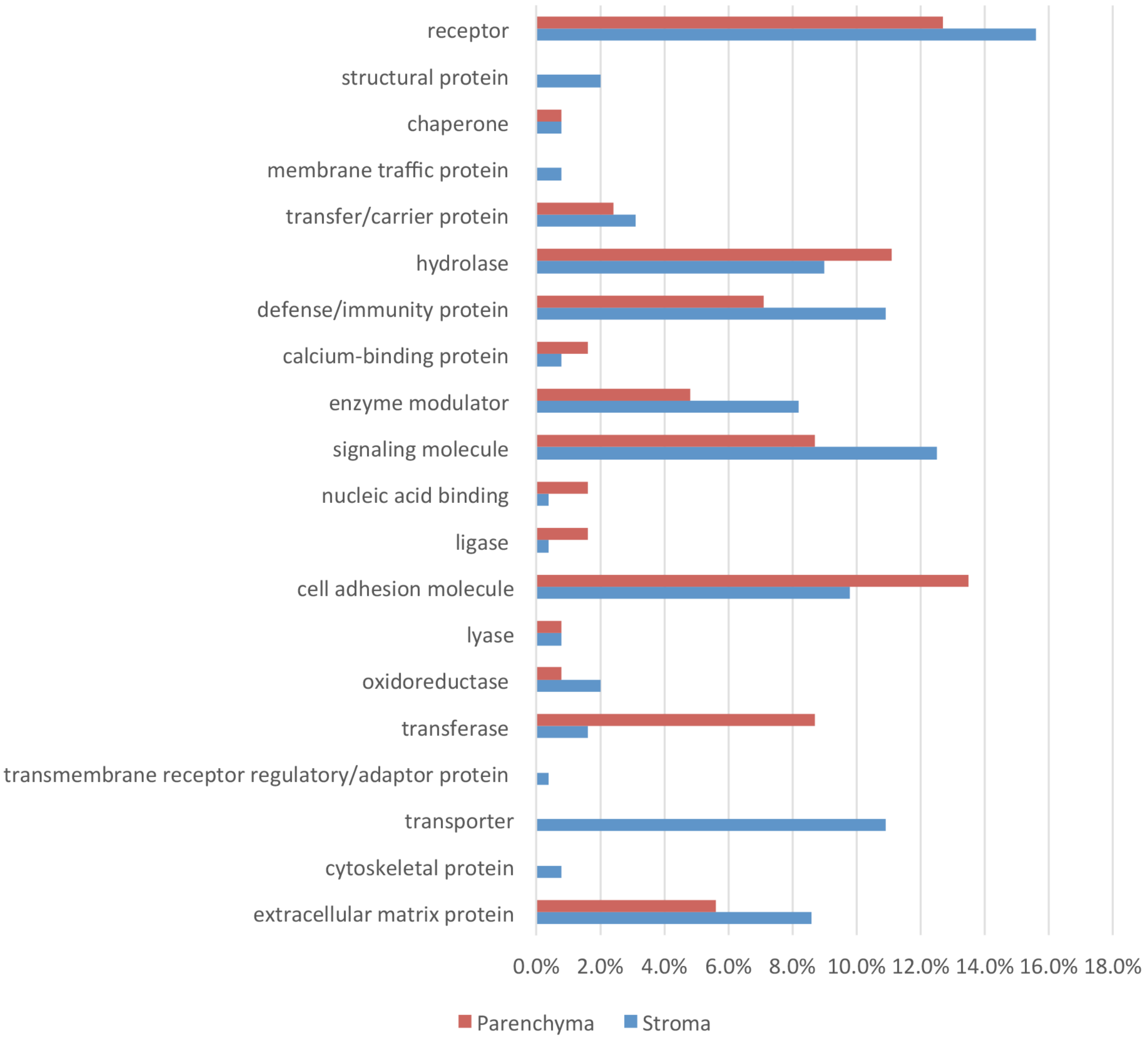


**Supplementary Figure 3: Protein class analysis using the PANTHER software.**

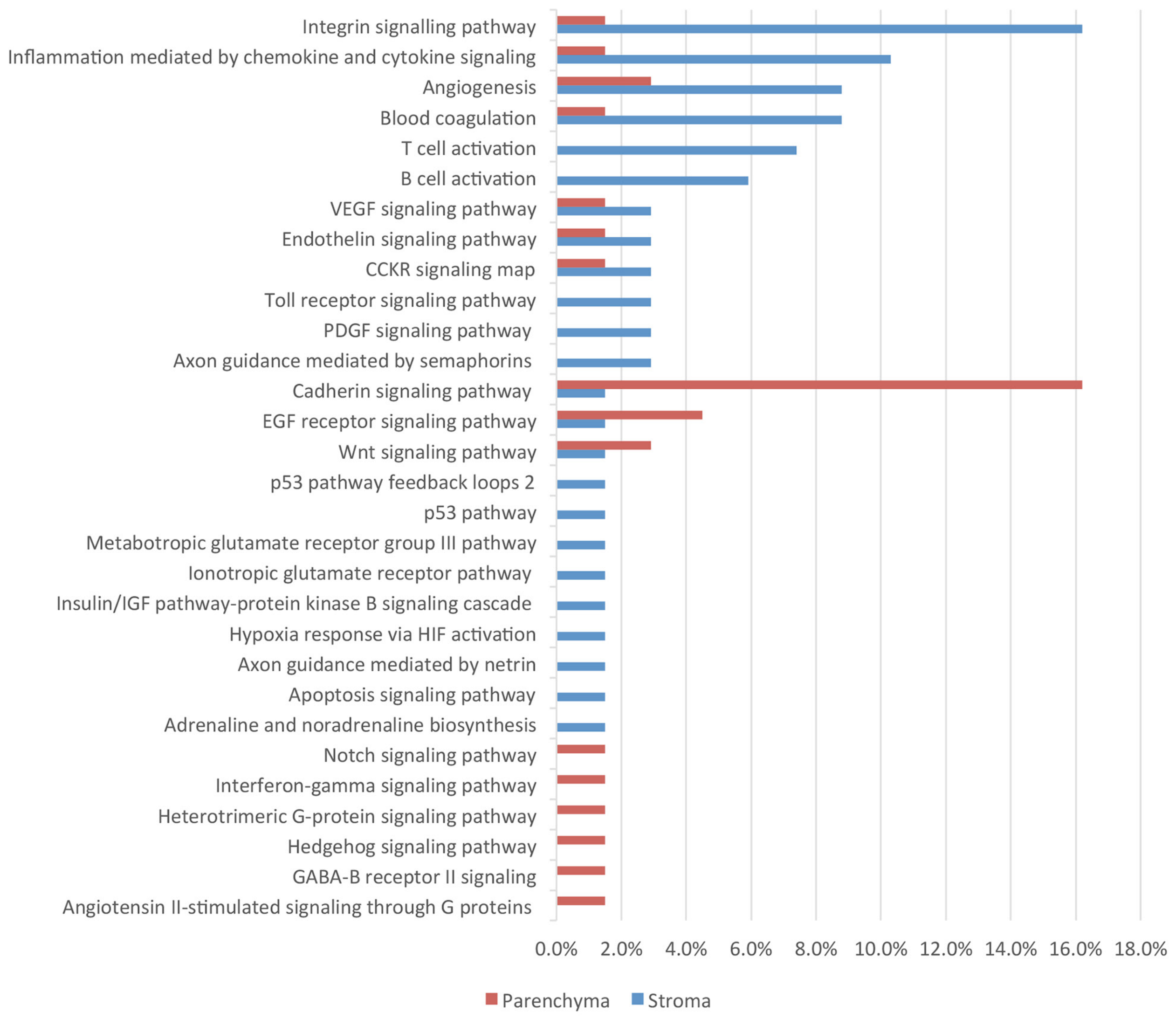


**Supplementary Figure 4: Pathway analysis using the PANTHER software.**

Network Analysis: Malignant Tumor Invasion

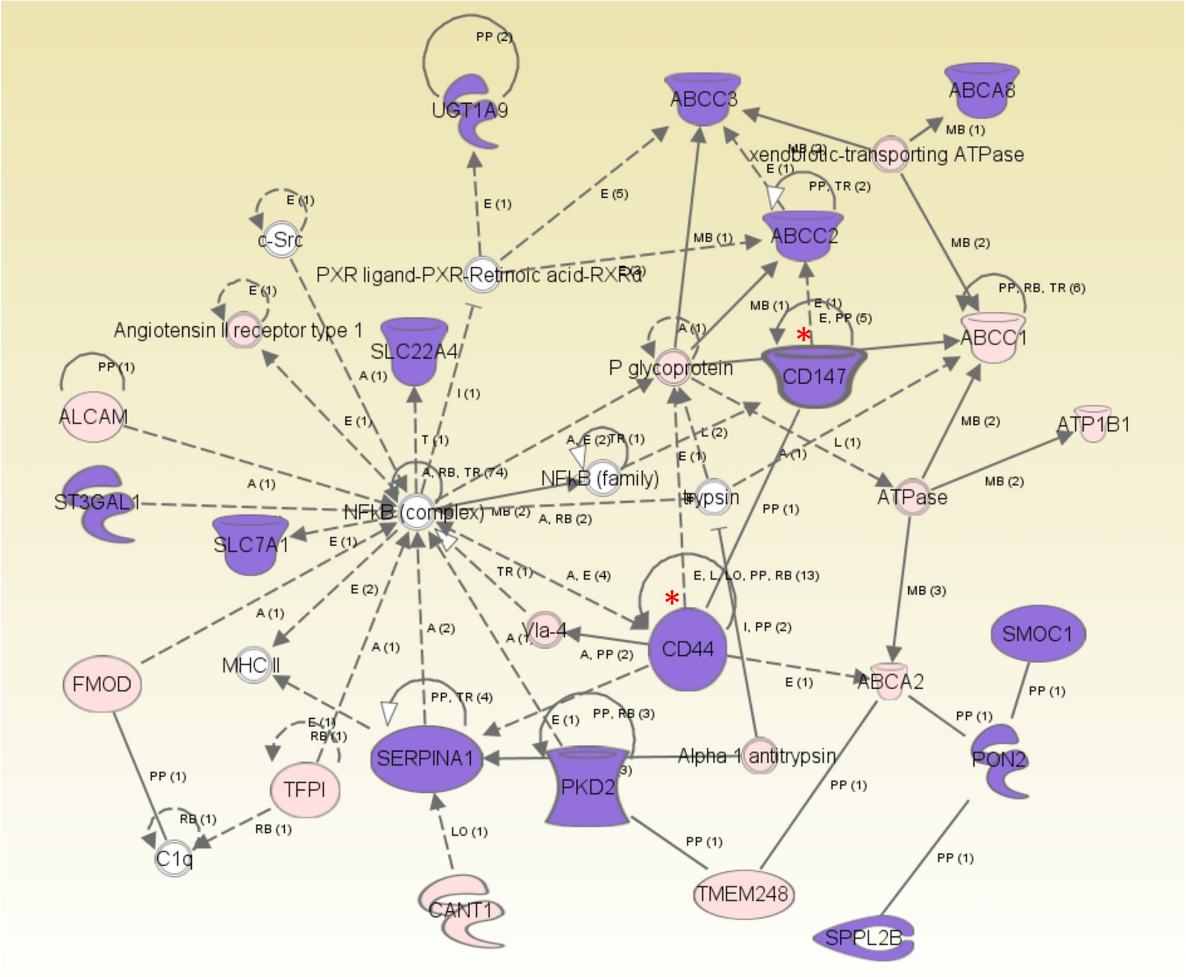


© 2000-2017 QIAGEN. All rights reserved.

**Supplementary Figure 5: IPA® analysis reveals malignant tumor invasion as one of top N-glycosylated protein networks activated in tumor parenchyma.** Molecules selected for orthogonal cross-validation (i.e., CD147 and CD44) are marked with red asterisk.

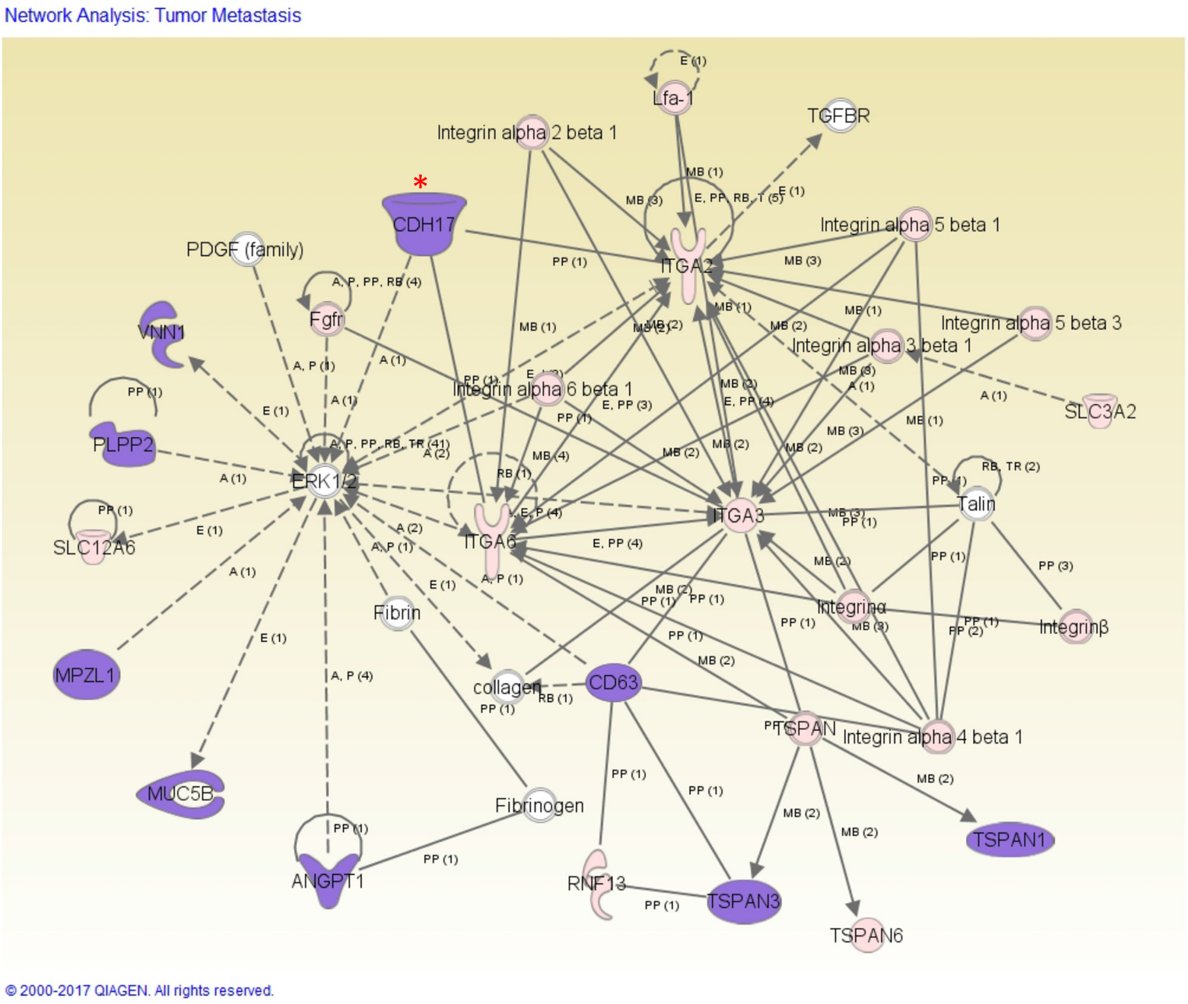


**Supplementary Figure 6: IPA® analysis reveals tumor metastasis as one of top N-glycosylated protein networks activated in tumor parenchyma.** Molecule selected for orthogonal cross-validation (i.e., CDH17) is marked with red asterisk.

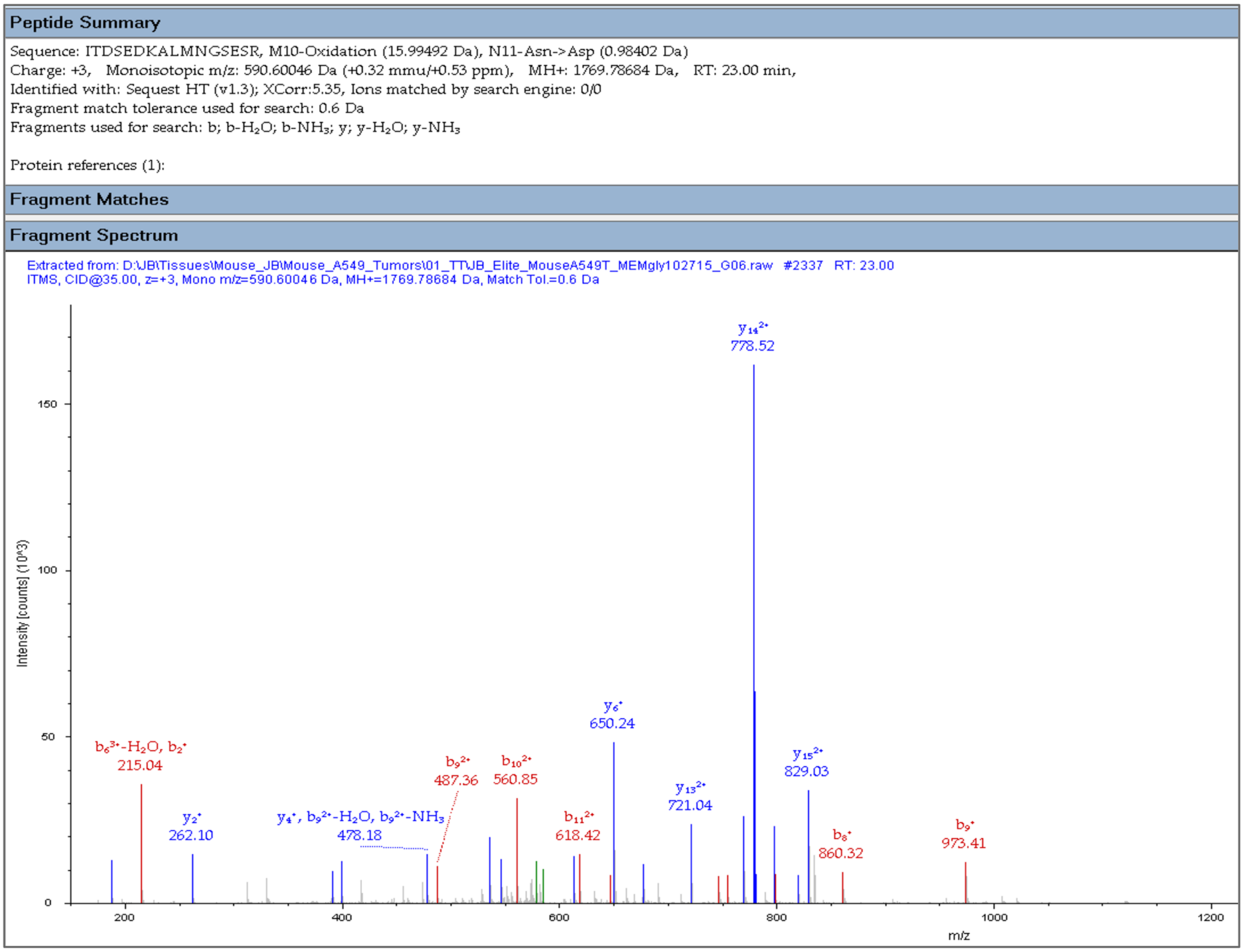


**Supplementary Figure 7: MS$^2$ spectrum depicting unambiguous identification of the human-parenchymal CD147 ortholog in tumor tissue by detection of protein- and species-specific N-glycopeptide.**

**Peptide Summary**

Sequence: ILLTCSLNDSATEVTGHR, C5-Carbamidomethyl (57.02146 Da), N8-Asn->Asp (0.98402 Da)
Charge: +3, Monoisotopic m/z: 663.33038 Da (+0.4 mmu/+0.6 ppm), MH+: 1987.97660 Da, RT: 47.19 min,
Identified with: Sequest HT (v1.3); XCorr:3.61, Ions matched by search engine: 0/0
Fragment match tolerance used for search: 0.6 Da
Fragments used for search: b; b-$H_2O$; b-$NH_3$; y; y-$H_2O$; y-$NH_3$

Protein references (1):

**Fragment Matches**

**Fragment Spectrum**

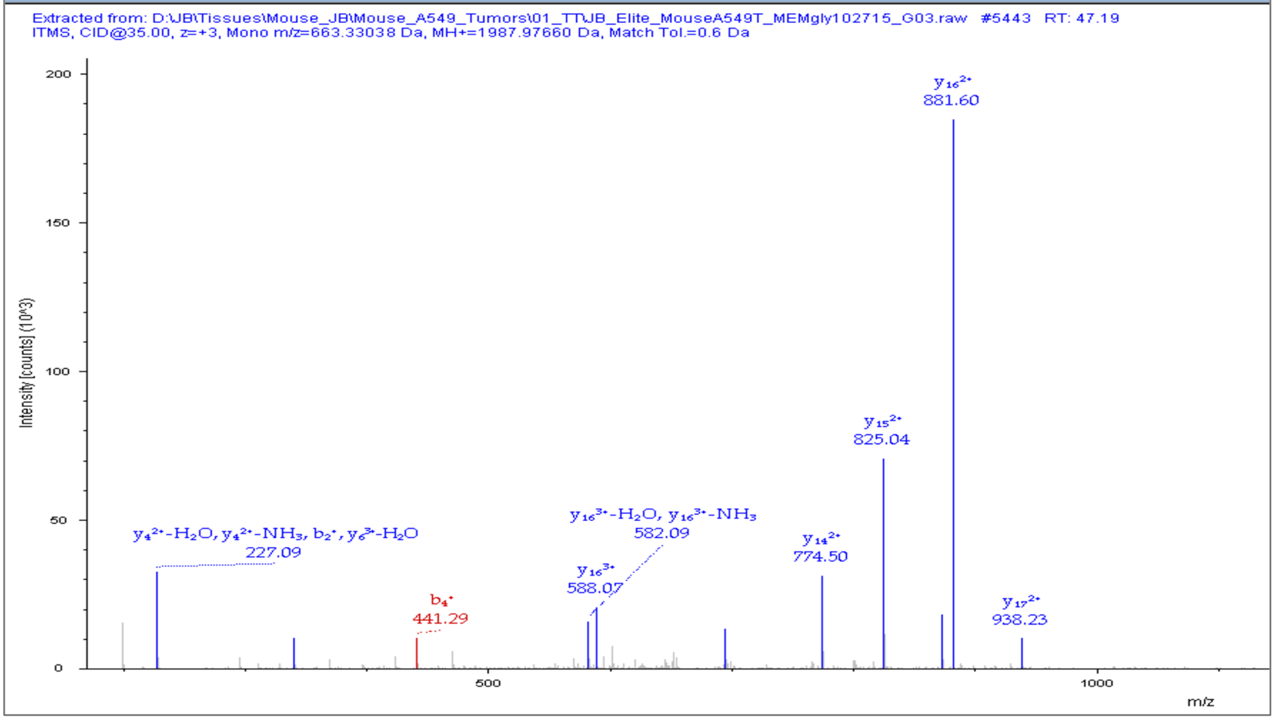


**Supplementary Figure 8: MS$^2$ spectrum depicting unambiguous identification of the human-parenchymal CD147 ortholog in tumor tissue by detection of protein- and species-specific N-glycopeptide.**

P18572 | BASI_MOUSE - **Basigin** Mus musculus (Mouse)

E-value: 0.0
Score: 1377
Ident.: 65.0%
Positives : 80.5%
Query Length: 385
Match Length: 389

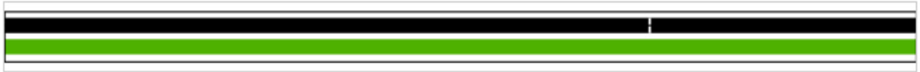

```
P35613 BASI_HUMAN     1     MAAALFVLLGFALLGTHGASGAAGFVQAPLSQQRWVGGSVELHCEAVGSPVPEIQWWFEG     60
                            MAAAL + L F LL   GA  AAGF++APLSQ+RW GGSV LHCEAVGSP+PEIQWWFEG
P18572 BASI_MOUSE     1     MAAALLLALAFTLLSGQGACAAAGFLKAPLSQERWAGGSVVLHCEAVGSPIPEIQWWFEG     60

P35613 BASI_HUMAN    61     QGPNDTCSQLWDGARLDRVHIHATYHQHAASTISIDTLVEEDTGTYECRASNDPDRNHLT    120
                              PND+CSQLWDGARLDRVHIHA Y QHAAS++S+D L  EDTGTYECRAS+DPDRNHLT
P18572 BASI_MOUSE    61     NAPNDSCSQLWDGARLDRVHIHAAYRQHAASSLSVDGLTAEDTGTYECRASSDPDRNHLT    120

P35613 BASI_HUMAN   121     RAPRVKWVRAQAVVLVLEPGTVFTTVEDLGSKILLTCSLNDSATEVTGHRWLKGGVVLKE    180
                            R PRVKWVRAQA V+VLEPGT+ T+V+++ SK  LTCSLN S  ++ GHRW++GG VL+E
P18572 BASI_MOUSE   121     RPPRVKWVRAQASVVVLEPGTIQTSVQEVNSKTQLTCSLNSSGVDIVGHRWMRGGKVLQE    180

P35613 BASI_HUMAN   181     DALPGQKTEFKVDSDDQWGEYSCVFLPEPMGTANIQLHGPPRVKAVKSSEHINEGETAML    240
                            D LP   T++ VD+DD+ GEYSC+FLPEP+G + I + GPPR+K  K SEH +EGE A L
P18572 BASI_MOUSE   181     DTLPDLHTKYIVDADDRSGEYSCIFLPEPVGRSEINVEGPPRIKVGKKSEHSSEGELAKL    240

P35613 BASI_HUMAN   241     VCKSE-SVPPVTDWAWYKITDS-EDKALMNGSES--RFFVSSSQGRSELHIENLNMEADP    296
                            VCKS+ S PP+TDW W+K +D+ E++A+ N +E+  ++ V S+  +S+L I NL++  DP
P18572 BASI_MOUSE   241     VCKSDASYPPITDWFWFKTSDTGEEEAITNSTEANGKYVVVSTPEKSQLTISNLDVNVDP    300

P35613 BASI_HUMAN   297     GQYRCNGTSSKGSDQAIITLRVRSHLAALWPFLGIVAEVLVLVTIIFIYEKRRKPEDVLD    356
                            G Y CN T+++G+ +  I+LRVRS +AALWPFLGIVAEVLVLVTIIFIYEKRRKP+  LD
P18572 BASI_MOUSE   301     GTYVCNATNAQGTTRETISLRVRSRMAALWPFLGIVAEVLVLVTIIFIYEKRRKPDQTLD    360

P35613 BASI_HUMAN   357     DDDAGSAPLKSSGQHQNDKGKNVRQRNSS                                   385
                            +DD G+APLK SG H NDK KNVRQRN++
P18572 BASI_MOUSE   361     EDDPGAAPLKGSGTHMNDKDKNVRQRNAT                                   389
```

**Supplementary Figure 9: BLAST analysis confirming protein and species specificity of identified N-glycopeptides from CD147 orthologs.** Underlined in red are human-specific peptides identified in tumor parenchyma. Underlined in green are human-specific peptides identified in 2D-grown A549 cells. Underlined in blue is mouse-specific N-glycopeptide identified in normal mouse lungs. NxST motif is highlighted in red.

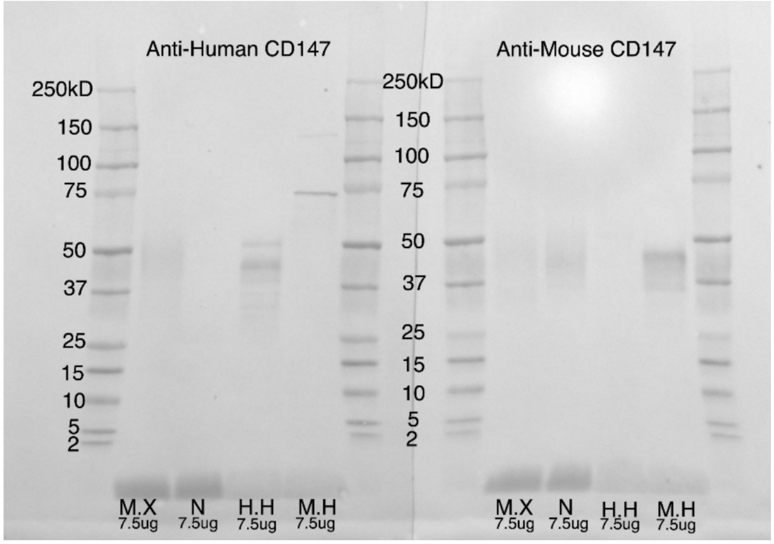


**Supplementary Figure 10: WB analysis of tumor tissue homogenate.** Using anti-human CD147 antibody the WB analysis verified expression of human-derived CD147 in tumor homogenate (MX) and human hart (HH) homogenate (i.e., positive control). The WB analysis using the same antibody was negative in normal (N) mouse lungs and mouse hart (MH) used as negative control.

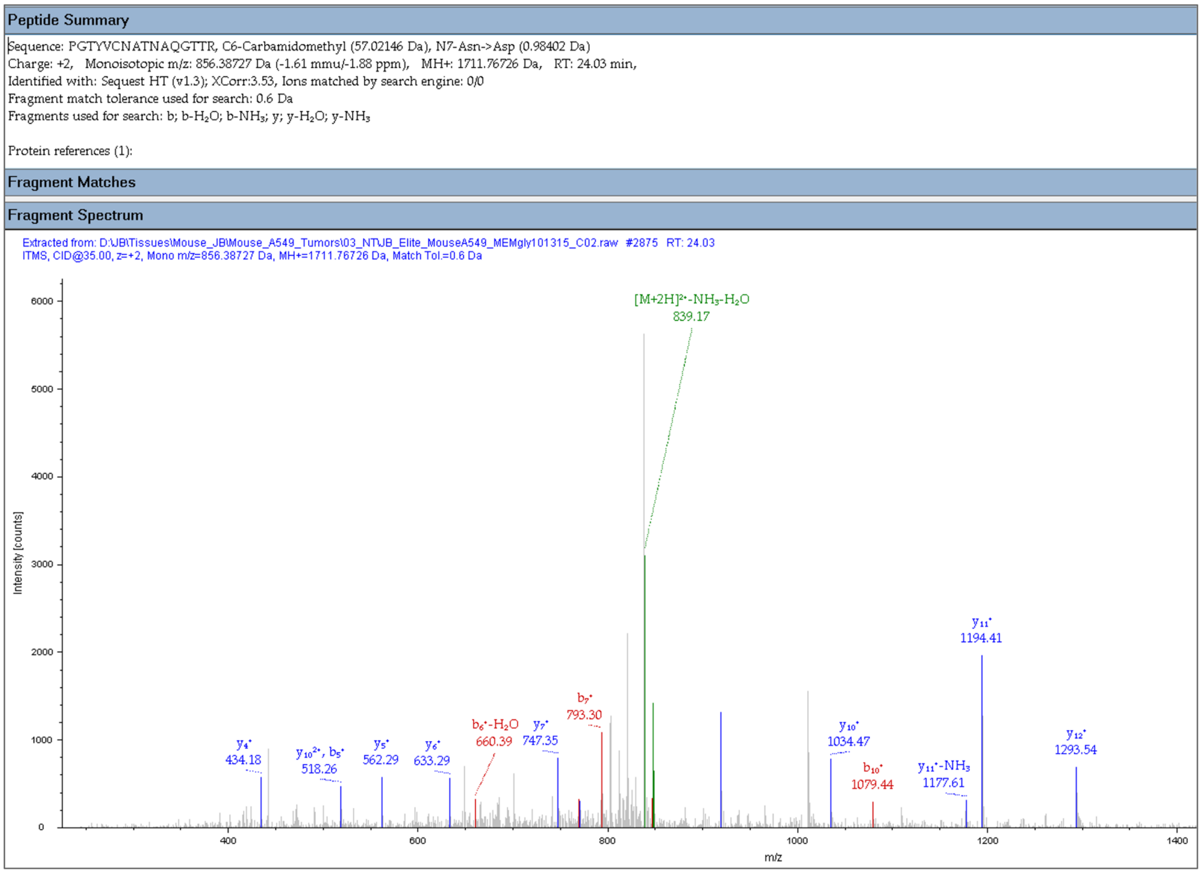


**Supplementary Figure 11: MS$^2$ spectrum depicting unambiguous identification of the mouse CD147 ortholog in normal lungs by detection of protein- and species-specific N-glycopeptide.**

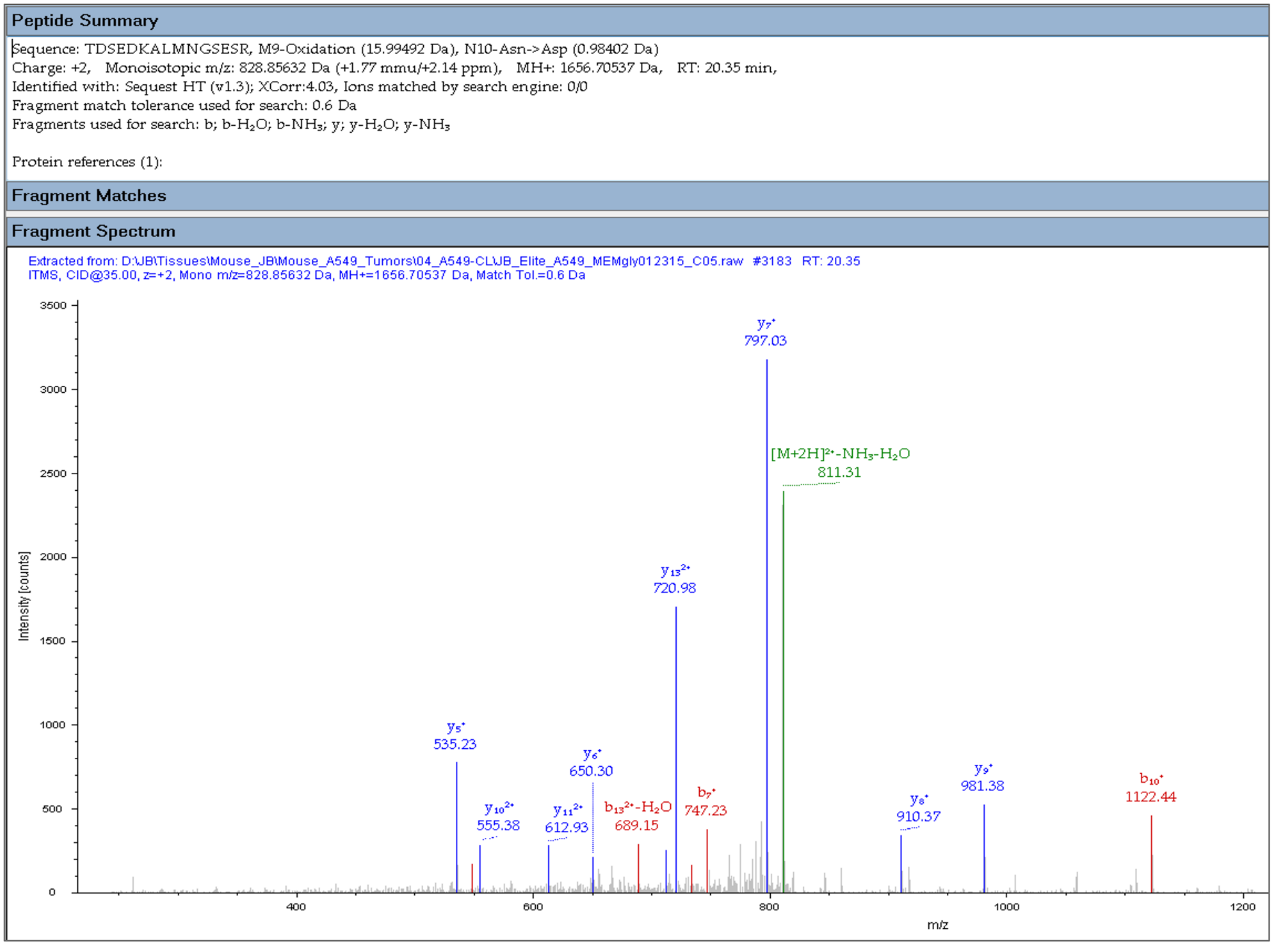


**Supplementary Figure 12: MS[2] spectrum depicting unambiguous identification of the human CD147 ortholog in 2D-grown A549 cells by detection of protein- and species-specific N-glycopeptide.**

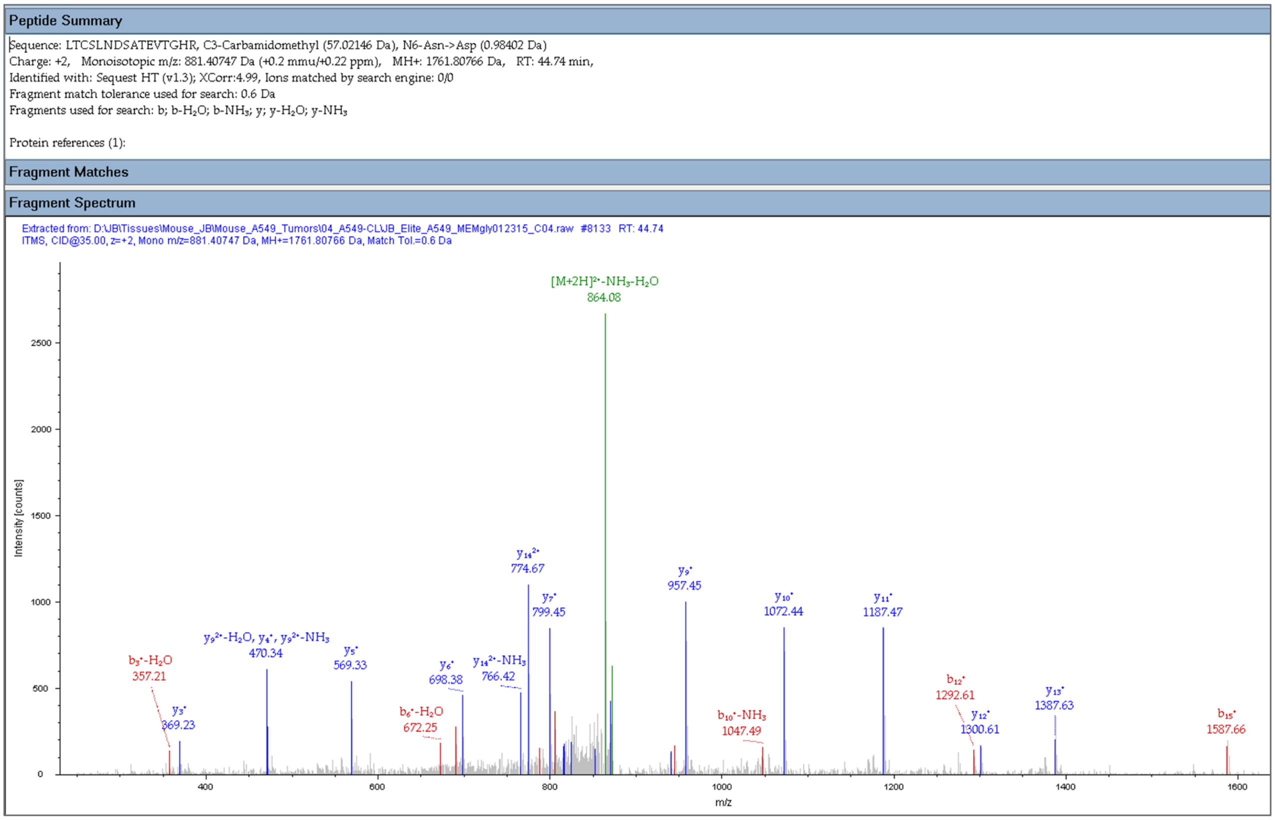


**Supplementary Figure 13: $MS^2$ spectrum depicting unambiguous identification of the human CD147 ortholog in 2D-grown A549 cells by detection of protein- and species-specific N-glycopeptide.**

G3QVG0 | G3QVG0_GORGO - **Basigin (Ok blood group)** Gorilla gorilla gorilla (Western lowland gorilla)

E-value: 0.0
Score: 1965
Ident.: 96.6%
Positives : 98.2%
Query Length: 385
Match Length: 386

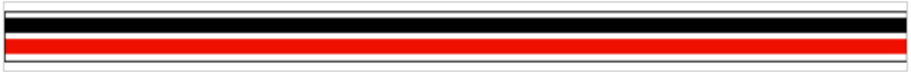

```
P35613 BASI_HUMAN      1     MAAALFVLLGFALLGTHGASGAAGFVQAPLSQQRWVGGSVELHCEAVGSPVPEIQWWFEG     60
                             MAAALFVLLGFALLGTHGASGAAGFVQAPLSQQRWVGGSVELHCEAVGSPVPEIQWWFEG
G3QVG0 G3QVG0_GORGO    1     MAAALFVLLGFALLGTHGASGAAGFVQAPLSQQRWVGGSVELHCEAVGSPVPEIQWWFEG     60

P35613 BASI_HUMAN      61    QGPNDTCSQLWDGARLDRVHIHATYHQHAASTISIDTLVEEDTGTYECRASNDPDRNHLT    120
                             QGPNDTCSQLWDGARLDRVHIHATYHQHAASTISIDTL EEDTGTYECRASNDPDRNHLT
G3QVG0 G3QVG0_GORGO    61    QGPNDTCSQLWDGARLDRVHIHATYHQHAASTISIDTLTEEDTGTYECRASNDPDRNHLT    120

P35613 BASI_HUMAN      121   RAPRVKWVRAQAVVLVLEPGTVFTTVEDLGSKILLTCSLNDSATEVTGHRWLKGGVVLKE    180
                             RAPRVKWVRAQAVVLVLEPGTV TTVE+LGSKILLTCSLNDSATEVTGHRWLKGGVVLKE
G3QVG0 G3QVG0_GORGO    121   RAPRVKWVRAQAVVLVLEPGTVLTTVEELGSKILLTCSLNDSATEVTGHRWLKGGVVLKE    180

P35613 BASI_HUMAN      181   DALPGQKTEFKVDSDDQWGEYSCVFLPEPMGTANIQL-HGPPRVKAVKSSEHINEGETAM    239
                             DALPGQKTEF+VDSDDQWGEYSCVFLPEPMGTANI+L HGPPRVKAVK SEHINEGETA+
G3QVG0 G3QVG0_GORGO    181   DALPGQKTEFEVDSDDQWGEYSCVFLPEPMGTANIKLHHGPPRVKAVKLSEHINEGETAV    240

P35613 BASI_HUMAN      240   LVCKSESVPPVTDWAWYKITDSEDKALMNGSESRFFVSSSQGRSELHIENLNMEADPGQY    299
                             L CKSESVPPVTDWAWYKITDS DKAL NGSESRFFVSSSQGRSEL+IENLNMEADPGQY
G3QVG0 G3QVG0_GORGO    241   LACKSESVPPVTDWAWYKITDSGDKALTNGSESRFFVSSSQGRSELYIENLNMEADPGQY    300

P35613 BASI_HUMAN      300   RCNGTSSKGSDQAIITLRVRSHLAALWPFLGIVAEVLVLVTIIFIYEKRRKPEDVLDDDD    359
                             RCNGTSSKGSDQA+ITLRVRSHLAALWPFLGIVAEVLVLVTIIFIYEKRRKPEDVLDDDD
G3QVG0 G3QVG0_GORGO    301   RCNGTSSKGSDQAVITLRVRSHLAALWPFLGIVAEVLVLVTIIFIYEKRRKPEDVLDDDD    360

P35613 BASI_HUMAN      360   AGSAPLKSSGQHQNDKGKNVRQRNSS                                      385
                             AGSAPLKSSGQHQNDKGKNVRQRNSS
G3QVG0 G3QVG0_GORGO    361   AGSAPLKSSGQHQNDKGKNVRQRNSS                                      386
```

**Supplementary Figure 14: BLAST analysis depicting comparison of human's and gorilla's CD147 orthologs.** Highlighted in red are predicted N-glycosylated sites showing protein and species-specific AA sequence differences of MS-identifiable N-glycopeptides.

G2HDX7 | G2HDX7_PANTR - **Basigin** Pan troglodytes (Chimpanzee)

E-value: 1.5e-168
Score: 1247
Ident.: 96.3%
Positives : 98.4%
Query Length: 385
Match Length: 269

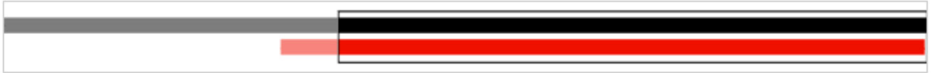

```
P35613 BASI_HUMAN     140   GTVFTTVEDLGSKILLTCSLNDSATEVTGHRWLKGGVVLKEDALPGQKTEFKVDSDDQWG    199
                            GTVFTTVEDLGSKILLTC +NDSATEVTGHRWLKGGVVL+EDALPGQKTEFKVDSDDQWG
G2HDX7 G2HDX7_PANTR   24    GTVFTTVEDLGSKILLTCYMNDSATEVTGHRWLKGGVVLEEDALPGQKTEFKVDSDDQWG     83

P35613 BASI_HUMAN     200   EYSCVFLPEPMGTANIQLHGPPRVKAVKSSEHINEGETAMLVCKSESVPPVTDWAWYKIT    259
                            EYSCVFLPEPMGTANIQLHGPPRVKAVKSSEHINEGETA+LVCKSESVPPVTDWAWYKIT
G2HDX7 G2HDX7_PANTR   84    EYSCVFLPEPMGTANIQLHGPPRVKAVKSSEHINEGETAVLVCKSESVPPVTDWAWYKIT    143

P35613 BASI_HUMAN     260   DSEDKALMNGSESRFFVSSSQGRSELHIENLNMEADPGQYRCNGTSSKGSDQAIITLRVR    319
                            DS DKALMNGSESRFFVSS QGRSELHIENLNMEADPGQYRCNG SS+GSDQA+ITLRVR
G2HDX7 G2HDX7_PANTR   144   DSGDKALMNGSESRFFVSSLQGRSELHIENLNMEADPGQYRCNGNSSEGSDQAVITLRVR    203

P35613 BASI_HUMAN     320   SHLAALWPFLGIVAEVLVLVTIIFIYEKRRKPEDVLDDDDAGSAPLKSSGQHQNDKGKNV    379
                            SHLAALWPFLGIVAEVLVLVTIIFIYEKRRKPEDVLDDDDAGSAPLKSSGQHQNDKGKNV
G2HDX7 G2HDX7_PANTR   204   SHLAALWPFLGIVAEVLVLVTIIFIYEKRRKPEDVLDDDDAGSAPLKSSGQHQNDKGKNV    263

P35613 BASI_HUMAN     380   RQRNSS                                                          385
                            RQRNSS
G2HDX7 G2HDX7_PANTR   264   RQRNSS                                                          269
```

**Supplementary Figure 15: BLAST analysis depicting comparison of human's and chimpanzee's CD147 orthologs.** Highlighted in red are predicted N-glycosylated sites showing protein and species-specific AA sequence differences of MS-identifiable N-glycopeptides.

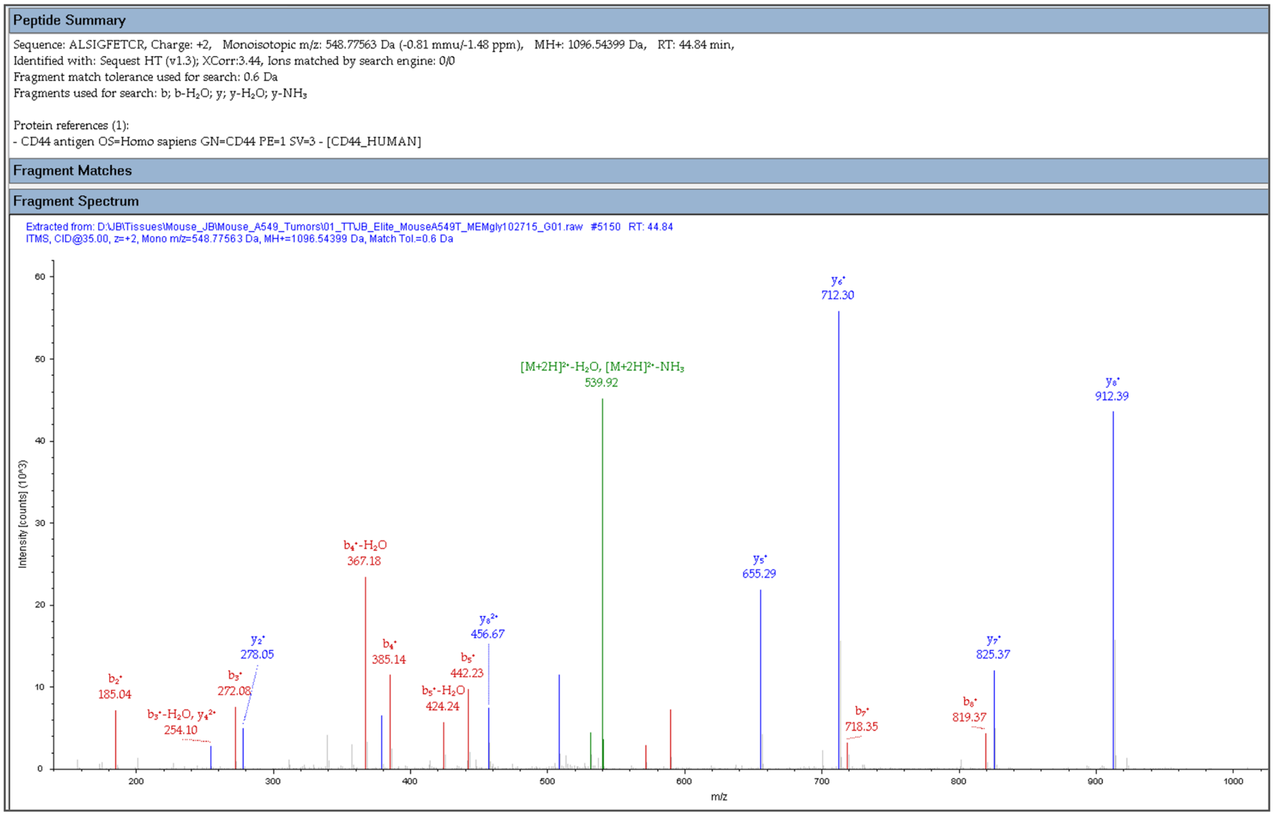


**Supplementary Figure 16: MS$^2$ spectrum depicting unambiguous identification of the human-derived CD44 ortholog in tumor parenchyma by detection of protein- and species-specific N-glycopeptide.**

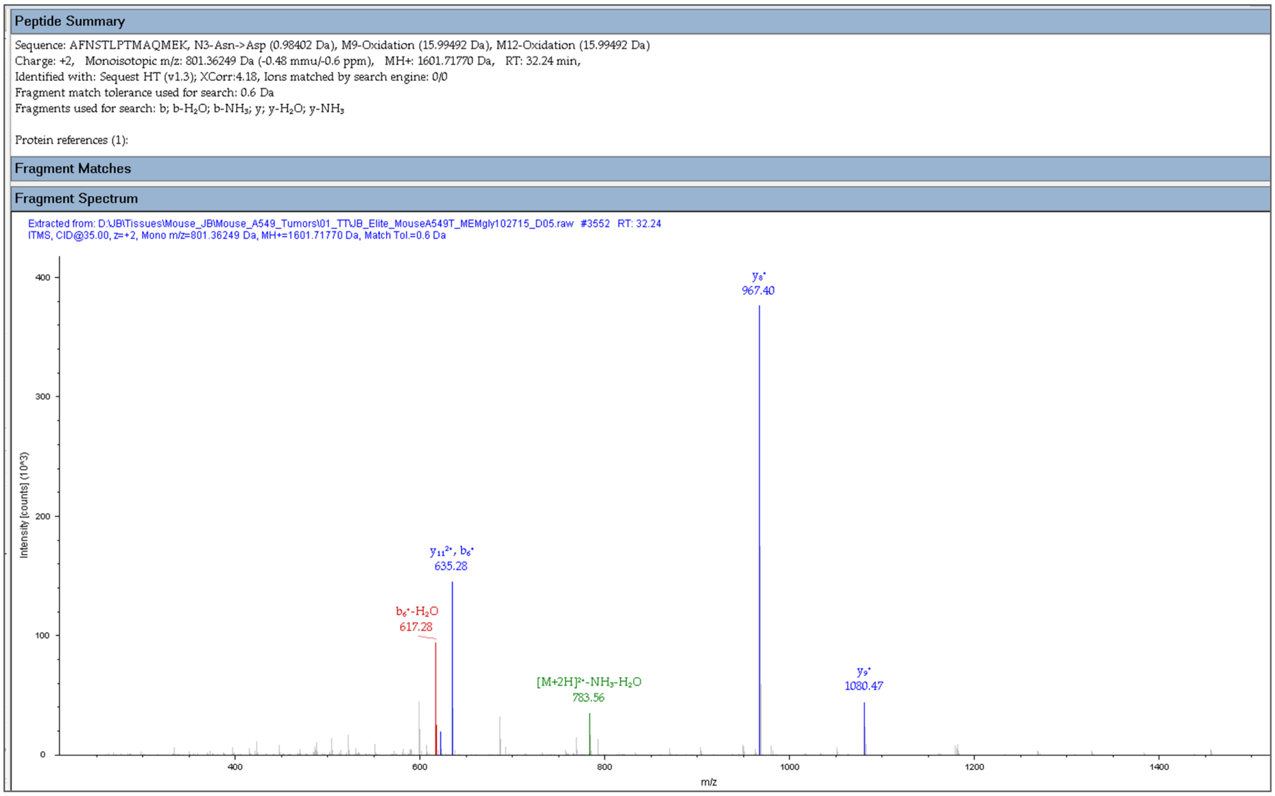


**Supplementary Figure 17: MS$^2$ spectrum depicting unambiguous identification of the human-derived CD44 ortholog in tumor parenchyma by detection of protein- and species-specific N-glycopeptide.**

P15379 CD44_MOUSE - **CD44 antigen** Mus musculus (Mouse)

**E-value:** 0.0
**Score:** 2582
**Ident.:** 67.2%
**Positives :** 75.0%
**Query Length:** 742
**Match Length:** 778

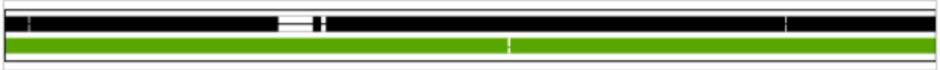

```
P16070 CD44_HUMAN      1    MDKFWWHAAWGLCLVPLSLA--QIDLNITCRFAGVFHVEKNGRYSISRTEAADLCKAFNS     58
                            MDKFWWH AWGLCL+ LSLA  QIDLN+TCR+AGVFHVEKNGRYSISRTEAADLC+AFNS
P15379 CD44_MOUSE      1    MDKFWWHTAWGLCLLQLSLAHQQIDLNVTCRYAGVFHVEKNGRYSISRTEAADLCQAFNS     60

P16070 CD44_HUMAN      59   TLPTMAQMEKALSIGFETCRYGFIEGHVVIPRIHPNSICAANNTGVYIL-TSNTSQYDTY     117
                            TLPTM QM+ ALS GFETCRYGFIEG+VVIPRIHPN+ICAAN+TGVYIL TSNTS YDTY
P15379 CD44_MOUSE      61   TLPTMDQMKLALSKGFETCRYGFIEGNVVIPRIHPNAICAANHTGVYILVTSNTSHYDTY     120

P16070 CD44_HUMAN      118  CFNASAPPEEDCTSVTDLPNAFDGPITITIVNRDGTRYVQKGEYRTNPEDIYPSNPTDDD     177
                            CFNASAPPEEDCTSVTDLPN+FDGP+TITIVNRDGTRY +KGEYRT+ EDI  SN  DDD
P15379 CD44_MOUSE      121  CFNASAPPEEDCTSVTDLPNSFDGPVTITIVNRDGTRYSKKGEYRTHQEDIDASNIIDDD     180

P16070 CD44_HUMAN      178  VSSGSSSERSSTSGGYIFYTF-STVHPIPDEDSPWITDSTDRIPATTLMS----------     226
                            VSSGS+ E+S T  GYI +T+  T  P  D+D  +   ST    A+T+ S
P15379 CD44_MOUSE      181  VSSGSTIEKS-TPEGYILHTYLPTEQPTGDQDDSFFIRSTLATIASTVHSKSHAAAQKQN     239

P16070 CD44_HUMAN      227  --------------------TSATAT-----ETATKRQETWDWFSWLFLPSESKNHLHTT     261
                                                TSAT       ET  KRQE  +WFSWLF PSESK+HLHTT
P15379 CD44_MOUSE      240  NWIWSWFGNSQSTTQTQEPTTSATTALMTTPETPPKRQEAQNWFSWLFQPSESKSHLHTT     299

P16070 CD44_HUMAN      262  TQMAGTSSNTISAGWEPNEENEDERDRHLSFSGSGIDDDEDFISSTISTTPRAFDHTKQN     321
                            T+M GT SNT   GWEPNEENEDE D + SFSGSGIDDDEDFISSTI+TTPR    T+ N
P15379 CD44_MOUSE      300  TKMPGTESNTNPTGWEPNEENEDETDTYPSFSGSGIDDDEDFISSTIATTPRVSARTEDN     359
```

**Supplementary Figure 18: BLAST analysis confirming protein and species specificity of identified N-glycopeptides from human-derived CD44.** Underlined in red are protein and species-specific peptides identified solely in tumor parenchyma.

**Peptide Summary**

Sequence: VGNVTAKDPEGLDISYSLR, N3-Asn->Asp (0.98402 Da)
Charge: +3, Monoisotopic m/z: 679.01581 Da (-0.48 mmu/-0.71 ppm), MH+: 2035.03287 Da, RT: 47.57 min,
Identified with: Sequest HT (v1.3); XCorr:5.52, Ions matched by search engine: 0/0
Fragment match tolerance used for search: 0.6 Da
Fragments used for search: b; b-$H_2O$; b-$NH_3$; y; y-$H_2O$; y-$NH_3$

Protein references (1):

**Fragment Matches**

**Fragment Spectrum**

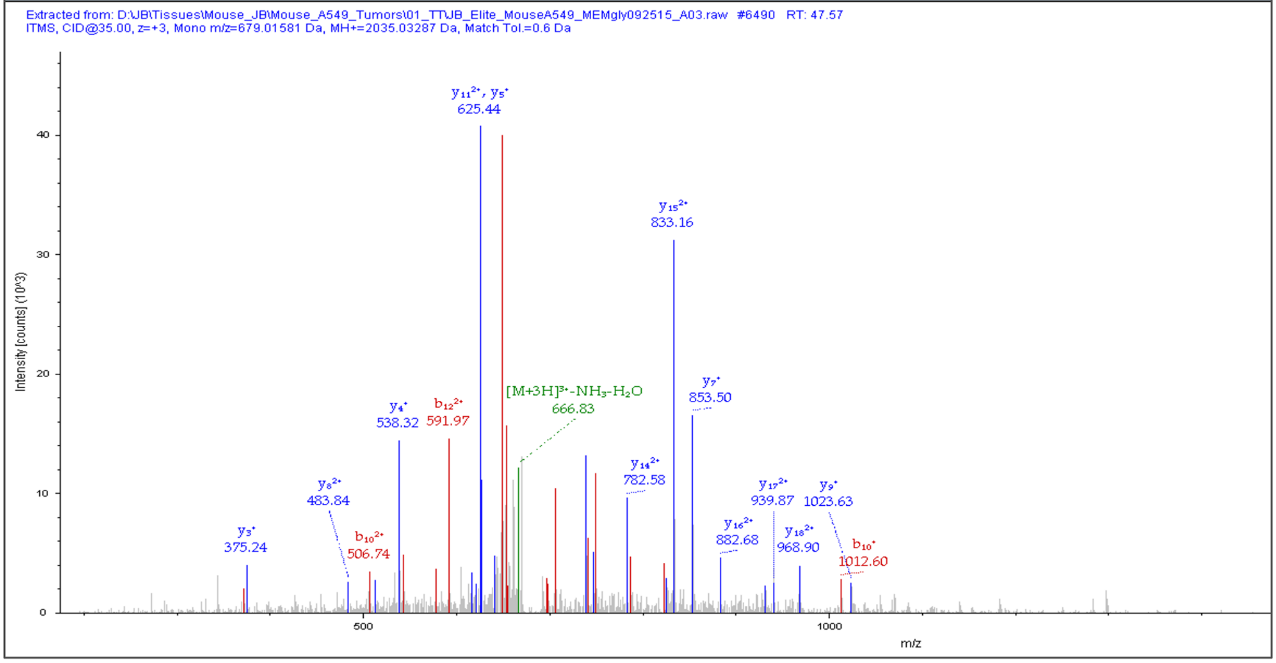


**Supplementary Figure 19: MS$^2$ spectrum depicting unambiguous identification of the human-derived CDH17 ortholog in tumor parenchyma by detection of protein- and species-specific N-glycopeptide.**

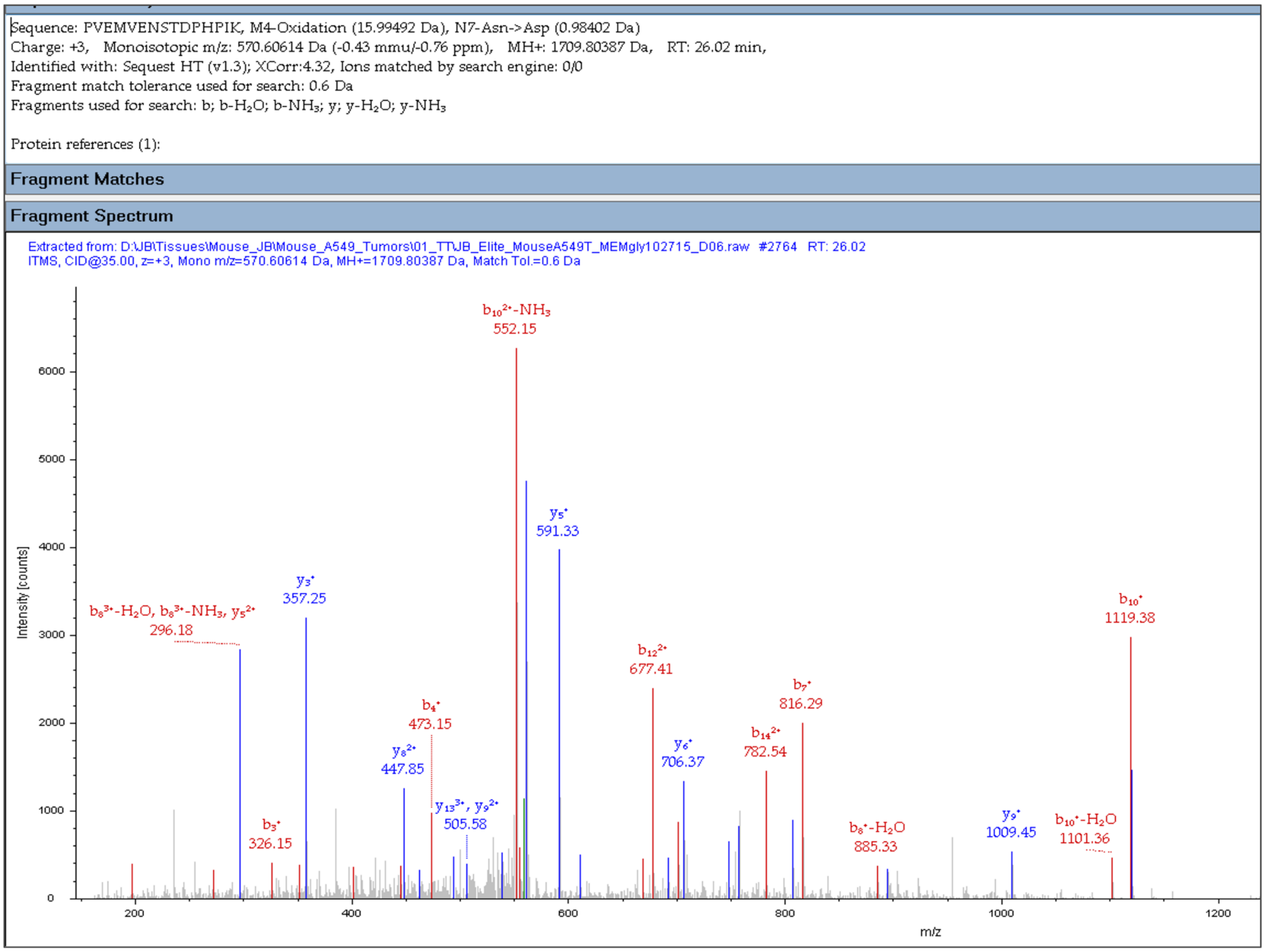


**Supplementary Figure 20: MS$^2$ spectrum depicting unambiguous identification of the human-derived CDH17 ortholog in tumor parenchyma by detection of protein- and species-specific N-glycopeptide.**

Q9R100 CAD17_MOUSE - **Cadherin-17** Mus musculus (Mouse)

E-value: 0.0
Score: 3443
Ident.: 79.1%
Positives : 88.2%
Query Length: 832
Match Length: 827

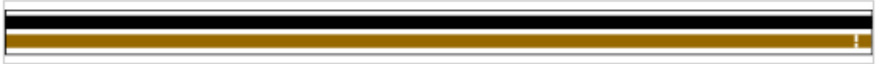

```
Q12864 CAD17_HUMAN    2    ILQAHLHSLCLLMLYLATGYGQEGKFSGPLKPMTFSIYEGQEPSQIIFQFKANPPAVTFE   61
                           ++ A LH LCLL LYL  GYG+EGKFSGPLKPMTFSI+EGQEPSQ+IFQFK NPPAVTFE
Q9R100 CAD17_MOUSE    1    MVSAQLHFLCLLTLYLTCGYGEEGKFSGPLKPMTFSIFEGQEPSQVIFQFKTNPPAVTFE   60

Q12864 CAD17_HUMAN   62    LTGETDNIFVIEREGLLYYNRALDRETRSTHNLQVAALDANGIIVEGPVPITIKVKDIND  121
                           LTGETD IF IE++GLLY+ RALDRETR+ H+LQ+AALD++G IV+GPVPITI+VKDIND
Q9R100 CAD17_MOUSE   61    LTGETDGIFKIEKDGLLYHTRALDRETRAVHHLQLAALDSHGAIVDGPVPITIEVKDIND  120

Q12864 CAD17_HUMAN  122    NRPTFLQSKYEGSVRQNSRPGKPFLYVNATDLDDPATPNGQLYYQIVIQLPMINNVMYFQ  181
                           NRPTFLQSKYEGSVRQNSRPGKPF+YVNATDLDDPATPNGQL+YQIVIQLP IN+VMYFQ
Q9R100 CAD17_MOUSE  121    NRPTFLQSKYEGSVRQNSRPGKPFMYVNATDLDDPATPNGQLFYQIVIQLPQINDVMYFQ  180

Q12864 CAD17_HUMAN  182    INNKTGAISLTREGSQELNPAKNPSYNLVISVKDMGGQSENSFSDTTSVDIIVTENIWKA  241
                           I++KTGAISLT EGSQEL+P KNPSYNLV+SVKDMGGQSENSFSDTT VDI + ENIWKA
Q9R100 CAD17_MOUSE  181    IDSKTGAISLTPEGSQELDPVKNPSYNLVVSVKDMGGQSENSFSDTTYVDISIRENIWKA  240

Q12864 CAD17_HUMAN  242    PKPVEMVENSTDPHPIKITQVRWNDPGAQYSLVDKEKLPRFPFSIDQEGDIYVTQPLDRE  301
                           P+PVE+ ENSTDPHPIKITQV+WNDPGAQYSLV+KEKL  FPFSIDQEG+IYVTQ LDRE
Q9R100 CAD17_MOUSE  241    PEPVEIRENSTDPHPIKITQVQWNDPGAQYSLVNKEKLSPFPFSIDQEGNIYVTQALDRE  300

Q12864 CAD17_HUMAN  302    EKDAYVFYAVAKDEYGKPLSYPLEIHVKVKDINDNPPTCPSPVTVFEVQENERLGNSIGT  361
                           EK+++VF+A AKDE GKPL+YPLEI+VKV DINDNPPTC SPVTVFEVQENE LGNSIG
Q9R100 CAD17_MOUSE  301    EKNSHVFFATAKDENGKPLAYPLEIYVKVIDINDNPPTCLSPVTVFEVQENEPLGNSIGI  360

Q12864 CAD17_HUMAN  362    LTAHDRDEENTANSFLNYRIVEQTPKLPMDGLFLIQTYAGMLQLAKQSLKKQDTPQYNLT  421
                             AHD DE N  NS L Y++V+QTPK+P DGLFLI  Y G +QL+KQSLKKQD+PQYNL+
Q9R100 CAD17_MOUSE  361    FEAHDMDEANNINSILKYKLVDQTPKVPSDGLFLIGEYEGKVQLSKQSLKKQDSPQYNLS  420

Q12864 CAD17_HUMAN  422    IEVSDKDFKTLCFVQINVIDINDQIPIFEKSDYGNLTLAEDTNIGSTILTIQATDADEPF  481
                           IEVSD DFKTLC++Q+NVIDINDQIPIFE S+YG+ TL+EDT IGSTIL IQATDADEPF
Q9R100 CAD17_MOUSE  421    IEVSDVDFKTLCYIQVNVIDINDQIPIFETSNYGSKTLSEDTAIGSTILIIQATDADEPF  480

Q12864 CAD17_HUMAN  482    TGSSKILYHIIKGDSEGRLGVDTDPHTNTGYVIIKKPLDFETAAVSNIVFKAENPEPLVF  541
                           TGSSKILY I++GD+EGRL V TDP TN GYV IKKPLDFET  VS+IVF+AENPEPLV
Q9R100 CAD17_MOUSE  481    TGSSKILYKIVQGDTEGRLEVVTDPTTNAGYVKIKKPLDFETQPVSSIVFQAENPEPLVK  540

Q12864 CAD17_HUMAN  542    GVKYNASSFAKFTLIVTDVNEAPQFSQHVFQAKVSEDVAIGTKVGNVTAKDPEGLDISYS  601
                           G++YNASSFA F LIVTDVNE P F Q +FQA VSED A+G++VGNVTA+DPEGL +SYS
Q9R100 CAD17_MOUSE  541    GIEYNASSFASFELIVTDVNEVPVFPQRIFQANVSEDAAVGSRVGNVTARDPEGLTVSYS  600

Q12864 CAD17_HUMAN  602    LRGDTRGWLKIDHVTGEIFSVAPLDREAGSPYRVQVVATEVGGSSLSSVSEFHLILMDVN  661
                           L+G+ RGWLKID VTGEIFS APLDRE  S YRVQVVATEVGGSSLSS ++FHL+L DVN
Q9R100 CAD17 MOUSE  601    LKGNMRGWLKIDSVTGEIFSAAPLDRETESVYRVQVVATEVGGSSLSSTADFHLVLTDVN  660
```

**Supplementary Figure 21: BLAST analysis confirming protein and species specificity of identified N-glycopeptides from parenchymal human-derived CDH17.** Underlined in red are protein and species-specific peptides identified in tumor parenchyma.

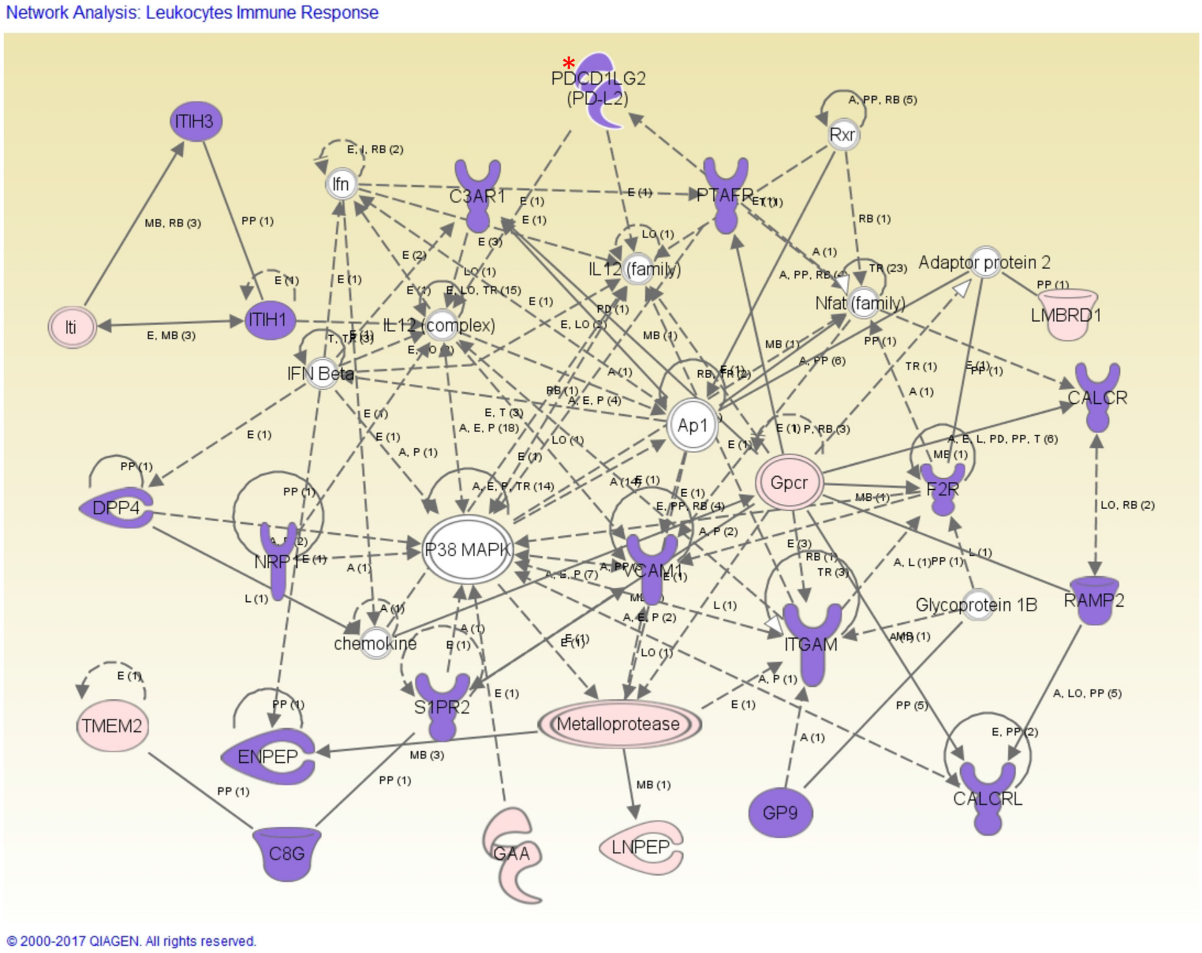


**Supplementary Figure 22: IPA® analysis reveals leukocyte immune response as one of top N-glycosylated protein networks activated in tumor stroma.** Molecule selected for orthogonal cross-validation (i.e., PD-L2) is marked with red asterisk.

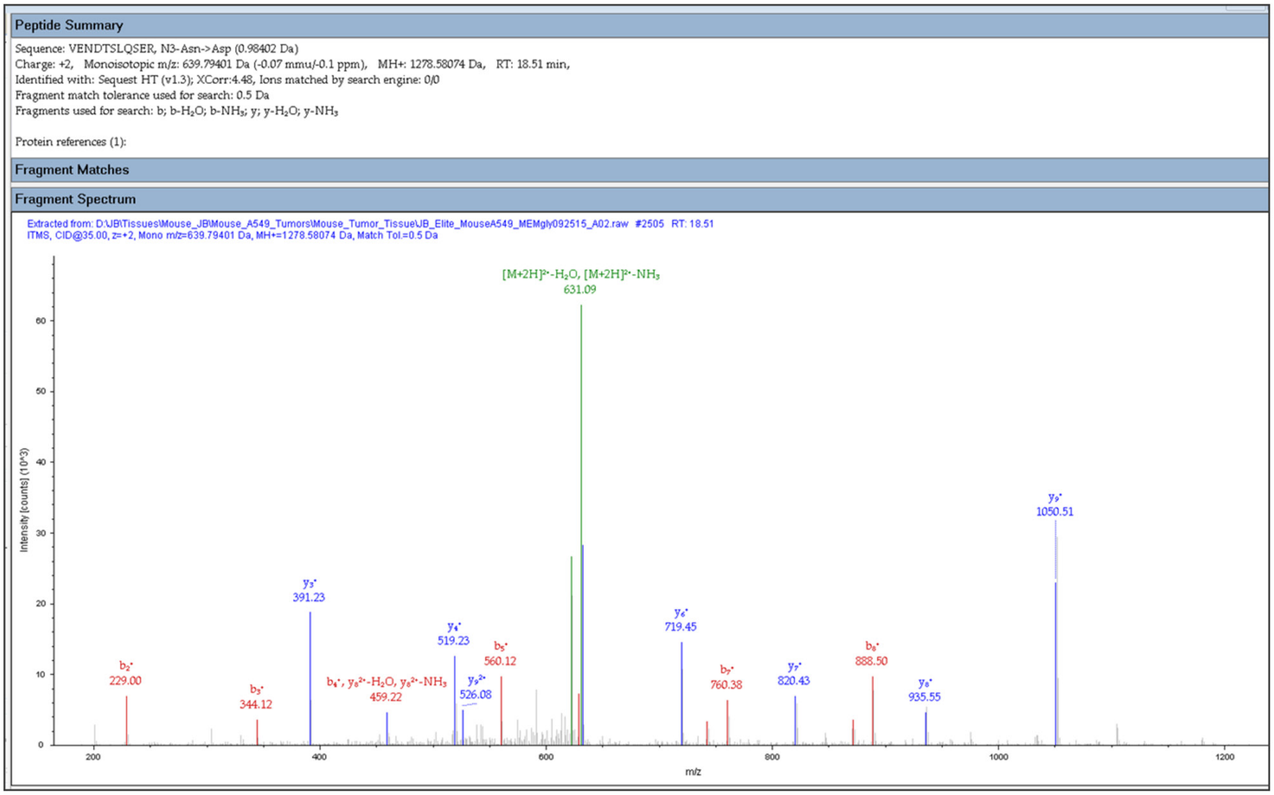


**Supplementary Figure 23: MS² spectrum depicting unambiguous identification of the mouse-derived PD-L2 ortholog in tumor stroma by detection of protein- and species-specific N-glycopeptide.**

Q9BQ51 PD1L2_HUMAN - **Programmed cell death 1 ligand 2** Homo sapiens (Human)

E-value: 2.2e-117

Score: 895

Ident.: 69.6%

Positives : 80.6%

Query Length: 247

Match Length: 273

**Identification of the mouse Programmed cell death 1 ligand 2 (Pdcd1lg2) only in tumor stroma (i.e. highlighted in blue) by 36 PSMs from species and protein specific glyco-peptide**

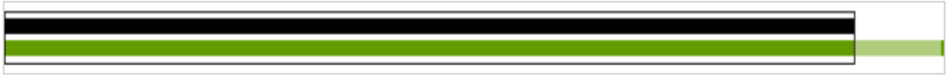

```
Q9WUL5 PD1L2_MOUSE     1     MLLLLPILNLSLQLHPVAALFTVTAPKEVYTVDVGSSVSLECDFDRRECTELEGIRASLQ     60
                             M+ LL +L+L LQLH +AALFTVT PKE+Y ++ GS+V+LEC+FD      L  I ASLQ
Q9BQ51 PD1L2_HUMAN     1     MIFLLLMLSLELQLHQIAALFTVTVPKELYIIEHGSNVTLECNFDTGSHVNLGAITASLQ     60

Q9WUL5 PD1L2_MOUSE     61    KVENDTSLQSERATLLEEQLPLGKALFHIPSVQVRDSGQYRCLVICGAAWDYKYLTVKVK    120
                             KVENDTS   ERATLLEEQLPLGKA FHIP VQVRD GQY+C++I G AWDYKYLT+KVK
Q9BQ51 PD1L2_HUMAN     61    KVENDTSPHRERATLLEEQLPLGKASFHIPQVQVRDEGQYQCIIIYGVAWDYKYLTLKVK    120

Q9WUL5 PD1L2_MOUSE     121   ASYMRIDTRILEVPGTGEVQLTCQARGYPLAEVSWQNVSVPANTSHIRTPEGLYQVTSVL    180
                             ASY +I+T IL+VP T EV+LTCQA GYPLAEVSW NVSVPANTSH RTPEGLYQVTSVL
Q9BQ51 PD1L2_HUMAN     121   ASYRKINTHILKVPETDEVELTCQATGYPLAEVSWPNVSVPANTSHSRTPEGLYQVTSVL    180

Q9WUL5 PD1L2_MOUSE     181   RLKPQPSRNFSCMFWNAHMKELTSAIIDPLSRMEPKVPRTWPLHVFIPACTIALIFLAIV    240
                             RLKP P RNFSC+FWN H++ELT A ID  S+MEP+   TW LH+FIP C IA IF+A V
Q9BQ51 PD1L2_HUMAN     181   RLKPPPGRNFSCVFWNTHVRELTLASIDLQSQMEPRTHPTWLLHIFIPFCIIAFIFIATV    240

Q9WUL5 PD1L2_MOUSE     241   IIQRKRI                                                         247
                             I  RK++
Q9BQ51 PD1L2_HUMAN     241   IALRKQL                                                         247
```

**Supplementary Figure 24: BLAST analysis confirming protein and species specificity of identified N-glycopeptides from stromal mouse-derived PD-L2, Pdcd1lg2 gene product.** Underlined in blue is protein and species-specific peptides identified solely in tumor stroma.

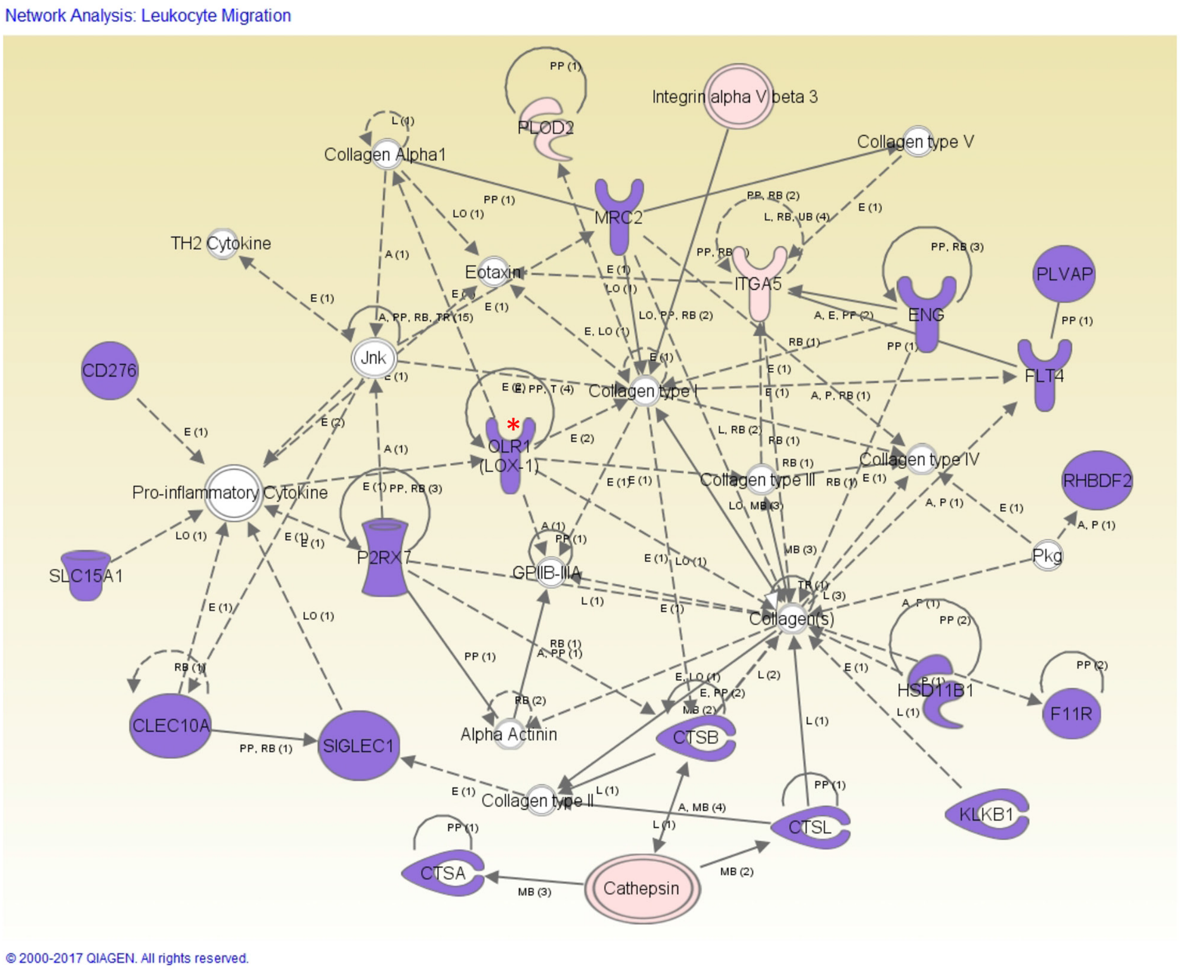


**Supplementary Figure 25: IPA® analysis reveals leukocyte migration as one of top N-glycosylated protein networks activated in tumor stroma.** Molecule selected for orthogonal cross-validation (i.e., LOX-1) is marked with red asterisk.

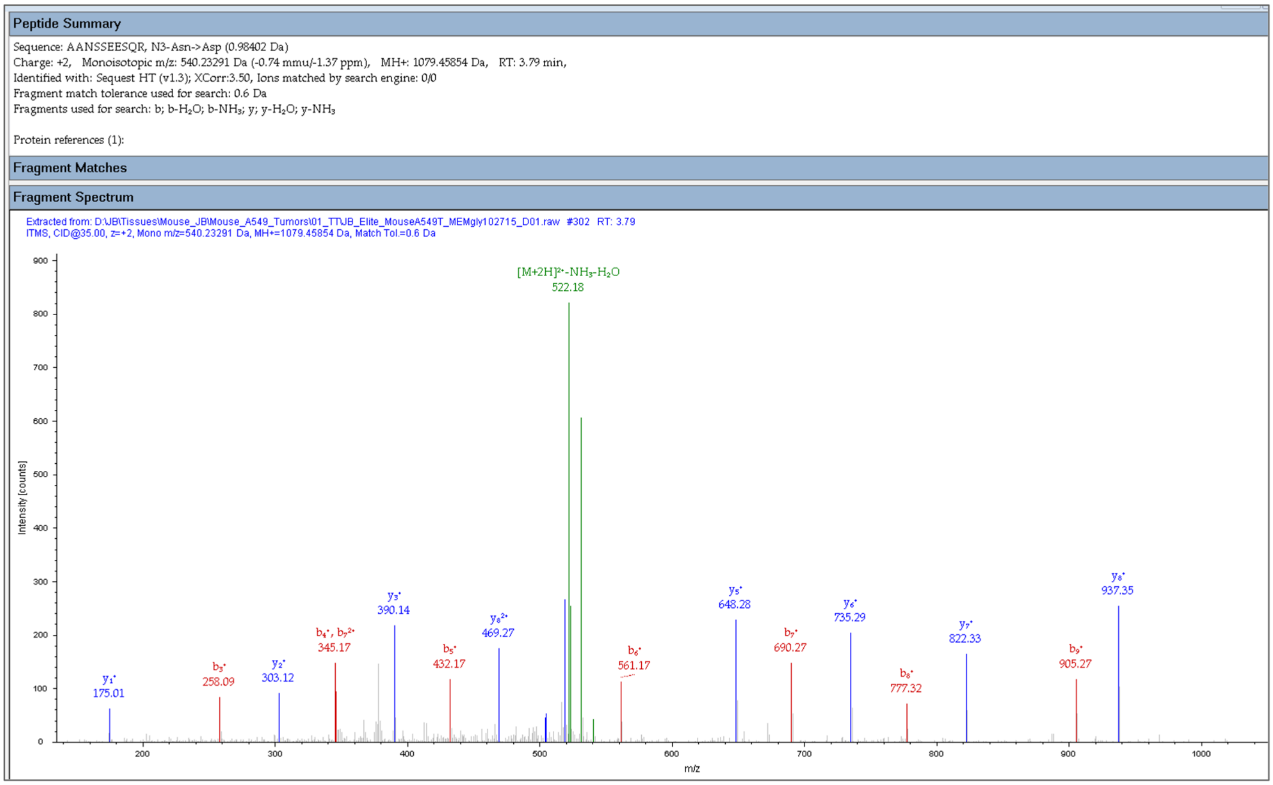


**Supplementary Figure 26: MS$^2$ spectrum depicting unambiguous identification of the mouse-derived LOX-1 ortholog in tumor stroma by detection of protein- and species-specific N-glycopeptide.**

P78380 OLR1_HUMAN - **Oxidized low-density lipoprotein receptor 1** Homo sapiens (Human)

**E-value:** 2.2e-87
**Score:** 707
**Ident.:** 44.9%
**Positives :** 54.6%
**Query Length:** 363
**Match Length:** 273

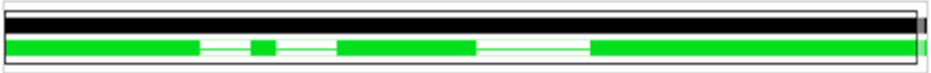

```
Q9EQ09 OLR1_MOUSE   1     MTFDD-KMKPANDEPDQKSCGKKPKGLHLLSSPWWFPAAMTLVILCLVLSVTLIVQWTQL   59
                          MTFDD K++   D+PD+KS GKK KGL  L SPWW  AA TL +LCL L VT++V   QL
P78380 OLR1_HUMAN   1     MTFDDLKIQTVKDQPDEKSNGKKAKGLQFLYSPWWCLAAATLGVLCLGLVVTIMVLGMQL   60

Q9EQ09 OLR1_MOUSE   60    RQVSDLLKQYQANLTQQDRILEGQMLAQQKAENTSQESKKELKGKIDTLTQKLNEKSKEQ   119
                           QVSDLL Q QANLT Q                     KK+L+G+I
P78380 OLR1_HUMAN   61    SQVSDLLTQEQANLTHQ---------------------KKKLEGQIS-------------   86

Q9EQ09 OLR1_MOUSE   120   EELLQKNQNLQEALQRAANSSEESQRELKGKIDTITRKLDEKSKEQEELLQMIQNLQEAL   179
                                      A Q+A  +S+ES+ ELK  I+T+ RKL+EKSKEQ EL     NLQE L
P78380 OLR1_HUMAN   87    ------------ARQQAEEASQESENELKEMIETLARKLNEKSKEQMELHHQNLNLQETL   134

Q9EQ09 OLR1_MOUSE   180   QRAANSSEESQRELKGKIDTLTLKLNEKSKEQEELLQKNQNLQEALQRAANFSGPCPQDW   239
                          +R AN                                               S PCPQDW
P78380 OLR1_HUMAN   135   KRVANC----------------------------------------------SAPCPQDW   148

Q9EQ09 OLR1_MOUSE   240   LWHKENCYLF-HGPFSWEKNRQTCQSLGGQLLQINGADDLTFILQAISHTTSPFWIGLHR   298
                          +WH ENCYLF  G F+WEK+++ C SL  +LL+IN   DL FI QAIS+++ PFW+GL R
P78380 OLR1_HUMAN   149   IWHGENCYLFSSGSFNWEKSQEKCLSLDAKLLKINSTADLDFIQQAISYSSFPFWMGLSR   208

Q9EQ09 OLR1_MOUSE   299   KKPGQPWLWENGTPLNFQFFKTRGVSLQLYSSGNCAYLQDGAVFAENCILIAFSICQKKT   358
                          + P  PWLWE+G+PL    F+ RG   Q Y SG CAY+Q GAV+AENCIL AFSICQKK
P78380 OLR1_HUMAN   209   RNPSYPWLWEDGSPLMPHLFRVRGAVSQTYPSGTCAYIQRGAVYAENCILAAFSICQKKA   268
```

**Supplementary Figure 27: BLAST analysis confirming protein and species specificity of identified N-glycopeptides from stromal mouse-derived LOX-1, Olr1 gene product.** Underlined in blue is protein and species-specific peptides identified solely in tumor stroma.

Network Analysis: Humoral Immune Response

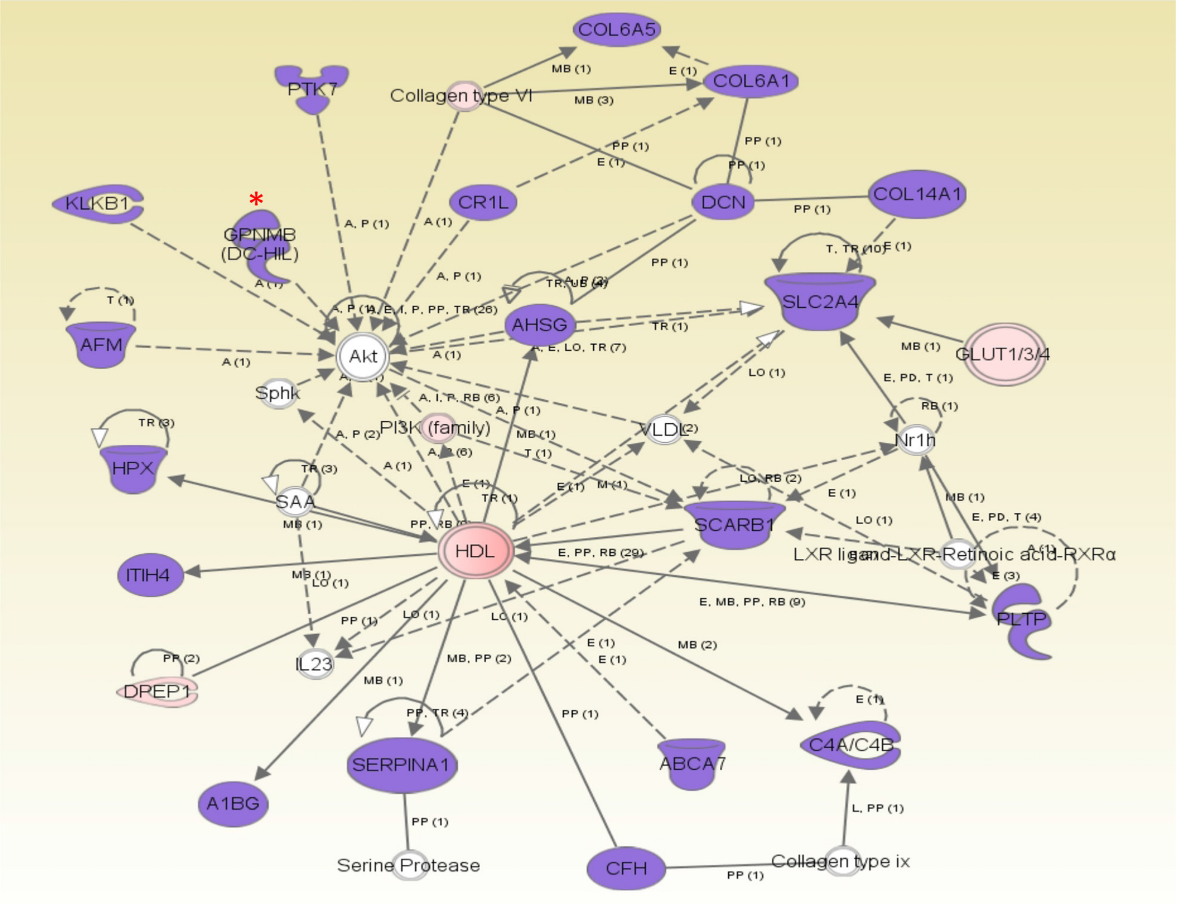


© 2000-2017 QIAGEN. All rights reserved.

**Supplementary Figure 28: IPA® analysis reveals humoral immune response as one of top N-glycosylated protein networks activated in tumor stroma.** Molecule selected for orthogonal cross-validation (i.e., DC-HIL) is marked with red asterisk.

**Peptide Summary**

Sequence: VSINTVNLTAGPQVMEVTVFR, N7-Asn->Asp (0.98402 Da), M15-Oxidation (15.99492 Da)
Charge: +3,   Monoisotopic m/z: 764.73547 Da (+0.45 mmu/+0.59 ppm),   MH+: 2292.19187 Da,   RT: 58.82 min,
Identified with: Sequest HT (v1.3); XCorr:6.11, Ions matched by search engine: 0/0
Fragment match tolerance used for search: 0.5 Da
Fragments used for search: b; b-$H_2O$; b-$NH_3$; y; y-$H_2O$; y-$NH_3$

Protein references (1):

**Fragment Matches**

**Fragment Spectrum**

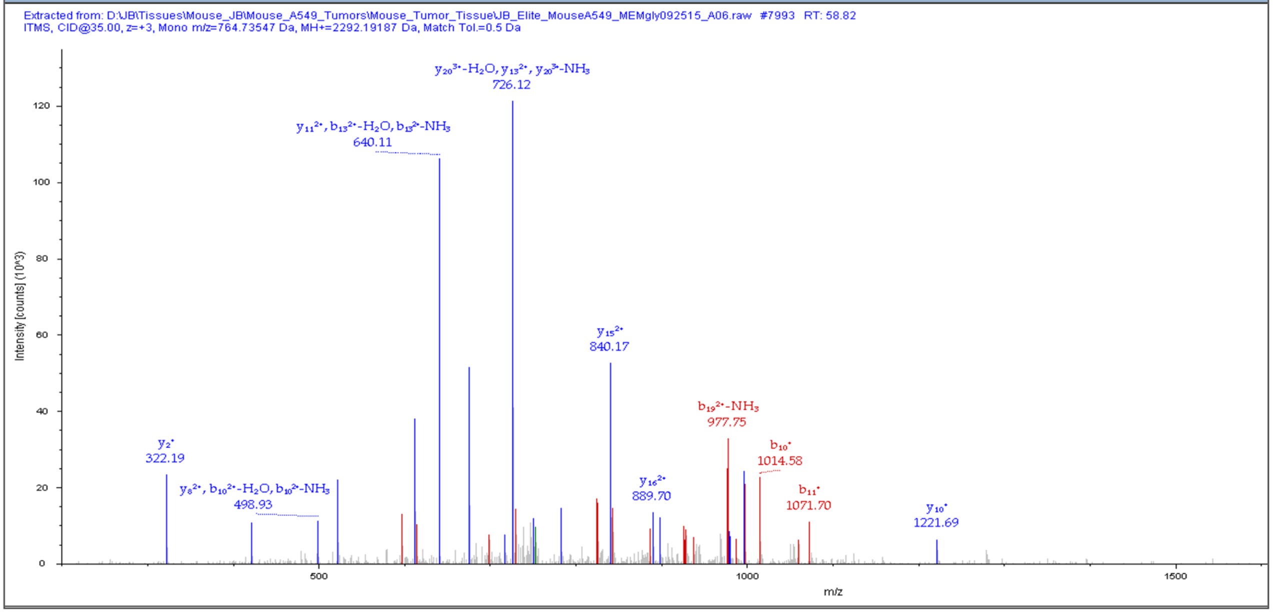


**Supplementary Figure 29: MS$^2$ spectrum depicting unambiguous identification of the mouse-derived DC-HIL ortholog (i.e., Gpnmb gene product) in tumor stroma by detection of protein- and species-specific N-glycopeptide.**

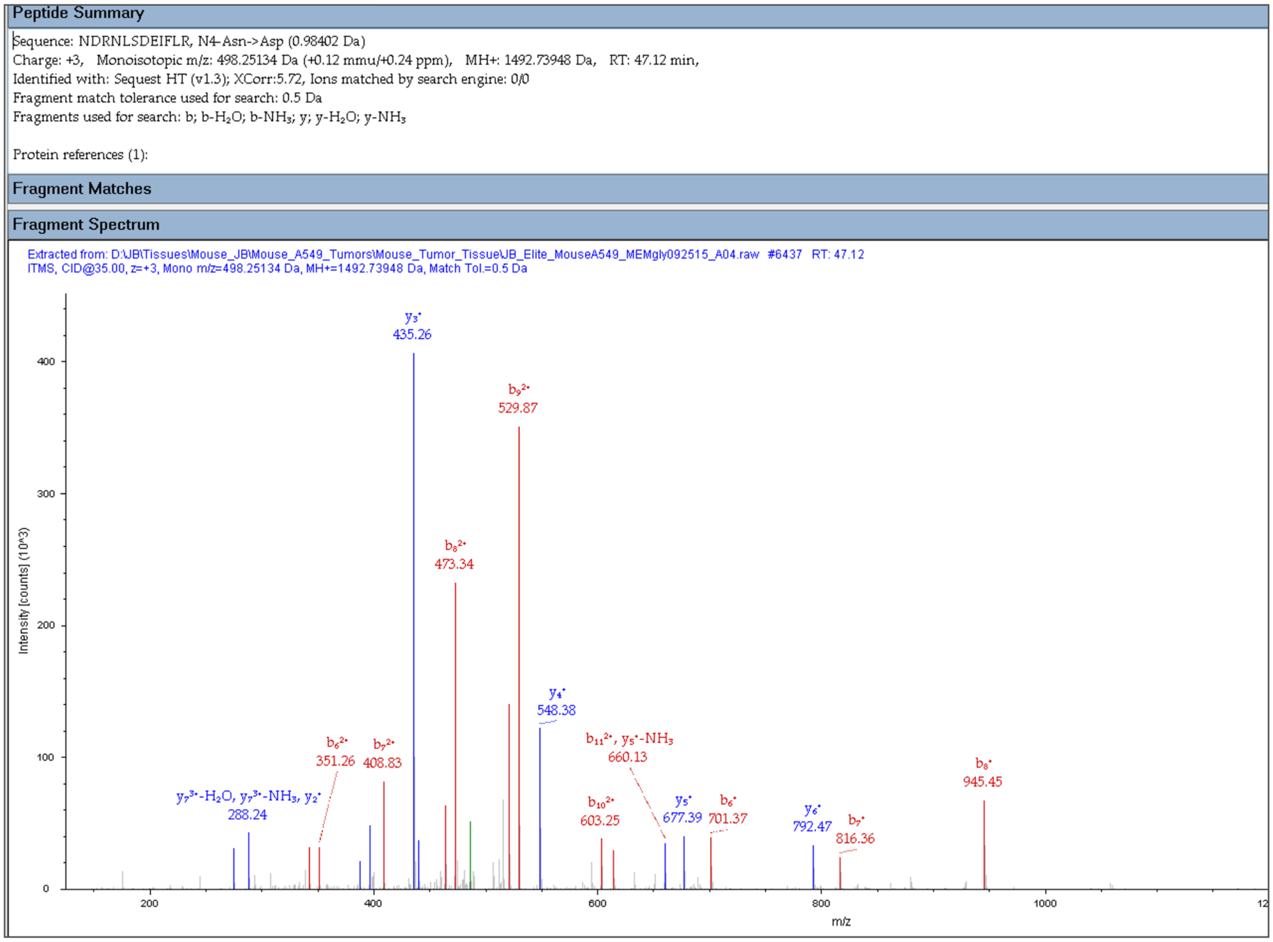


**Supplementary Figure 30: MS[2] spectrum depicting unambiguous identification of the mouse-derived DC-HIL ortholog (i.e., Gpnmb gene product) in tumor stroma by detection of protein- and species-specific N-glycopeptide.**

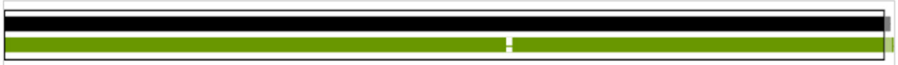

```
Q99P91 GPNMB_MOUSE     1    MESLCGVLGFLLLAAGLPLQAAKRFRDVLGHEQYPDHMREHNQLRGWSSDENEWDEHLYP    60
                           ME L   LGFLLLAA LPL AAKRF DVLG+E+   +MREHNQL GWSSDEN+W+E LYP
Q14956 GPNMB_HUMAN     1    MECLYYFLGFLLLAARLPLDAAKRFHDVLGNERPSAYMREHNQLNGWSSDENDWNEKLYP    60

Q99P91 GPNMB_MOUSE     61   VWRRGDGRWKDSWEGGRVQAVLTSDSPALVGSNITFVVNLVFPRCQKEDANGNIVYEKNC    120
                           VW+RGD RWK+SW+GGRVQAVLTSDSPALVGSNITF VNL+FPRCQKEDANGNIVYEKNC
Q14956 GPNMB_HUMAN     61   VWKRGDMRWKNSWKGGRVQAVLTSDSPALVGSNITFAVNLIFPRCQKEDANGNIVYEKNC    120

Q99P91 GPNMB_MOUSE     121  RNDLGLTSDLHVYNWTAGADDGDWEDGTSRSQHLRFPDRRPFPRPHGWKKWSFVYVFHTL    180
                           RN+ GL++D +VYNWTA ++D D E+GT +S H  FPD +PFP   GW++W+F+YVFHTL
Q14956 GPNMB_HUMAN     121  RNEAGLSADPYVYNWTAWSEDSDGENGTGQSHHNVFPDGKPFPHHPGWRRWNFIYVFHTL    180

Q99P91 GPNMB_MOUSE     181  GQYFQKLGRCSARVSINTVNLTAGPQVMEVTVFRRYGRAYIPISKVKDVYVITDQIPVFV    240
                           GQYFQKLGRCS RVS+NT N+T GPQ+MEVTV+RR+GRAY+PI++VKDVYV+TDQIPVFV
Q14956 GPNMB_HUMAN     181  GQYFQKLGRCSVRVSVNTANVTLGPQLMEVTVYRRHGRAYVPIAQVKDVYVVTDQIPVFV    240

Q99P91 GPNMB_MOUSE     241  TMSQKNDRNLSDEIFLRDLPIVFDVLIHDPSHFLNDSAISYKWNFGDNTGLFVSNNHTLN    300
                           TM QKNDRN SDE FL+DLPI+FDVLIHDPSHFLN S I+YKW+FGDNTGLFVS NHT+N
Q14956 GPNMB_HUMAN     241  TMFQKNDRNSSDETFLKDLPIMFDVLIHDPSHFLNYSTINYKWSFGDNTGLFVSTNHTVN    300

Q99P91 GPNMB_MOUSE     301  HTYVLNGTFNLNLTVQTAVPGPCPPPSPSTPPPPSTPPSPPPSPLPTL-STPSPSLMPTG    359
                           HTYVLNGTF+LNLTV+ A PGPCPP     PPPP  P  P PS   TL S  S +  P G
Q14956 GPNMB_HUMAN     301  HTYVLNGTFSLNLTVKAAAPGPCPP-----PPPPPRPSKPTPSLATTLKSYDSNTPGPAG    355

Q99P91 GPNMB_MOUSE     360  YKSMELSDISNENCRINRYGYFRATITIVEGILEVSIMQIADVPMPTPQPANSLMDFTVT    419
                              +ELS I +ENC+INRYG+F+ATITIVEGILEV+I+Q+ DV MP P P +SL+DF VT
Q14956 GPNMB_HUMAN     356  DNPLELSRIPDENCQINRYGHFQATITIVEGILEVNIIQMTDVLMPVPWPESSLIDFVVT    415

Q99P91 GPNMB_MOUSE     420  CKGATPMEACTIISDPTCQIAQNRVCSPVAVDGLCLLSVRRAFNGSGTYCVNFTLGDDAS    479
                           C+G+ P E CTIISDPTC+I QN VCSPV VD +CLL+VRR FNGSGTYCVN TLGDD S
Q14956 GPNMB_HUMAN     416  CQGSIPTEVCTIISDPTCEITQNTVCSPVDVDEMCLLTVRRTFNGSGTYCVNLTLGDDTS    475

Q99P91 GPNMB_MOUSE     480  LALTSTLISIPGKDPDSPLRAVNGVLISIGCLAVLVTMVTILLYKKHKAYKPIGNCPRNT    539
                           LALTSTLIS+P +DP SPLR  N  LIS+GCLA+ VT++++L+YKKHK Y PI N P N
Q14956 GPNMB_HUMAN     476  LALTSTLISVPDRDPASPLRMANSALISVGCLAIFVTVISLLVYKKHKEYNPIENSPGNV    535

Q99P91 GPNMB_MOUSE     540  VKGKGLSVLLSHAKAPFFRGDQEKDPLLQDK    570
                           V+ KGLSV L+ AKA FF G+QEKDPLL+++
Q14956 GPNMB_HUMAN     536  VRSKGLSVFLNRAKAVFFPGNQEKDPLLKNQ    566
```

**Supplementary Figure 31: BLAST analysis confirming protein and species specificity of identified N-glycopeptides from stromal mouse-derived DC-HIL, Gpnmb gene product.** Underlined in blue are protein and species-specific peptides identified solely in tumor stroma.

Network Analysis: Movement Disorder

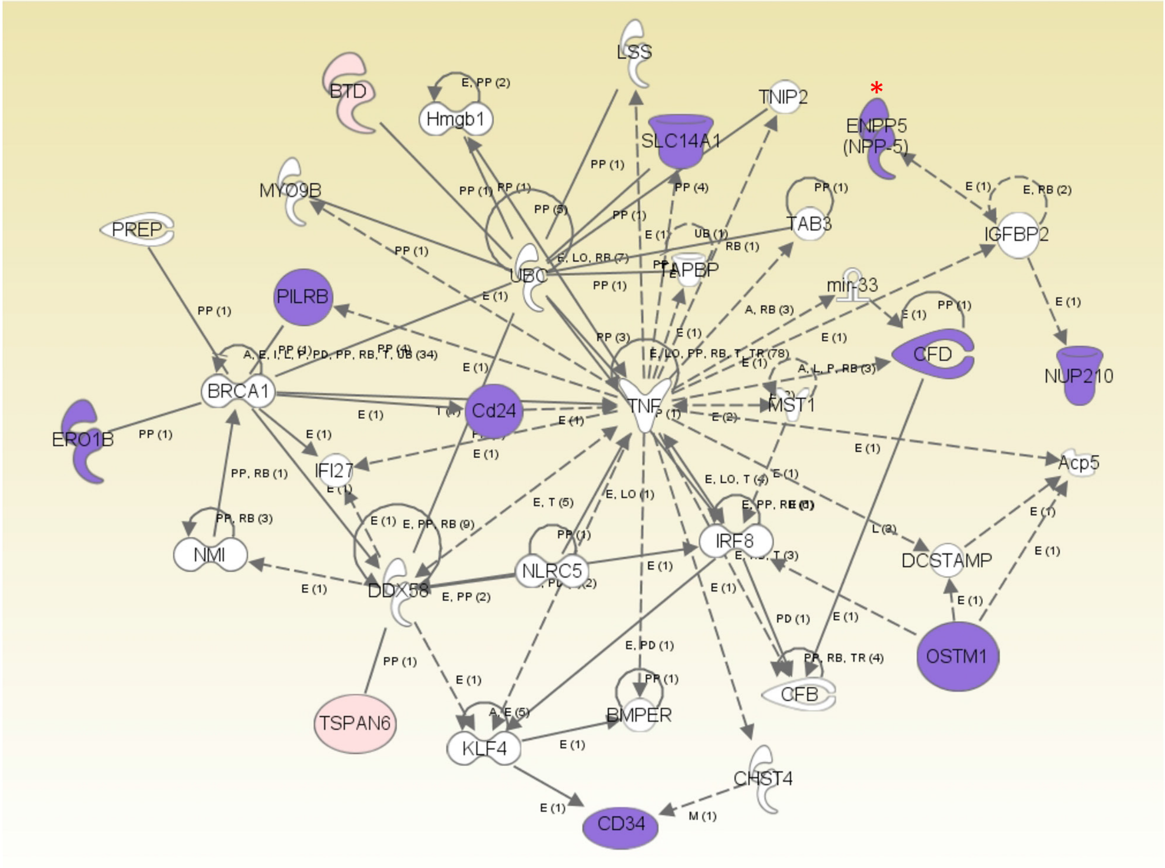


© 2000-2017 QIAGEN. All rights reserved.

**Supplementary Figure 32: IPA® analysis reveals humoral immune response as one of top N-glycosylated protein networks activated in tumor stroma.** Molecule selected for orthogonal cross-validation (i.e., NPP-5) is marked with red asterisk.

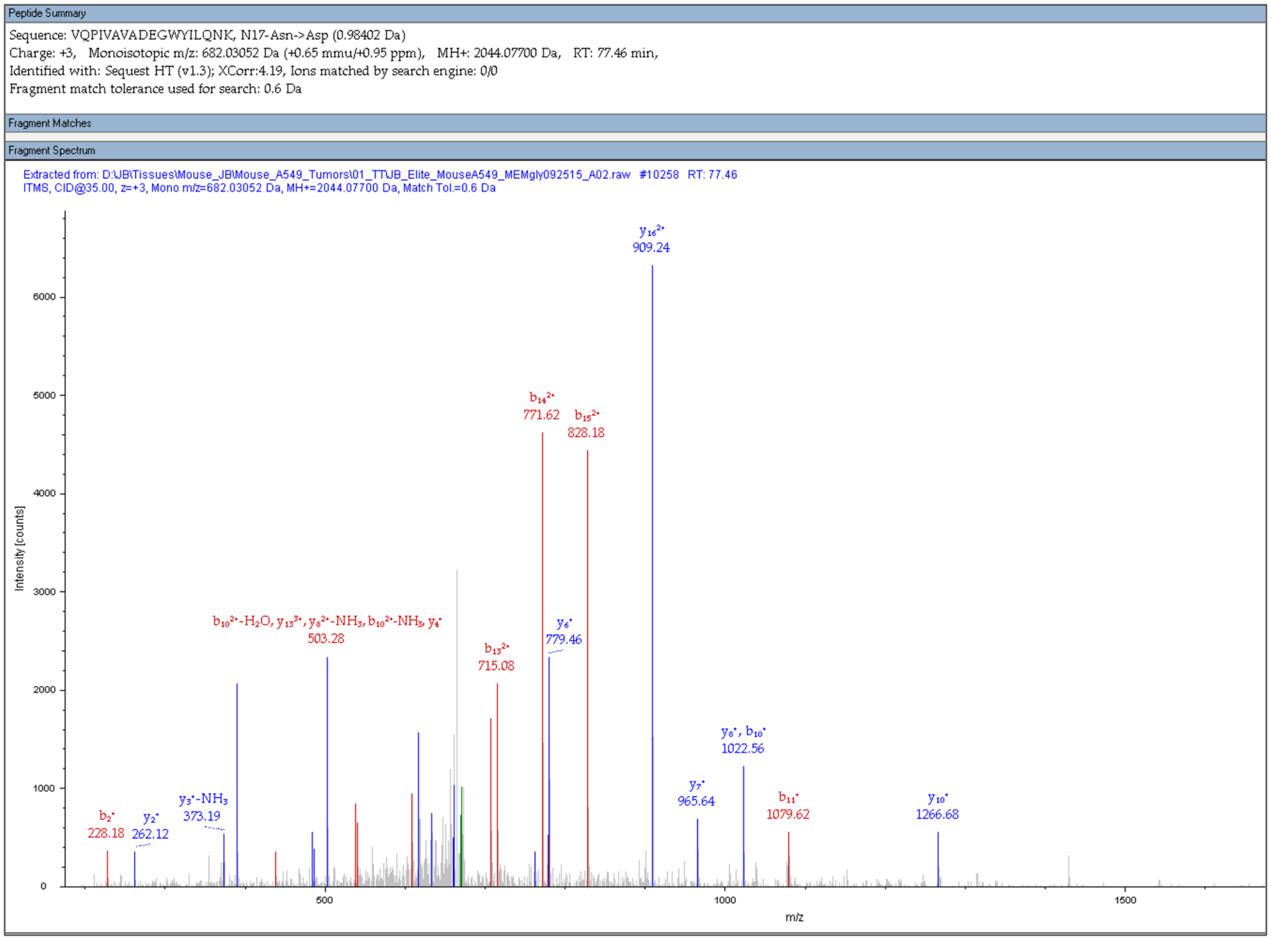


Supplementary Figure 33: MS$^2$ spectrum depicting unambiguous identification of the mouse-derived NPP-5 ortholog (i.e., Enpp5 gene product) in tumor stroma by detection of protein- and species-specific N-glycopeptide.

Q9UJA9 ENPP5_HUMAN - **Ectonucleotide pyrophosphatase/phosphodiesterase family member 5** Homo sapiens (Human)

E-value: 0.0

Score: 2152

Ident.: 81.8%

Positives : 90.6%

Query Length: 477

Match Length: 477

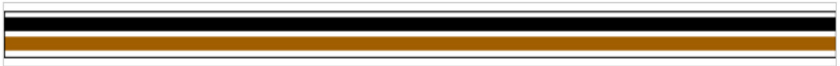

```
Q9EQG7 ENPP5_MOUSE    1    MIPEFLLASCTLATLCHSAPFSLQPEEQKVLVVSFDGFRWDYLYKVPTPHFHYIMKNGVH    60
                           M  +FLL S  LA L  S  FSLQP++QKVL+VSFDGFRWDYLYKVPTPHFHYIMK GVH
Q9UJA9 ENPP5_HUMAN    1    MTSKFLLVSFILAALSLSTTFSLQPDQQKVLLVSFDGFRWDYLYKVPTPHFHYIMKYGVH    60

Q9EQG7 ENPP5_MOUSE    61   VNQVTNVFITKTYPNHYTLVTGLFAENHGIVANDMFDPILNKSFSLEHMDIYDSKFWEEA    120
                           V QVTNVFITKTYPNHYTLVTGLFAENHGIVANDMFDPI NKSFSL+HM+IYDSKFWEEA
Q9UJA9 ENPP5_HUMAN    61   VKQVTNVFITKTYPNHYTLVTGLFAENHGIVANDMFDPIRNKSFSLDHMNIYDSKFWEEA    120

Q9EQG7 ENPP5_MOUSE    121  TPIWITNQRAGHASGAAMWPGADVKIHDSFPTYYLPYNESVSFEDRVAKIIEWFTAKDPI    180
                           TPIWITNQRAGH SGAAMWPG DVKIH  FPT+Y+PYNESVSFEDRVAKIIEWFT+K+PI
Q9UJA9 ENPP5_HUMAN    121  TPIWITNQRAGHTSGAAMWPGTDVKIHKRFPTHYMPYNESVSFEDRVAKIIEWFTSKEPI    180

Q9EQG7 ENPP5_MOUSE    181  NLGFLYWEEPDDTGHDVGPDSPLMGSVISDVDHKLGYLIKMLKRAKLWNNVNLIVTSDHG    240
                           NLG LYWE+PDD GH +GPDSPLMG VISD+D KLGYLI+MLK+AKLWN +NLI+TSDHG
Q9UJA9 ENPP5_HUMAN    181  NLGLLYWEDPDDMGHHLGPDSPLMGPVISDIDKKLGYLIQMLKKAKLWNTLNLIITSDHG    240

Q9EQG7 ENPP5_MOUSE    241  MTQCSKQRVIELDRYLDKEHYTLIDHSPVAAILPKEGKFDEVYDALAGAHPNLTVYKKEE    300
                           MTQCS++R+IELD+YLDK+HYTLID SPVAAILPKEGKFDEVY+AL  AHPNLTVYKKE+
Q9UJA9 ENPP5_HUMAN    241  MTQCSEERLIELDQYLDKDHYTLIDQSPVAAILPKEGKFDEVYEALTHAHPNLTVYKKED    300

Q9EQG7 ENPP5_MOUSE    301  IPERWHYKHNDRVQPIVAVADEGWYILQNKSDDFLLGNHGYDNALAEMHPIFLAHGPAFR    360
                           +PERWHYK+N R+QPI+AVADEGW+ILQNKSDDFLLGNHGYDNALA+MHPIFLAHGPAFR
Q9UJA9 ENPP5_HUMAN    301  VPERWHYKYNSRIQPIIAVADEGWHILQNKSDDFLLGNHGYDNALADMHPIFLAHGPAFR    360

Q9EQG7 ENPP5_MOUSE    361  KNFTKEAMNSTDLYSLLCHLLNLTALPHNGSFWNVQDLLSSATPKPIPYTQSTTLLLGSD    420
                           KNF+KEAMNSTDLY LLCHLLN+TA+PHNGSFWNVQDLL+SA P+ +PYTQST LL GS
Q9UJA9 ENPP5_HUMAN    361  KNFSKEAMNSTDLYPLLCHLLNITAMPHNGSFWNVQDLLNSAMPRVVPYTQSTILLPGSV    420

Q9EQG7 ENPP5_MOUSE    421  KPGEDEQEESYPYYIGVSLGSIIAMVFFVVLIKHLIRSQVHTLQYRQVEVAQPLLQA    477
                           KP E +QE SYPY+IGVSLGSII +VFFV+ IKHLI SQ+  LQ    E+AQPLLQA
Q9UJA9 ENPP5_HUMAN    421  KPAEYDQEGSYPYFIGVSLGSIIVIVFFVIFIKHLIHSQIPALQDMHAEIAQPLLQA    477
```

**Supplementary Figure 34: BLAST analysis confirming protein and species specificity of identified N-glycopeptides from stromal mouse-derived NPP-5, Enpp5 gene product.** Underlined in blue are protein and species-specific peptides identified solely in tumor stroma.

**Supplementary Table 1: (A) N-glycosylated human peptides identified in A549 murine tumor xenograft using TCSG. (B) N-glycosylated mouse peptides identified in A549 murine tumor xenograft using TCSG.**

**See Supplementary File 1**

**Supplementary Table 2: (A) N-glycosylated peptides identified in normal mouse lung tissue using TCSG. (B) N-glycosylated peptides identified in 2D-grown A549 human lung-adenocarcinoma cell line using TCSG.**

**See Supplementary File 2**

**Supplementary Table 3: (A) All human N-glycopeptides identified in tumor xenograft and 2D-grown A549 cells using TCSG. (B) All mouse N-glycopeptides identified in tumor xenograft and normal mouse lungs using TCSG.**

**See Supplementary File 3**

**Supplementary Table 4: (A) N-glycosylated mouse proteins identified in tumor xenograft using TCSG. (B) N-glycosylated human proteins identified in tumor xenograft using TCSG.**

**See Supplementary File 4**

**Supplementary Table 5: (A) All mouse N-glycoproteins identified in tumor xenograft and normal mouse lungs obtained from healthy mice using TCSG. (B) All human N-glycoproteins identified in tumor xenograft and 2D-grown A549 cells using TCSG.**

**See Supplementary File 5**

**Supplementary Table 6: (A) Mouse-stromal N-glycoproteins identified in tumor xenograft by at least a single species-specific N-glycosylated peptide. (B) Human-parenchymal N-glycoproteins identified in tumor xenograft by at least a single species-specific N-glycosylated peptide.**

**See Supplementary File 6**

**Supplementary Table 7: (A) N-glycoproteins identified in normal mouse lungs by at least a single species-specific N-glycosylated peptide. (B) N-glycoproteins identified in 2D-grown A549 human lung-denocarcinoma cell line by at least a single species-specific N-glycosylated peptide.**

**See Supplementary File 7**

**Supplementary Table 8: (A) Stroma-uniqe mouse proteins revealed by subtractive TCSG analysis. (B) Parenchyma-uniqe human proteins revealed by subtractive TCSG analysis.**

**See Supplementary File 8**

**Supplementary Table 9: (A) Stroma-specific protein panel uncovered by compartive subtractive TCSG analysis. (B) Parenchyma-specific protein panel uncovered by compartive subtractive TCSG analysis. (C) A549-specific protein panel uncovered by compartive subtractive TCSG analysis. (C) Normal lung-specific protein panel uncovered by compartive subtractive TCSG analysis.**

**See Supplementary File 9**

**Supplementary Table 10: Diferently regulated orthologues detected in both tumor parenchyma and tumor stroma by at least one species-specific N-glycosylated peptide.**

**See Supplementary File 10**

**Supplementary Table 11: (A) Top parenchymal functional protein networks in terms of statistical significance and number of detected interacting protein species. (B) Top stromal functional protein networks in terms of statistical significance and number of detected interacting protein species.**

**See Supplementary File 11**
